# Supplementary material for: Microenvironment regulation breaks the Faradaic efficiency-current density trade-off for electrocatalytic deuteration using D2O
Source: Nat Commun. 2024 Jun 19;15:5231. doi: 10.1038/s41467-024-49544-y (PMC11187139; doi:10.1038/s41467-024-49544-y)
Supplement: Supplementary file 1 — Supplementary Information [file 41467_2024_49544_MOESM1_ESM.pdf]

Supplementary Information

Microenvironment regulation breaks the Faradaic efficiency-current density trade-off for electrocatalytic deuteration using D_2O

He et al.

Contents

Supplementary Fig. 1 Representative drug molecules.
Supplementary Fig. 2 Time–temperature/energy curve in AIMD.
Supplementary Fig. 3 Transport of **1a** in solution.
Supplementary Fig. 4 Simulation calculations of nanotips.
Supplementary Fig. 5 Electrochemical reduction of CuO NTs to Cu NTs.
Supplementary Fig. 6 Synthesis and characterization of the Cu NTs.
Supplementary Fig. 7 Synchrotron radiation characterization of the Cu NTs.
Supplementary Fig. 8 Standard calibration curves for quantitative analysis of the model reaction with dodecane as an internal standard.
Supplementary Fig. 9 Potential-dependent yield and FE of **2a**.
Supplementary Fig. 10 Surfactants.
Supplementary Fig. 11 Electrochemical performance of **2a** under different conditions.
Supplementary Fig. 12 SEM and TEM images of Cu NRs and Cu NSs.
Supplementary Fig. 13 Active site tests of different Cu nanocatalysts.
Supplementary Fig. 14 Schematic illustration of the flow reactor.
Supplementary Fig. 15 Characterization of the Cu NTs after the deuteration reaction.
Supplementary Fig. 16 In situ ATR-SEIRAS and XPS spectra.
Supplementary Fig. 17 In situ ATR-FTIR spectra under various bias potential.
Supplementary Fig. 18 Contact angle test of Cu NTs with and without BTAB.
Supplementary Fig. 19 Interfacial properties from EIS.
Supplementary Fig. 20 Equivalent circuit diagram.
Supplementary Fig. 21 LSV curves of Cu NTs in a 0.5 M D_2O solution of K_2CO_3.
Supplementary Fig. 22 CV curves of Cu NTs with **1a** with or without BTAB.
Supplementary Fig. 23 Interfacial properties from EIS.
Supplementary Fig. 24 Interfacial properties from EIS.
Supplementary Fig. 25 LSV curves of Cu NSs, NRs and NTs with **1a** for activation energy analyses.
Supplementary Fig. 26 In situ Raman spectra for the electrocatalytic deuteration of **1a** over Cu NTs.
Supplementary Fig. 27 Free-energy diagram for the electrocatalytic hydrogenation of **1a** over the Cu NTs.
Supplementary Fig. 28 EPR and HRMS tests.
Supplementary Fig. 29 H/D exchange experiment.
Supplementary Fig. 30 Comparison of methods for the synthesis of *α,β*-DAEPAs.

Supplementary Fig. 31 Standard calibration curves for the quantitative analysis of other reactions with dodecane as an internal standard.

Supplementary Fig. 32 Electrochemical deuteration of different types of organics on different Cu electrocatalysts in D_2O.

Supplementary Fig. 33 Electrohydrogenation of **1a** in a mixed solution of Diox and 0.5 M K_2CO_3 in H_2O (2:5 v/v, 7 mL) over Cu NTs with and without BTAB.

Supplementary Fig. 34 Comparison of electrocatalytic hydrogenation using H_2O and deuteration using D_2O of **1a** over Cu NTs.

Supplementary Table 1-3.

Supplementary Notes 1-23.

NMR spectra.

References (1-20).

Supplementary Figures and Notes

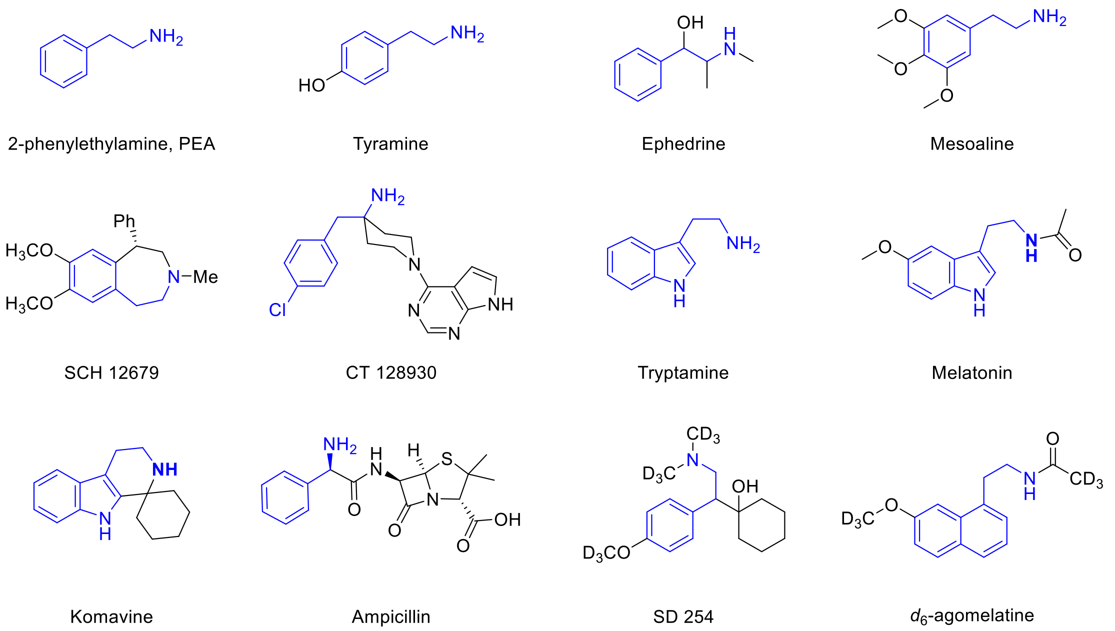

Supplementary Fig. 1 Representative drug molecules. Representative examples of drug molecules containing aryl ethylamine backbones.

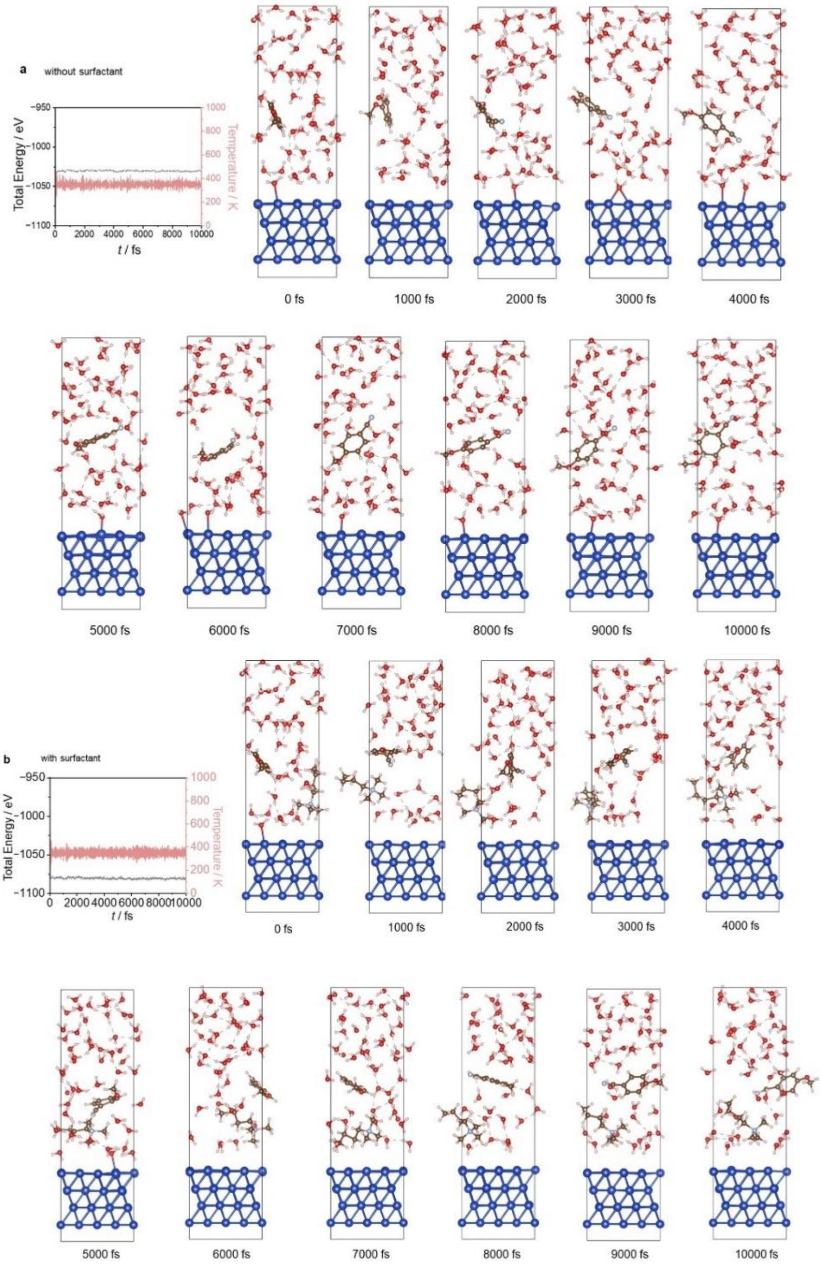

Supplementary Fig. 2 Time−temperature/energy curve in AIMD. Time–temperature/energy curve (a) without BTAB and (b) with BTAB in AIMD.

Supplementary Note 1 To compare the effects of BTAB on the electrode-electrolyte interface, $(H_2O)_4$ clusters were replaced by BTAB molecules. Although the number of H atoms and hydrogen bonds increases in the initial state, after 10000 fs of AIMD simulation, the hydrogen bonds between the H of the CH_3 group and H_2O are difficult to form, causing the breakdown of the hydrogen bond network at the interface and a decrease in the number of hydrogen bonds. In the process of hydrogen bond calculation of the AIMD model, the bond length between H and O and C in the model ranges from 1.2 Å to 4 Å, and the bond angle cut-off is 40 degrees.

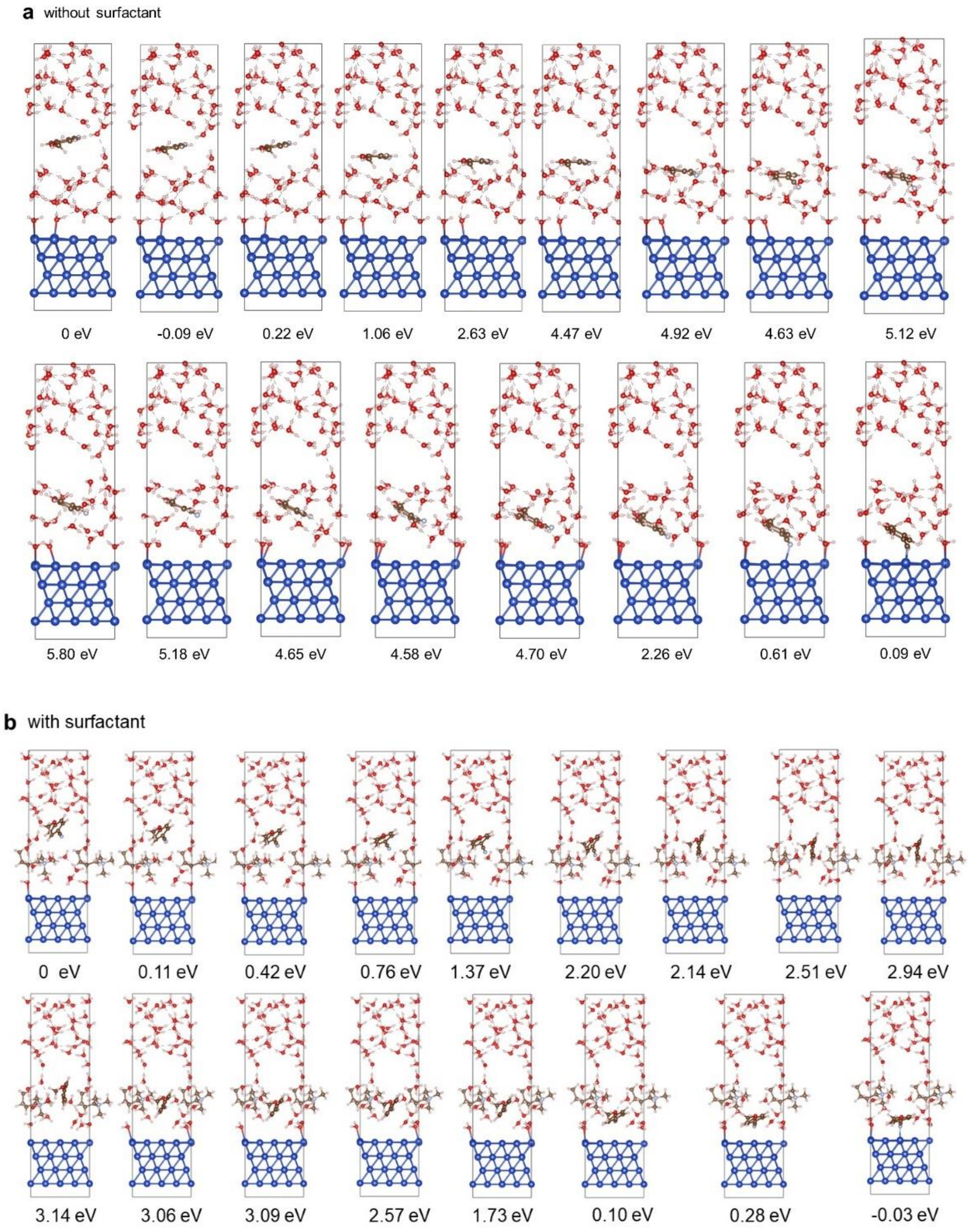

Supplementary Fig. 3 Transport of 1a in solution. Transport of **1a** in solution without (a) and with (b) BTAB.

Supplementary Note 2 The AIMD simulation results reveal that the migration energy barrier of **1a** is reduced, and the migration rate is accelerated due to the disruption of the hydrogen bonding network by the BTAB molecules. This is favourable for the transfer of **1a** to the electrode surface, thus enhancing the deuteration process.

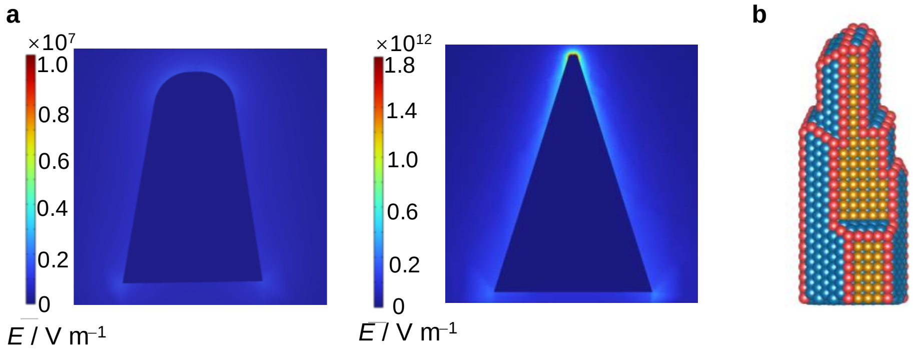

Supplementary Fig. 4 Simulation calculations of nanotips. (a) Electrostatic field on the surface of Cu materials with different tip radii: 50 nm (left) and 5 nm (right). (b) Diagram of the atomic model of the nanotip structure.

Supplementary Note 3 For the nanotip structure formed by fcc metals, such as Cu, the curvature of the needle point leads to a large number of fine crystalline planes converging at the tips, and a large number of crystal prisms are formed. Due to the inherent nature of fcc, its (111) and (001) crystal planes are the crystal planes with the lowest surface energy, and its pinpoint structure is composed of a large number of (111) and (001) crystal planes, as shown in Supplementary Fig. 4b. The blue atoms represent the (111) crystal planes, the yellow atoms represent the (001) crystal planes, and the red atoms represent the crystal prism sites. Here, by measuring the dihedral angle of the relaxed model, we define the angles of (111)/(111) as 77 degrees, (100)/(100) as 96 degrees, and (111)/(100) as 126.9 degrees.

Finite element method simulations. Nernst–Planck–Poisson (NPP) calculations of the Gouy–Chapman model were employed to elucidate the underlying mechanisms of charge transfer and storage, as well as of ion diffusion, governed by the Poisson equation and the Nernst–Plank equation,[1–3] where ε_0 is the permittivity of vacuum, ε_r is the relative permittivity of the medium, φ is the electrostatic potential, D_i is the diffusivity of chemical species i, C_i is the density of the species, z_i is the valency of the species, e is the elementary charge, k_B is Boltzmann's constant, and T is the temperature. We used the 'Electrostatics' and 'Transport of Diluted Species' physics of COMSOL to obtain the electrochemical behavior of all the species. The tip radius of the structure in the panel is 5 nm. The thickness of the Helmholtz layer was taken as the radius of a hydrated potassium ion (0.33 nm),[4] and the absolute temperature T was taken as 297.3 K. The diffusion coefficients D of the potassium ion and the hydroxide ion were taken to be 2.14×10^{-9} m^2 s^{-1} and 5.30×10^{-9} m^2 s^{-1}, respectively, in water.[5] This indicates a

600000-fold increase in the surface-adsorbed BTA^+ ion concentration at the Cu needle tip due to a locally enhanced electrostatic field.

$$\nabla \cdot (\varepsilon_0 \varepsilon_r \nabla \varphi) = -\sum_i z_i e C_i$$

$$\frac{\partial C_i}{\partial t} = \nabla \cdot \left[D_i \nabla C_i + \frac{D_i C_i}{k_B T} z_i e \nabla \varphi \right]$$

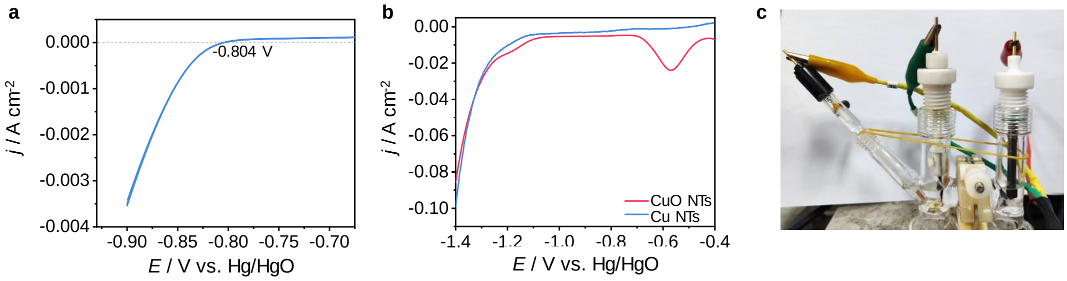

Supplementary Fig. 5 Electrochemical reduction of CuO NTs to Cu NTs. (a) Cyclic voltammetry curves for Hg/HgO electrode calibration in 0.1 M KOH solution. (b) Linear sweep voltammetry (LSV) curves of the Cu NTs and CuO NTs at a scan rate of 5 mV s^{-1} in 0.5 M K_2CO_3 solution. (c) Reaction setup for the electrochemical deuteration reaction.

Supplementary Note 4 The electroreduction of CuO to metallic Cu usually involves a $CuO \rightarrow Cu_2O \rightarrow Cu$ process. The complete disappearance of the cathodic peaks corresponding to Cu(II) and Cu(I) in the LSV curve (blue curve) reveals the successful conversion of CuO to Cu NTs.

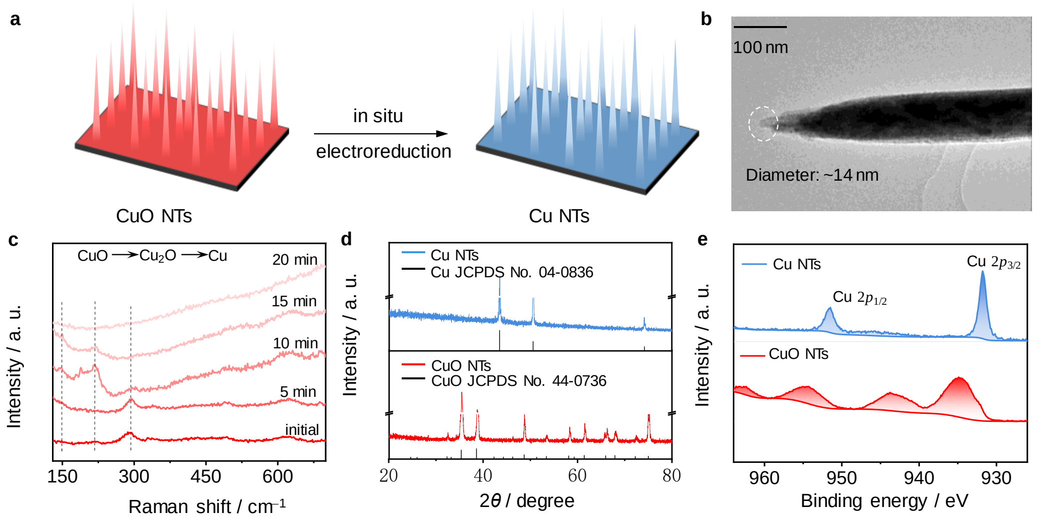

Supplementary Fig. 6 Synthesis and characterization of the Cu NTs. (a) The synthesis of self-supported Cu NTs via an electroreduction strategy. (b) TEM image of Cu NTs. (c) In situ Raman spectra of the Cu NTs collected at −1.0 V in 0.5 M K_2CO_3. (d) XRD patterns of CuO NTs and Cu NTs. (e) Cu 2*p* XPS spectra of CuO and Cu NTs.

Supplementary Note 5 In situ Raman spectra reveal the electrochemical reduction conversion process of $CuO \rightarrow Cu_2O \rightarrow Cu$. The X-ray diffraction (XRD) pattern indicates that all the peaks of the electroreduced sample can be indexed to pure metallic Cu (JCPDS No. 04-0836). To determine the real chemical valence state of the Cu NTs, the sample was treated with argon ion etching 3 times (20 nm) before being subjected to XPS test. The peaks in the X-ray photoelectron spectroscopy (XPS) spectra located at 951.5 eV and 931.8 eV belong to Cu^0 $2p_{1/2}$ and Cu^0 $2p_{3/2}$, respectively.

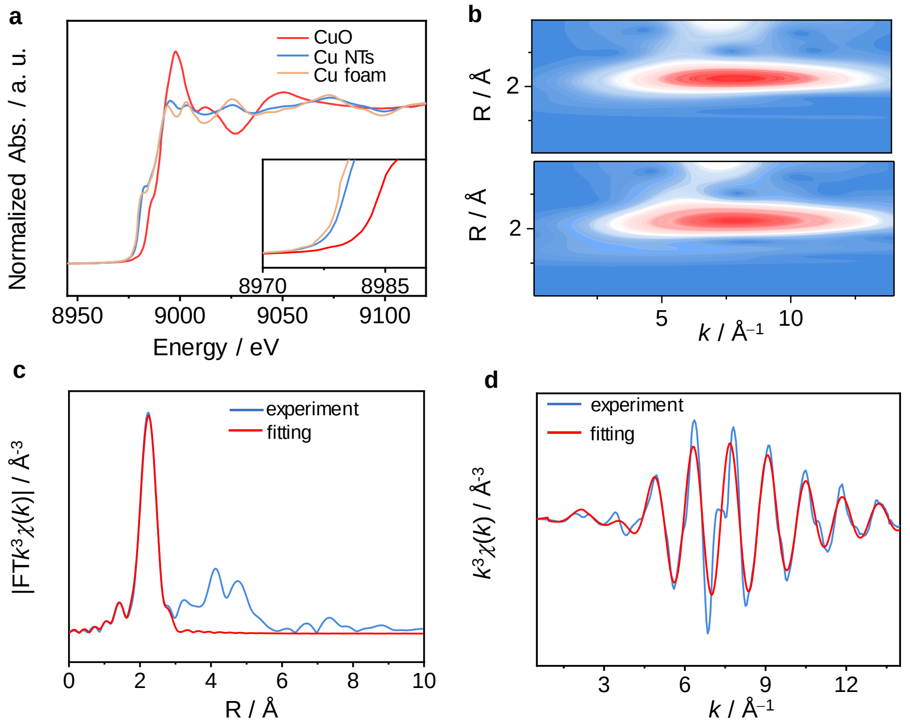

Supplementary Fig. 7 Synchrotron radiation characterization of the Cu NTs. (a) Cu K-edge XANES spectra. (b) 2D contour plots of the WT and (c) Fourier transform (FT) to R-space to isolate the EXAFS contributions from each coordination shell. (d) The χ(k) data are weighted by k^3. The quantified fitting results are shown in Supplementary Table 1.

Supplementary Note 6 To determine the electronic structure and coordination environment of the Cu NTs, X-ray absorption near-edge structure (XANES) and extended X-ray absorption fine structure (EXAFS) data were examined. The Cu NTs samples were ultrasonicated from the Cu foam and then loaded on a carbon paper substrate for synchrotron radiation tests using transmission mode.

The absorption edge position of the Cu NTs is located between that of the CuO NTs and the Cu foil (Supplementary Fig. 7a), indicating a higher valence state of the Cu NTs owing to the presence of low-coordination sites. The EXAFS spectra were subjected to continuous Cauchy wavelet transform (WT), and similar spectra for the Cu NTs and Cu foil further indicate the complete transformation of CuO to Cu (Supplementary Fig. 7b). The Fourier transformed k^3-weighted Cu-K edge EXAFS spectra reveal the new appearance of the Cu−Cu path in the reduced sample (approximately 2.23 Å), while the average Cu−Cu coordination shell of the Cu NTs is much shorter than that of the Cu foam (Fig. 3b). The Fourier transform of the Cu K-edge in the R space plot was fitted with the least-squares method to precisely determine

the average coordination number (CN). The fitting results (Supplementary Figs. 7c-d and Supplementary Table 1) show that the Cu−Cu CN of the Cu NTs is 7.3, which is much smaller than that of the Cu foil (CN = 12). The above results indicate the successful preparation of Cu NTs via the electroreduction of CuO NTs.

For comparison, Cu NTs with fewer coordination-unsaturated sites were also synthesized according to the procedure described below. The prepared CuO NTs were placed in a porcelain boat in a tube furnace and heated at 350 °C for 2 h at a heating rate of 1 °C/min under a H_2/Ar (3:97) atmosphere. The products were cooled to room temperature under a H_2/Ar atmosphere and collected as the final samples, which were denoted as Cu-H_2 NTs.

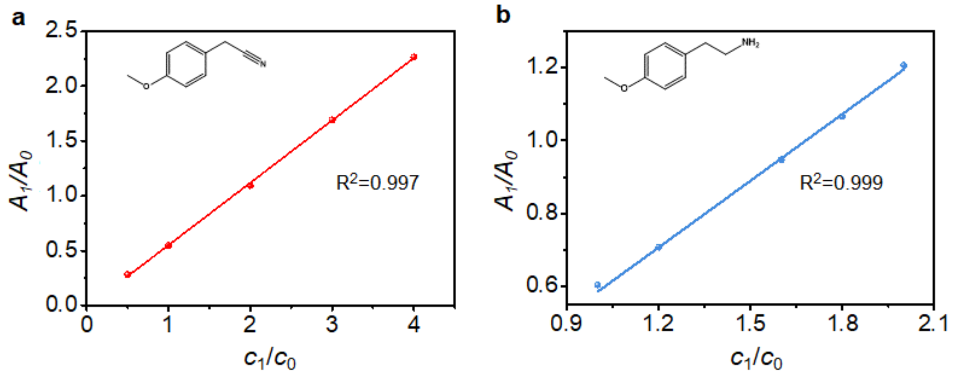

Supplementary Fig. 8 Standard calibration curves for quantitative analysis of the model reaction with dodecane as an internal standard. (a) The reactant *p*-methoxyphenylacetonitrile and (b) the hydrogenated product *p*-methoxyphenethylamine.

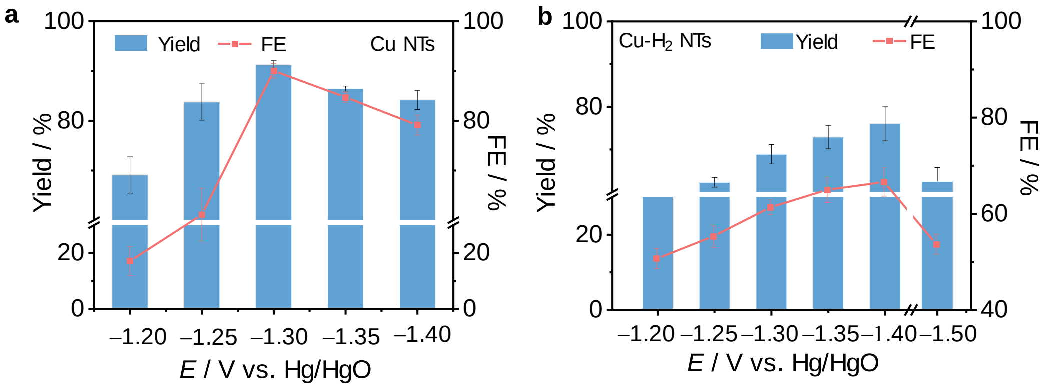

Supplementary Fig. 9 Potential-dependent yields and FEs of 2a. Potential-dependent yields and FEs of **2a** over (a) Cu NTs and (b) Cu-H_2 NTs.

Supplementary Note 7 Both the yield and FE of **2a** are higher for Cu NTs than for Cu-H_2 NTs at the same potentials (Supplementary Fig. 9). This result demonstrates the promoting effect of more low-coordination sites on the transformation of **1a**.

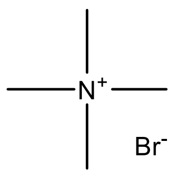

Tetramethylammonium bromide (TMAB)

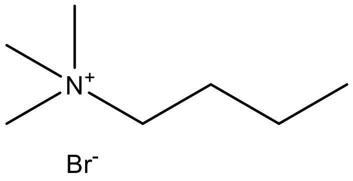

Butyl trimethyl ammonium bromide (BTAB)

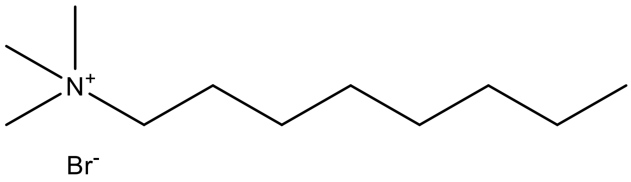

Octyl trimethyl ammonium bromide (OTAB)

Supplementary Fig. 10 Surfactants. Structures of different quaternary ammonium surfactants with the same head groups but different tail chains (TMAB, BTAB, OTAB).

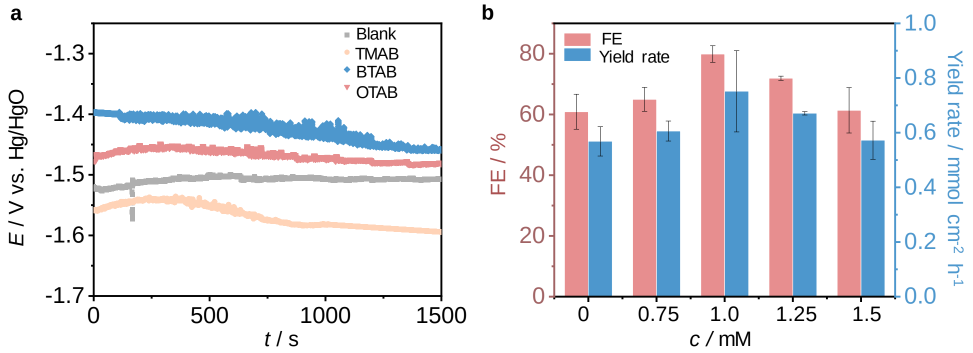

Supplementary Fig. 11 Electrochemical performance of 2a under different conditions. (a) Chronopentiometric curves of the electrocatalytic deuteration of **1a** without surfactant and with different surfactants over Cu NTs at −100 mA cm^{-2}. (b) FEs and yield rates of **2a** under various concentrations of BTAB at −100 mA cm^{-2}.

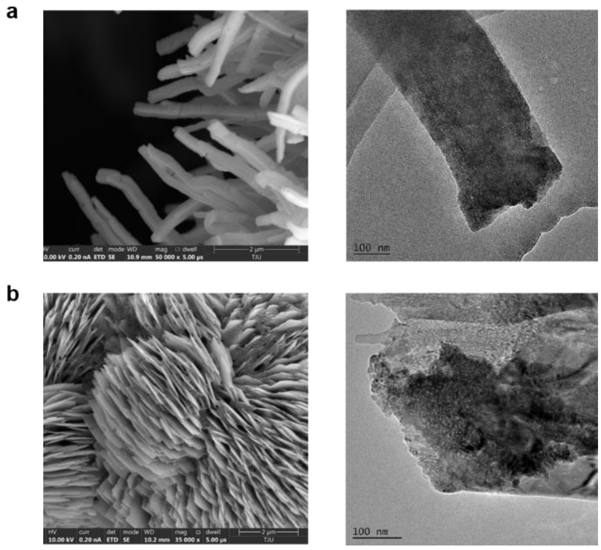

Supplementary Fig. 12 SEM and TEM images of Cu NRs and Cu NSs. SEM (left) and TEM (right) images of (a) Cu NRs and (b) Cu NSs.

Supplementary Note 8

Synthesis of CuO nanorods (NRs). Self-supported CuO NRs were synthesized according to previous methods.[6] First, commercial Cu foam was treated with acetone, 3.0 M HCl solution, and deionized water (DIW). Then, the cleaned copper foam was immersed in an aqueous solution containing 10 M NaOH (7.5 mL), 1 M $(NH_4)_2S_2O_8$ (3 mL), and DIW (19.5 mL) for 3 h at 0 °C. After that, the blue $Cu(OH)_2$ NRs/Cu foam was removed from the solution, washed with DIW and absolute ethanol, and dried at room temperature. Finally, the $Cu(OH)_2$ NR/Cu foam was put into a porcelain boat and heated through a 3-stage annealing process (2 h at 60 °C, 4 h at 120 °C, and 6 h at 180 °C) in an Ar environment to form the CuO NRs structure.

Synthesis of CuO nanosheets (NSs). Self-supported CuO NSs were synthesized according to the reported literature.[7] The fully cleaned Cu foam was submerged in a solution containing 0.133 M $(NH_4)_2S_2O_8$ and 2.667 M NaOH for 4 h without interruption, followed by thermal annealing at 250 °C in air for 2 h to synthesize CuO NSs.

In situ electrochemical synthesis of Cu NRs and NSs. The Cu NRs and NSs were synthesized by a method similar to that used for the synthesis of Cu NTs. However, Cu NRs and NSs were obtained by electroreduction of CuO at −1.0 V vs. Hg/HgO for 45 min to ensure complete reduction.

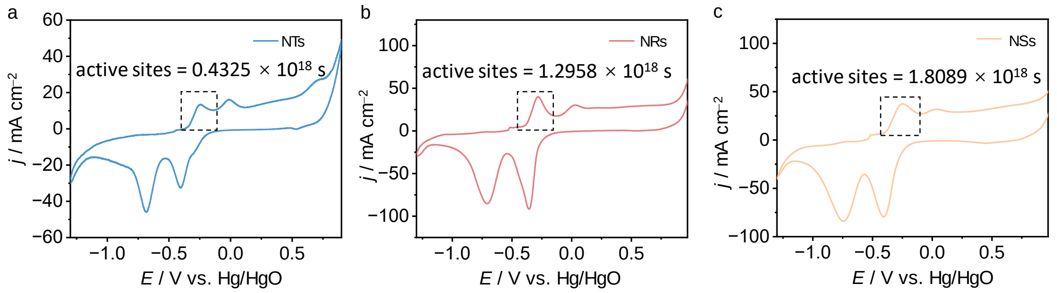

Supplementary Fig. 13 Active site tests of different Cu nanocatalysts. CV curves of (a) Cu NTs, (b) Cu NRs, and (c) Cu NSs at scan rates of 10 mV s^{-1} in 0.5 M K_2CO_3 (D_2O).

Supplementary Note 9 Calculating the active sites by the redox peak area of a metal is a simple and convenient method.[8] Here, we measured the redox peak areas of the Cu NTs, NRs, and NSs and calculated the number of active sites in the three samples (Supplementary Figs. 13a-c). To verify the intrinsic activity of the catalysts, the performances of the three Cu materials were normalized to the number of active sites. The calculated active site areas of the Cu NTs, Cu NRs, and Cu NRs were 0.4325 × 1018 s, 1.2958 × 1018 s, and 1.8089 × 1018 s, respectively.

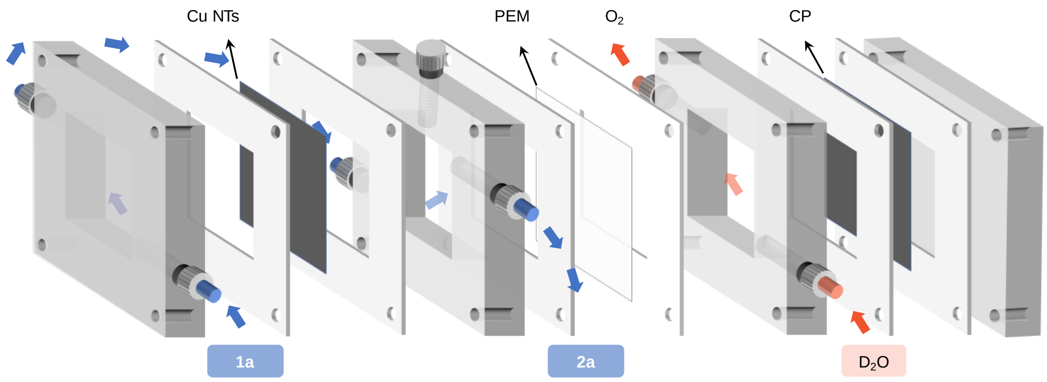

Supplementary Fig. 14 Schematic illustration of the flow reactor. Schematic illustration of the flow reactor used for the electrochemical deuteration of **1a** using D_2O.

Supplementary Note 10

Stability measurement in a flow cell. The stability was measured in a typical flow cell that was separated by a Nafion 117 proton exchange membrane consisting of Cu NTs as the working electrode, CP as the counter electrode, and Hg/HgO as the reference electrode. Thirty milliliters of a 0.5 M D_2O solution of K_2CO_3 was added to the anodic cell, and a mixed solution of 20 mL of Diox and 15 mL of a 0.5 M D_2O solution of K_2CO_3 that contained 1.0 mM BTAB was added to the cathodic cell. **1a** was added to the cathodic cell at 30-hour intervals (for a total of **1a**). Chronoamperometry was carried out at −100 mA cm^{-2}. A peristaltic pump was used to achieve liquid-phase circulation, and the flow rate was 1 mL/min. Supplementary Fig. 14 shows a schematic diagram of the flow cell. The working electrode was held in the cathodic cell by conductive copper tape before performance testing.

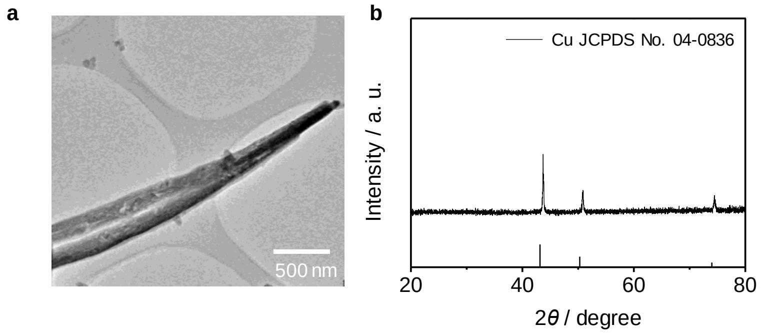

Supplementary Fig. 15 Characterization of the Cu NTs after the deuteration reaction. (a) TEM image and (b) XRD pattern of the Cu NTs.

Supplementary Note 11 TEM images show that the tip structure of the Cu NTs is well maintained, and all the diffraction peaks in the XRD pattern can be indexed to the (111), (200), and (220) lattice planes of Cu (JCPDS No. 04-0836), revealing the good stability of the Cu NTs.

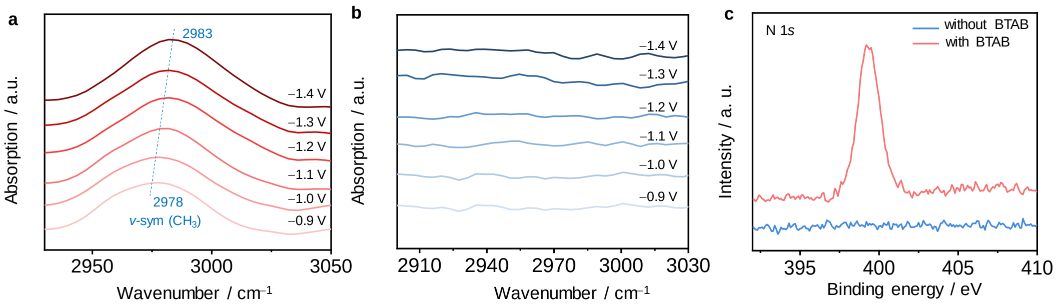

Supplementary Fig. 16 In situ ATR-SEIRAS and XPS spectra. In situ ATR-SEIRAS under various bias potentials (a) with and (b) without Cu NTs. (c) XPS spectra of the Cu NTs with and without BTAB treatment.

Supplementary Note 12 To exclude interactions between Au and the BTAB surfactant, we carried out ATR-FTIR experiments without loading Cu NTs under other identified conditions. As shown in Supplementary Fig. 16b, no obvious signal was observed under various bias potentials. This indicates that the observed signal in Supplementary Fig. 16a is ascribed to the interaction between BTAB and Cu rather than between BTAB and Au.

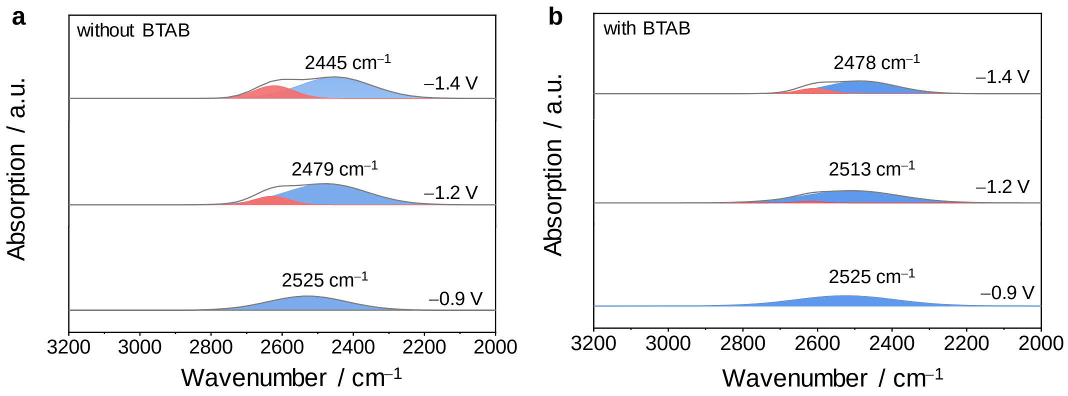

Supplementary Fig. 17 In situ ATR-FTIR spectra under various bias potential. In situ ATR-FTIR spectra under various bias potentials for the system (a) without or (b) with BTAB over Cu NTs.

Supplementary Note 13 Smaller redshifts of the ν_{O-D} mode (2478 vs. 2445 cm^{-1} and 2479 vs. 2513 cm^{-1}) after adding BTAB to the electrolyte suggest weaker adsorption of D_2O on the Cu NTs.

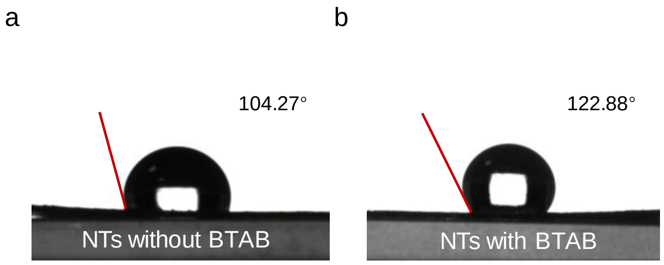

Supplementary Fig. 18 Contact angle test. Contact angle results of Cu NTs (a) with BTAB and (b) without BTAB.

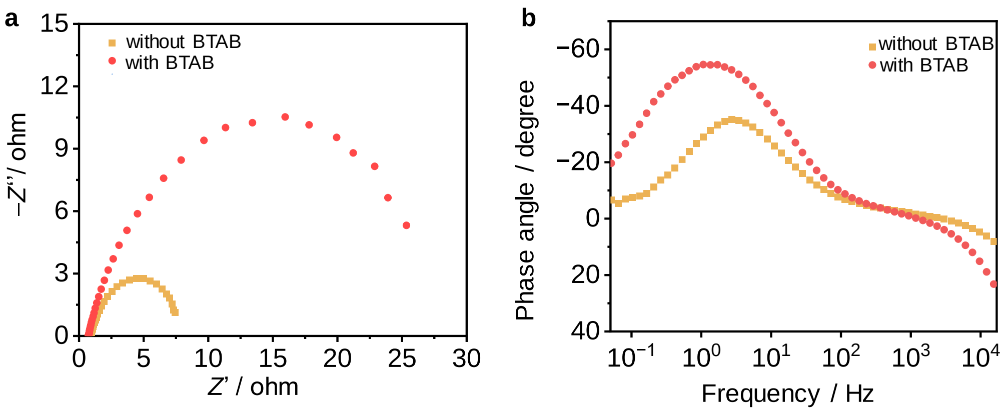

Supplementary Fig. 19 Interfacial properties from EIS. Comparison of (a) Nyquist plots and (b) Bode plots for the DER process with and without 1.0 mM BTAB over Cu NTs at −1.15 V.

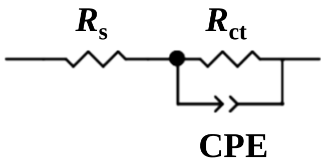

Supplementary Fig. 20 Equivalent circuit diagram. The equivalent circuit of all the systems.

Supplementary Note 14 ZView2 software was used to examine the data using the circuit shown above, where R_s is the solution resistance and R_{ct} is the charge transfer resistance. CPE: Constant-Phase Element.

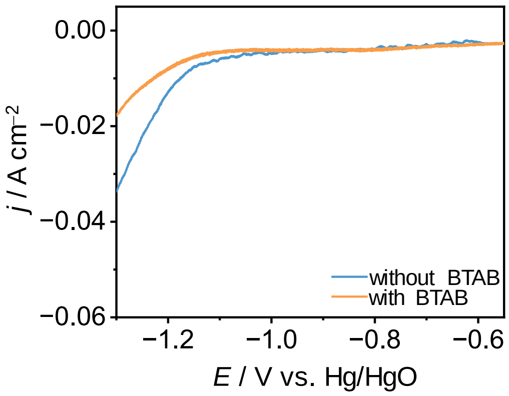

Supplementary Fig. 21 LSV curves of Cu NTs in a 0.5 M D_2O solution of K_2CO_3. LSV curves of Cu NTs in a 0.5 M D_2O solution of K_2CO_3 with and without BTAB.

Supplementary Note 15 A remarkable increase in the onset potential and a more negative potential required to achieve a current density of −10 mA cm^{-2} over Cu NTs after adding BTAB to the electrolyte imply poor activity for D_2 formation.

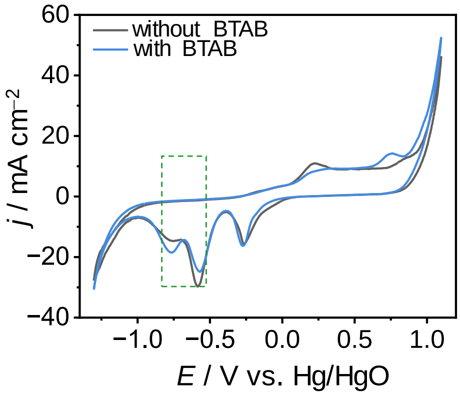

Supplementary Fig. 22 CV curves of Cu NTs with 1a with or without BTAB. CV curves of Cu NTs with **1a** at a scan rate of 3 mV s^{-1} in Diox/0.5 M K_2CO_3 with or without BTAB.

Supplementary Note 16 The reduction peak of **1a** is larger with BTAB than that without BTAB, suggesting that more **1a** is present near the electrode surface. The increased concentration of **1a** may be ascribed to the breaking of the deuterium bonding networks between D_2O molecules by BTAB, thus accelerating the migration of **1a** to the electrode surface.

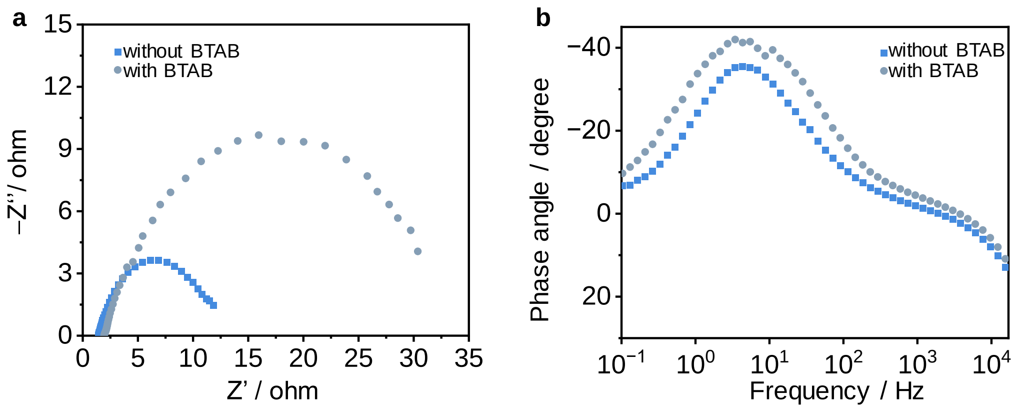

Supplementary Fig. 23 Interfacial properties from EIS. Comparison of (a) Nyquist plots and (b) Bode plots for the deuteration of **1a** with and without 1.0 mM BTAB over Cu NTs at −1.15 V.

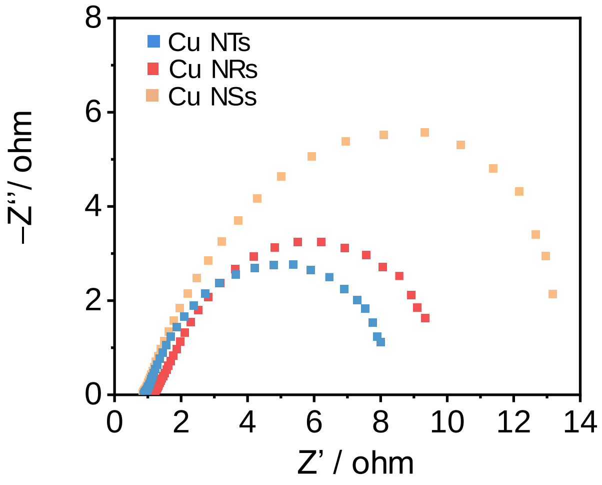

Supplementary Fig. 24 Interfacial properties from EIS. EIS Nyquist plots at −1.3 V for the Cu NTs, Cu NRs, and Cu NSs.

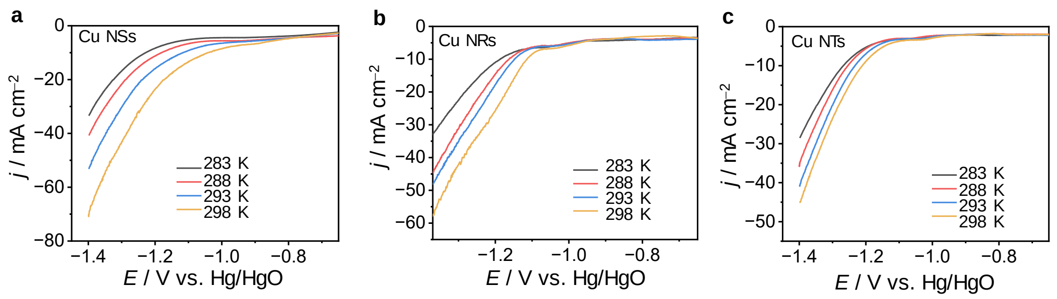

Supplementary Fig. 25 LSV curves of Cu NSs, NRs, and NTs with 1a for activation energy analyses. Polarization curves of (a) Cu NSs, (b) Cu NRs, and (c) Cu NTs in a 0.5 M K_2CO_3 aqueous electrolyte with BTAB at 10-25 °C.

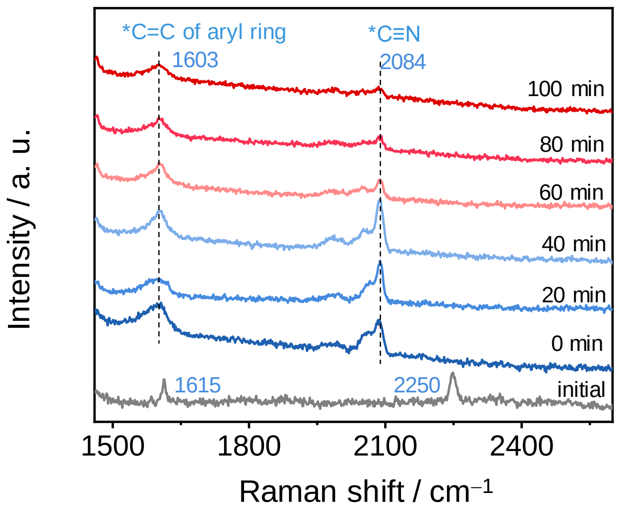

Supplementary Fig. 26 In situ Raman spectra for the electrocatalytic deuteration of 1a over Cu NTs. In situ Raman spectra of a mixed solution of Diox/0.5 M K_2CO_3 with BTAB for the electrocatalytic deuteration of 1a over Cu NTs at −1.3 V.

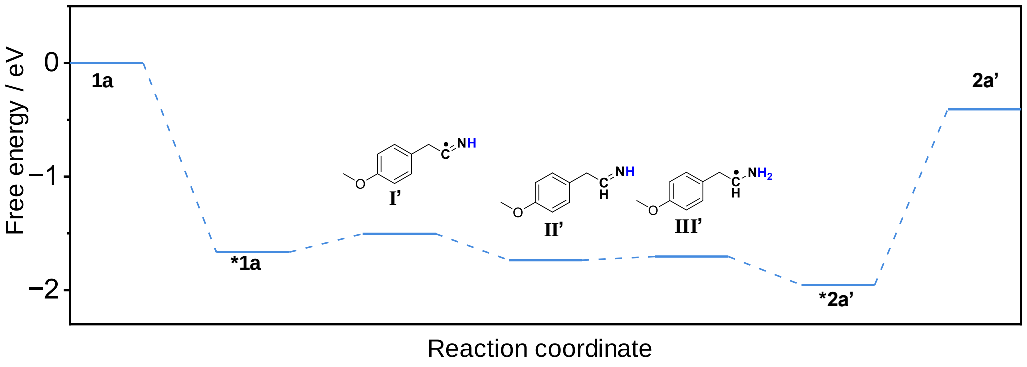

Supplementary Fig. 27 Free-energy diagram for the electrocatalytic hydrogenation of 1a. Free-energy diagram for the electrocatalytic hydrogenation of **1a** over the Cu NTs.

Supplementary Note 17 According to our proposed mechanism, the hydrogenation of **1a** using H_2O is simulated. The much lower hydrogenation barrier of imine than that of **1a** implies easier hydrogenation of the imine intermediate over the Cu NTs.

Computational details. All the DFT calculations were performed using the Vienna ab initio simulation package (VASP).[9] The projector augmented wave (PAW)[10] pseudopotential with the PBE[20] generalized gradient approximation (GGA) exchange-correlation function was utilized in the computations. The cut-off energy of the plane wave basis set was 500 eV, and Monkhorst-Pack meshes of 3 × 3 × 1 and 7 × 7 × 1 were used in *K*-sampling to calculate the adsorption energy and density of states (DOS). The long-range dispersion interaction was described by the DFT-D3 method. The electrolyte was incorporated implicitly with the Poisson-Boltzmann model implemented in VASPsol.[12] The relative permittivity of the media was chosen to be ϵ_r = 78.4, corresponding to that of water. All the structures were spin-polarized, all the atoms were fully relaxed with an energy convergence tolerance of 10^{-5} eV per atom, and the final force on each atom was < 0.05 eV $Å^{-1}$. The TS searches were performed using the dimer method in the VTST package. The final force on each atom was < 0.05 eV $Å^{-1}$. The TS search was conducted by using the climbing-image nudged elastic band (CI-NEB) method to generate initial guess geometries, followed by the dimer method to converge to the saddle points.

In this work, face-centered cubic (fcc) phase copper (Cu) was used (a = b = c = 3.621 Å, $\alpha = \beta = \gamma$ = 90.000°, Fm-3m). The bulk model of the sample was built by a 4 × 4 supercell of Cu (111) with four layers of Cu. For the Cu model with low coordination, 3 Cu atoms need to be removed from the surface of the Cu (111) model. All periodic slabs have a vacuum layer of at least 15 Å. The adsorption energy of the reaction

intermediates can be computed using equations (7)–(8):

$$\Delta E = E_{(ads/slab)} - E_{ads} - E_{slab} \quad (7)$$

$$\Delta G = \Delta E + \Delta E_{ZPE} - T\Delta S \quad (8)$$

where ΔE_{ZPE} is the zero-point energy change and ΔS is the entropy change. In this work, the values of ΔE_{ZPE} and ΔS were obtained via vibration frequency calculations. The energy of the reaction can be calculated by the following fourteen equations (9) - (19):

$*+H_2O$——$*H_2O$ (9)

$*H_2O+e^-$——$*H+OH^-$ (10)

$*H+*H$——$*+H_2$ (11)

$*+CH_3CN$——$*CH_3CN$ (12)

$*CH_3CH_2NH_2$——$*+ CH_3CH_2NH_2$ (13)

$*+C_9H_9NO$——$*C_9H_9NO$ (14)

$*C_9H_9NO+H_2O+e^-$——$*C_9H_9NHO+OH^-$ (15)

$*C_9H_9NHO+H_2O+e^-$——$*C_9H_{10}NHO+OH^-$ (16)

$*C_9H_{10}NHO+H_2O+e^-$——$*C_9H_{10}NH_2O+OH^-$ (17)

$*C_9H_{10}NH_2O+H_2O+e^-$——$* C_9H_{11}NH_2O+OH^-$ (18)

$*C_9H_{11}NH_2O$——$*+ C_9H_{11}NH_2O$ (19)

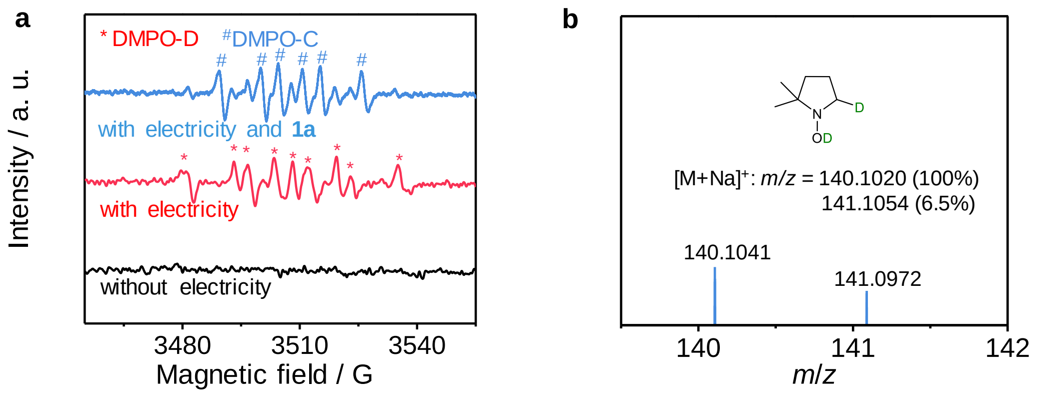

Supplementary Fig. 28 EPR and HRMS tests. (a) EPR experiments for trapping deuterium (*) and carbon (#) radicals during the electrocatalytic deuteration of **1a** over Cu NTs. (b) HR-MS analysis of the deuterium radical in the spin-trapping experiment.

Supplementary Note 18

Electron paramagnetic resonance (EPR) experiments for the electrocatalytic deuteration of 1a. EPR measurements were performed in a divided three-electrode electrochemical cell by using the trapping agent 5,5-dimethyl-1-pyrroline-*N*-oxide (DMPO). After chronoamperometry was performed with and without **1a** at −1.3 V for 10 min, 0.5 mL of reaction solution close to the working electrode was removed, and the solution was added to an ampoule bottle. Then, 0.1 mM DMPO was quickly added to the bottle, and the mixture was stirred to form a homogeneous solution. After that, the solution was removed for the EPR test.

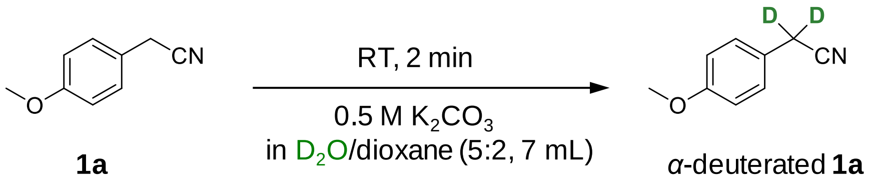

1a

α-deuterated **1a**
96% isolated yield, 99% D content

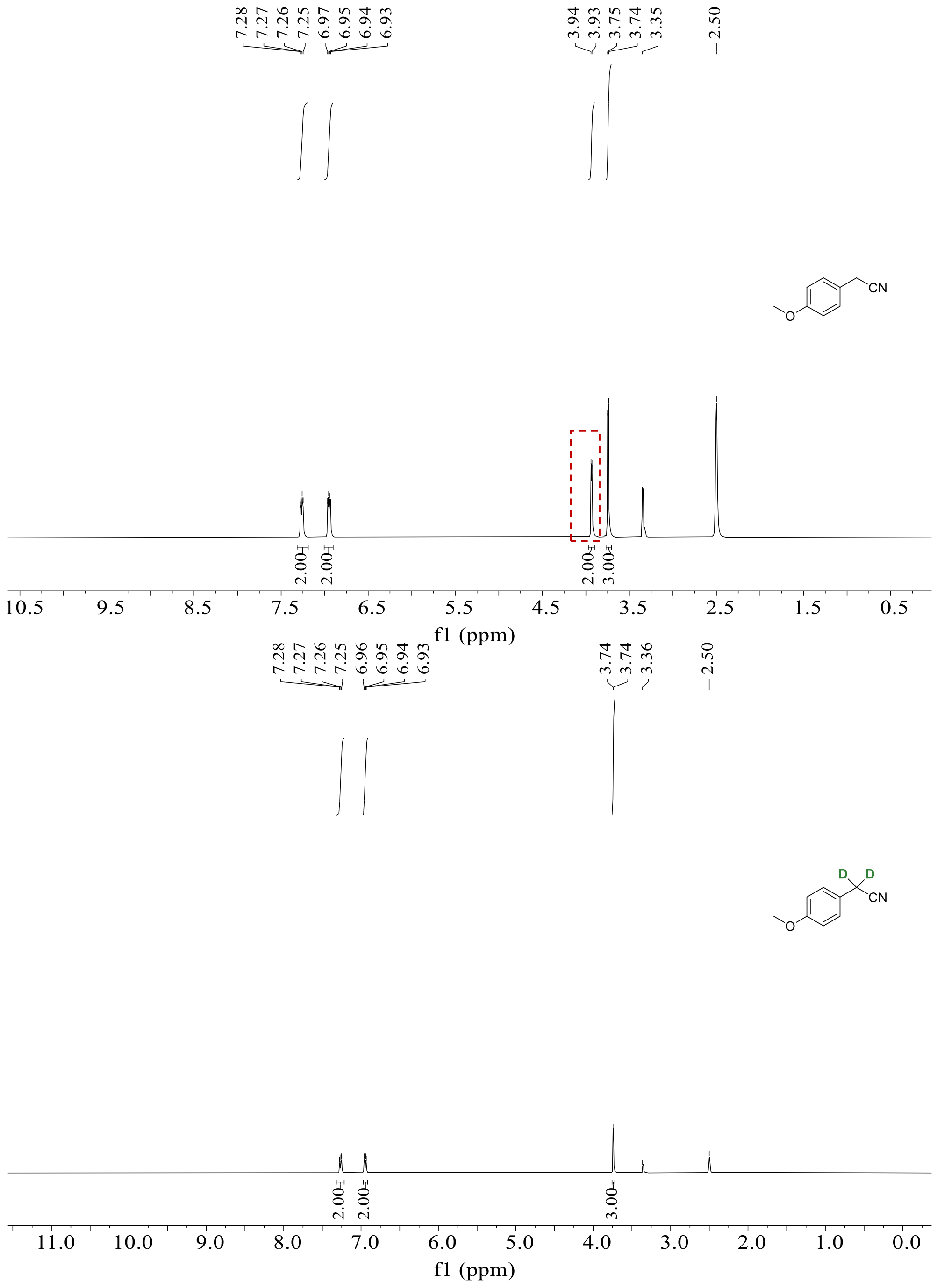

Supplementary Fig. 29 H/D exchange experiment. The *α*-C−H to *α*-C−D exchange of 0.1 mmol of **1a** is very quick with the assistance of K_2CO_3 in D_2O.

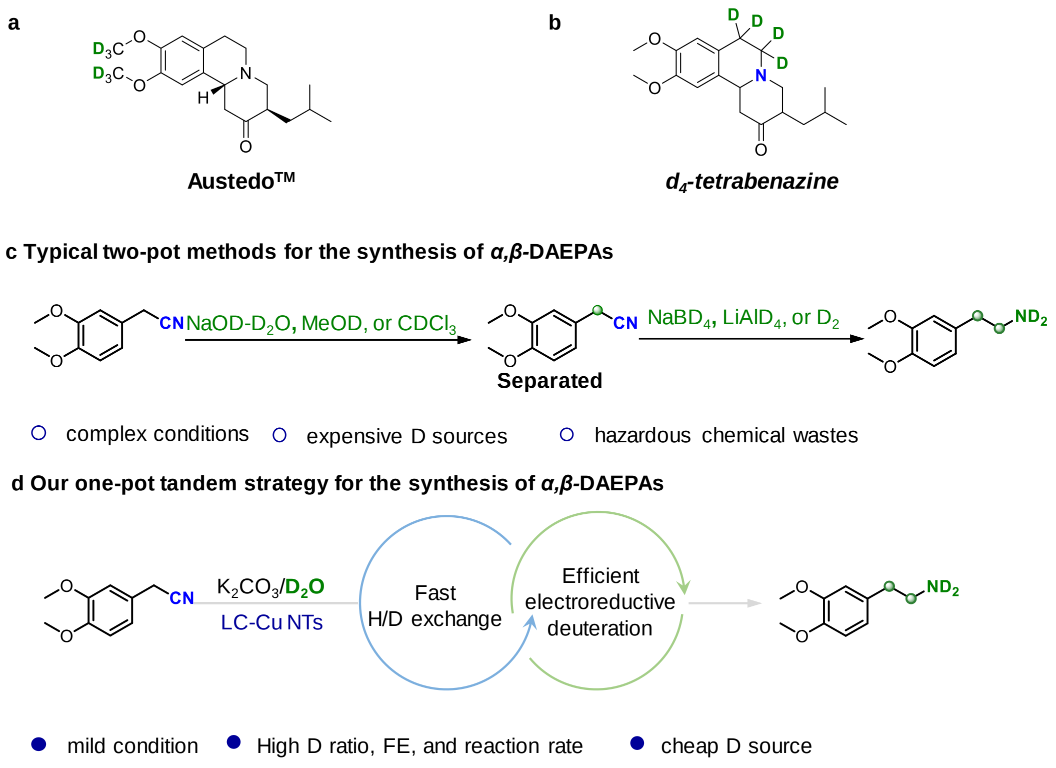

Supplementary Fig. 30 Comparison of methods for the synthesis of *α,β*-DAEPAs. The structures of (a) the first approved deuterated drug, Austedo, and (b) the synthesized *d*4-tetrabenazine in our work. (c) Typical methods and (d) our one-pot deuteration strategy for the synthesis of *α,β*-DAEPAs.

Supplementary Note 19 Comparison of the reported methods for the synthesis of *α,β*-deuterated arylethyl primary amines and our method

(1) "Two-pot" in refs. and "one-pot" in our manuscript. Typically, two-pot reaction systems are adopted to synthesize *α,β*-deuterated arylethyl primary amines via H/D exchange of aryl acetonitrile followed by reductive deuteration of the nitrile group (C≡N) in different reactors.[13–15] The requirement for such two-pot procedures may be due to the poor compatibility of the reaction conditions for the formation and subsequent deuteration of *α*-deuterio aryl acetonitrile for one-pot synthesis.

(2) "Expensive deuterated sources (e.g., $NaBD_4$, $LiAlD_4$, D_2)" in the literature and "inexpensive and safe D_2O" in our manuscript. Despite their dominance, expensive deuterated sources (e.g., $NaBD_4$, $LiAlD_4$, D_2) and strictly controlled anhydrous conditions are often needed. Moreover, the purification of *α*-deuterated aryl acetonitrile is labor-consuming and time-consuming. These intrinsic factors cause operating complexities, safety risks, and environmental issues, restricting their practical application.

Supplementary Note 20

The procedure for the gram-level synthesis of 2b. The gram-level synthesis of **2b** was carried out by using a flow cell, as shown in Supplementary Fig. 14. The Cu NTs, CP, and Hg/HgO were used as the working electrode, counter electrode, and reference electrode, respectively. For the synthesis of **2b**, a mixed solution of Diox/0.5 M K_2CO_3 in D_2O (3:4 v/v, 35 mL) was used as the electrolyte, and then **1b** was added to the electrolyte. The chronoamperometric measurements were carried out at −100 mA cm^{-2} until the starting substrates vanished. After the reaction was complete, the solution was extracted with dichloromethane (DCM). The DCM phase was dried over anhydrous sodium sulfate and removed under vacuum. The residue was analysed by GC to determine the yield.

The synthetic procedure of *d_4-tetrabenazine*. Formic acid (1.4 mmol) was slowly added to the as-prepared **2b** (1.0 mmol) in an ice bath, after which the pale yellow substance was heated to reflux (190 °C) until the reaction was finished. After the solution cooled, the yellow-colored reactant was diluted with toluene (2.0 mL). Then, 2.0 mmol of $POCl_3$ was added to the mixed solution, which was heated to reflux until the end of the reaction. Excess toluene and $POCl_3$ were removed by distillation, and the residue was washed with petroleum ether and dissolved in Diox. The solution was then poured into ice, and the resulting solution was washed with ethyl ether 3 times. The pH of the solution was adjusted to 12, and the solution was extracted with ethyl ether and dried over anhydrous sodium sulfate to give amber-colored oil. The amber oil was treated with 3.0 M cyclopentyl methyl ether hydrochloric acid solution, and the precipitated solid product was filtered. The crude product was mixed with acetone, and an equal amount of 3-(*N,N*-dimethylaminomethyl)-5-methyl-2-hexanone was added dropwise while stirring at room temperature. The precipitated solid was extracted and washed with water to obtain a light yellow crude product, which was purified by thin-layer chromatography (TLC) plates to give the pure product.[16, 17]

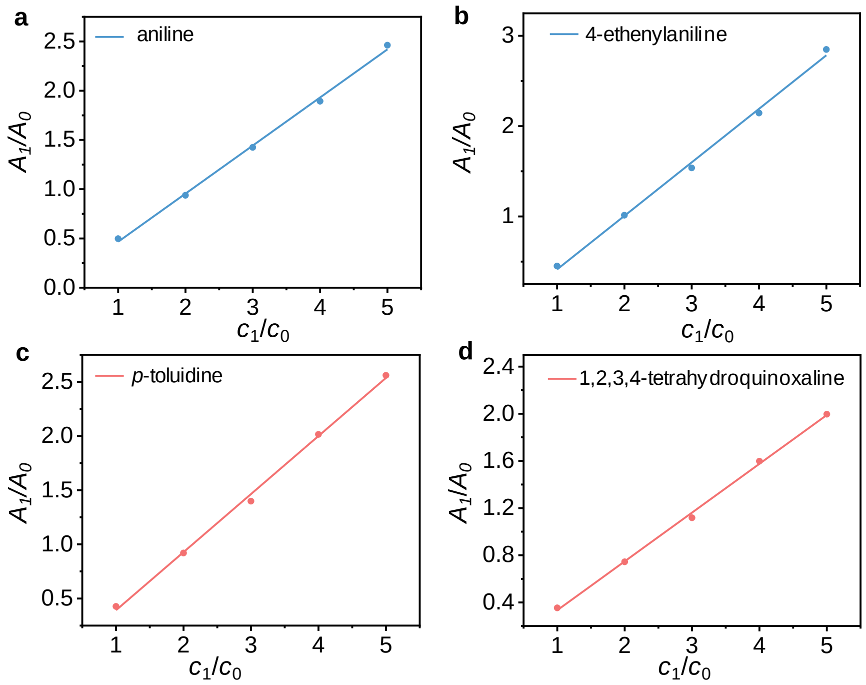

Supplementary Fig. 31 Standard calibration curves for the quantitative analysis of other reactions with dodecane as an internal standard. (a) The hydrogenated product (a) aniline and (b) 4-ethenylaniline and (c) *p*-toluidine and (d) 1,2,3,4-tetrahydroquinoxaline.

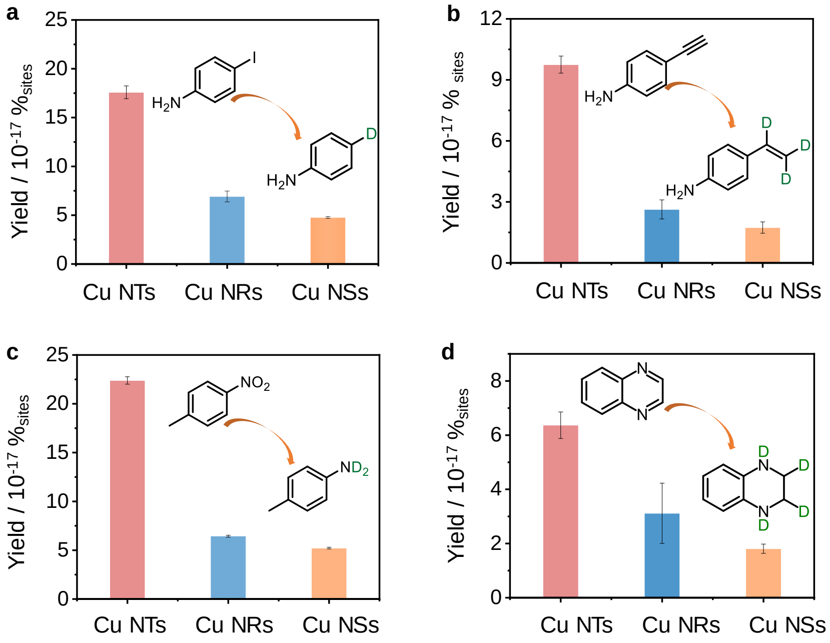

Supplementary Fig. 32 Electrochemical deuteration of different types of organics on different Cu electrocatalysts in D_2O. Electrochemical deuteration of (a) *p*-iodoaniline and (b) *p*-ethnylaniline and (c) 4-nitrotoluene and (d) quinoline on different Cu electrocatalysts in D_2O.

Supplementary Note 21 The active site-normalized yields of different deuteration reactions over Cu NTs, Cu NRs, and Cu NSs. High yields of different deuteration reactions are obtained by using Cu NTs, suggesting good methodology generality.

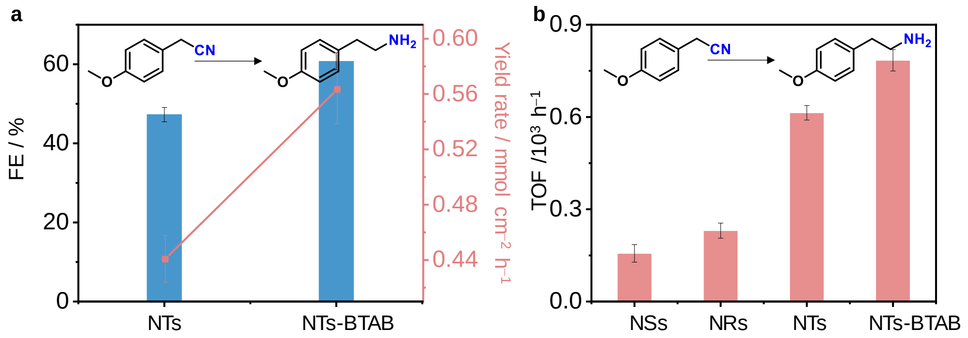

Supplementary Fig. 33 Electrohydrogenation of 1a in a mixed solution of Diox and 0.5 M K_2CO_3 in H_2O (2:5 v/v, 7 mL) over Cu NTs. (a) FE and yield rate of **2a** at −100 mA cm^{-2} over Cu NTs with and without BTAB. (b) TOF values at −100 mA cm^{-2} over Cu NSs, NRs and NTs without or with BTAB.

Supplementary Note 22 As shown in Supplementary Fig. 33a, the combined system can also promote the electrocatalytic hydrogenation of **1a** at −100 mA cm^{-2}. The FE and yield rate obviously increase after the addition of BTAB. Additionally, benefiting from the combination of the nanotip structure and surfactant, the highest turnover frequency (TOF) values are obtained under standard reaction conditions (Supplementary Fig. 33b). These results further demonstrate that promoting mass transfer and establishing a hydrophobic interface microenvironment to inhibit the HER are also effective at improving the FE of hydrogenation reactions at high current densities.

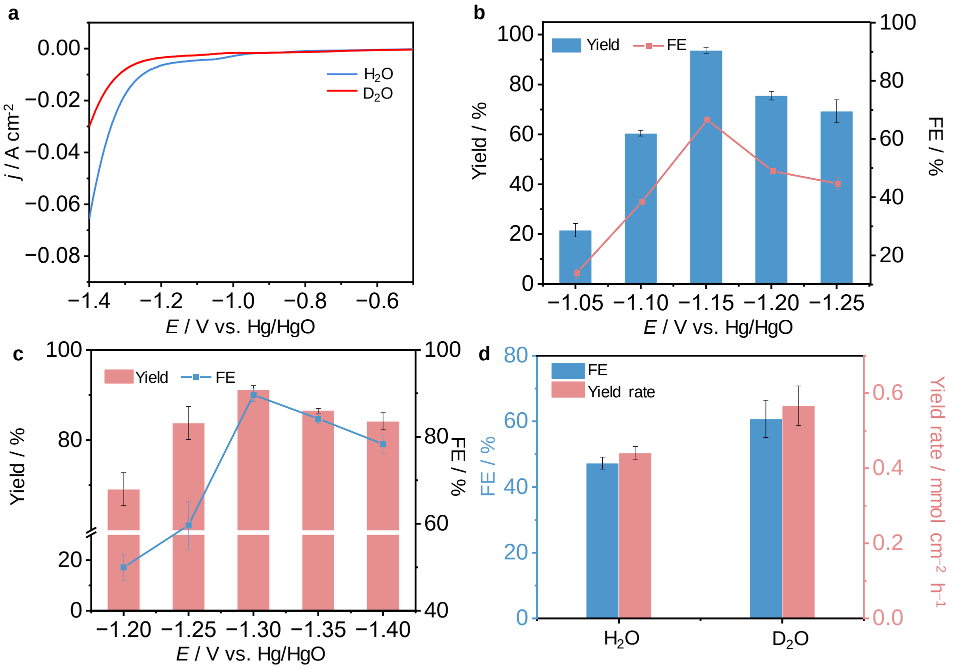

Supplementary Fig. 34 Comparison of electrocatalytic hydrogenation using H_2O and deuteration using D_2O of 1a over Cu NTs. (a) Linear sweep voltammetry (LSV) curves of the Cu NTs cathode recorded in 0.5 M K_2CO_3 in H_2O and D_2O at a scan rate of 10 mVs^{-1}. (b) Potential-dependent yield and FE of 4-methoxyphenethylamine over Cu NTs obtained by using H_2O. (c) Potential-dependent yield and FE of **2a** over Cu NTs by using D_2O. (d) Comparison of FEs and yield rates of 4-methoxyphenethylamine obtained by using H_2O or D_2O at −100 mA cm^{-2}.

Supplementary Note 23 The slower kinetics of D* generation than of H* generation will lead to differences in the overpotential, activity, selectivity, FE, and reaction rate between deuteration reactions and hydrogenation reactions using H_2O. We carried out several experiments to obtain a better understanding of the results. Linear sweep voltammetry (LSV) curves (Supplementary Fig. 34a) revealed that the overpotential of the hydrogen evolution reaction (HER) is much smaller than that of the DER for the same current density, such as the benchmark current density of −10 mA cm^{-2}, which implies sluggish kinetics of DER. In addition, potential-dependent experiments revealed optimal results at −1.15 V vs. Hg/HgO for the hydrogenation of **1a** using H_2O (Supplementary Fig. 34b). In contrast, the deuteration of **1a** using D_2O had the best performance when the potential reached −1.3 V vs. Hg/HgO (Supplementary Fig. 34c). Furthermore, the FE and yield rate of hydrogenation of **1a** are greatly different from those of deuterated 1a under the same reaction conditions (Supplementary Fig. 34d).

Supplementary Table 1 EXAFS fitting parameters at the Cu *K*-edge for various

samples (S_0^2 = 0.86)

Sample	Path	C.N.	R (Å)	σ^2 (Å^2)	ΔE (eV)	R factor
Cu foil	Cu-Cu	12*	2.54±0.01	0.0087	4.4±0.6	0.0042
Cu NTs	Cu-O	0.6±0.1	1.84±0.02	0.0036	3.1±1.0	0.0040
	Cu-Cu	7.3±0.2	2.54±0.01	0.0090		
	Cu-O-Cu	1.5±0.6	3.01±0.03	0.0130		

C.N.: coordination number; R: bond distance; σ^2: Debye–Waller factor; ΔE: inner potential correction; R factor: goodness of fit. * Fitting with fixed parameters.
The k-range of 3-14 Å^{-1} and R-range of 1-3 Å were used for the fitting of Cu foil; the k-range of 3-13 Å^{-1} and R-range of 1-3 Å were used for the fitting of Cu NTs.

Supplementary Table 2 Simulated impedance parameters of the Nyquist plots over the Cu NTs electrode at −1.15 V.

Systems	R_{ct} (Ω)	CPE-T	CPE-P	R_s (Ω)
0.5 M K_2CO_3	7.227	0.030507	0.81347	1.443
0.5 M K_2CO_3+ BTAB	28.62	0.040278	0.72931	0.72931
0.5 M K_2CO_3+ **1a**	30.33	0.010981	0.73072	1.972
0.5 M K_2CO_3+ BTAB+**1a**	10.63	0.017749	0.77940	1.685

Supplementary Table 3 Methodology universality.[a]

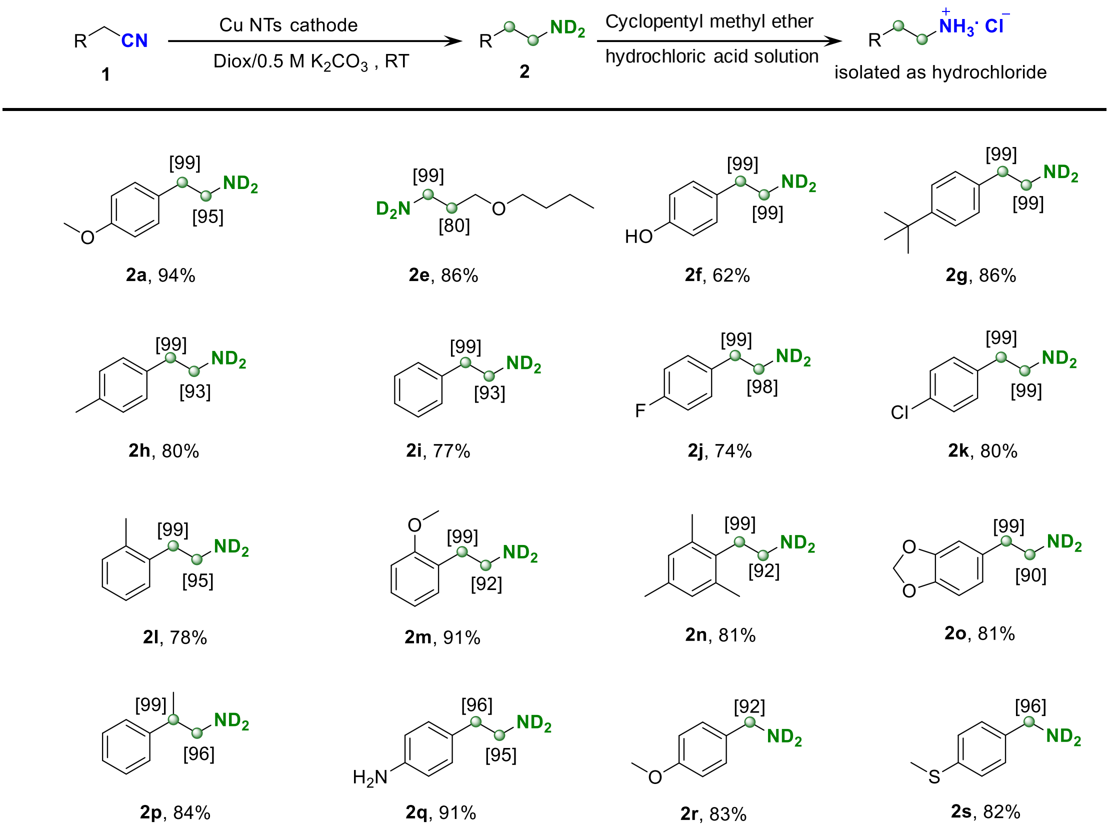

[a]Electrocatalytic deuteration of other functionalized arylacetonitriles using D_2O over the Cu NTs with BTAB under the standard reaction conditions (isolated yields of the hydrochloride of deuterated primary amines are reported in parentheses, and deuterium ratios are presented in brackets).

The NMR spectra and data for the hydrochloride groups of the amine products are provided. When DMSO-d_6 was used as the solvent for the NMR tests, the peaks located at 2.5 ppm and 3.3 ppm in the ^{1}H NMR spectra were assigned to the hydrogen signals of d_6-DMSO and H_2O, respectively. Seven peaks centered at 39.52 ppm in the ^{13}C NMR spectra were assigned to the carbon signals of DMSO-d_6. When $CDCl_3$ was used as the solvent for the NMR tests, the peak at 7.26 ppm in the ^{1}H NMR spectrum is assigned to the hydrogen signal of $CDCl_3$. The three peaks centered at 77 ppm in the ^{13}C NMR spectra were assigned to the carbon signals of $CDCl_3$. Additionally, some carbon atoms are missing in the ^{13}C spectra of the deuterated products due to the presence of D, which is consistent with the reported literature.[18–20]

NMR spectra

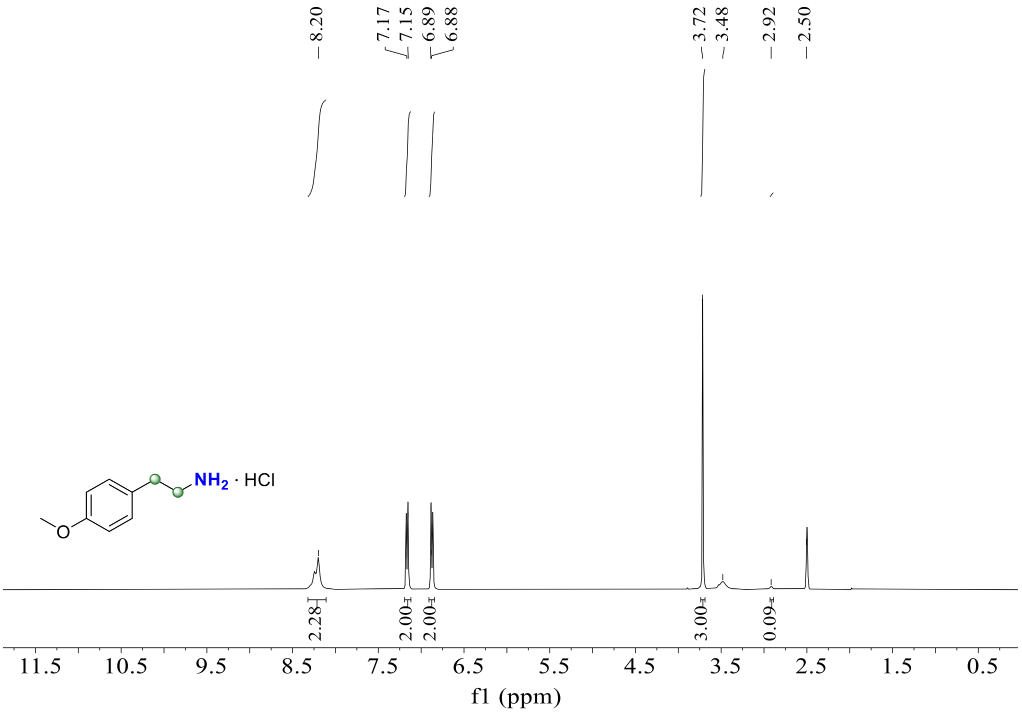

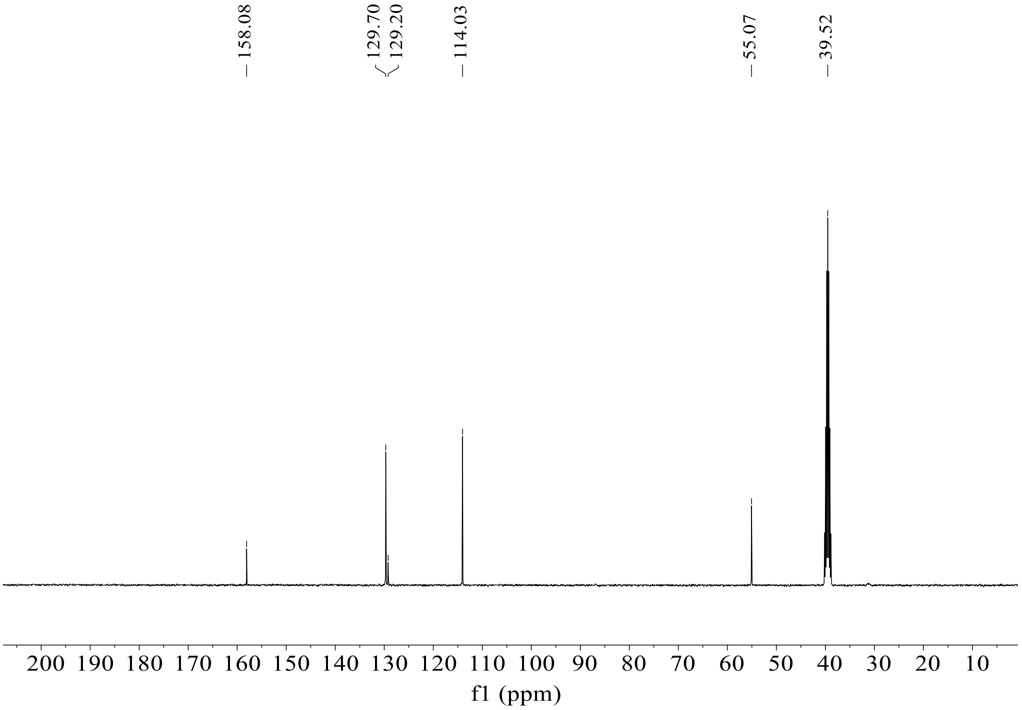

^{1}H NMR (400 MHz, DMSO-d_6) δ [ppm] 8.20 (s, 2.28H), 7.16 (d, J = 8.0 Hz, 2H), 6.88 (d, J = 4.0 Hz, 2H), 3.72 (s, 3H), 2.92 (s, 0.09H); **^{13}C NMR** (101 MHz, DMSO-d_6) δ [ppm] 158.58, 129.70 (2C), 129.20, 114.03 (2C), 55.07.

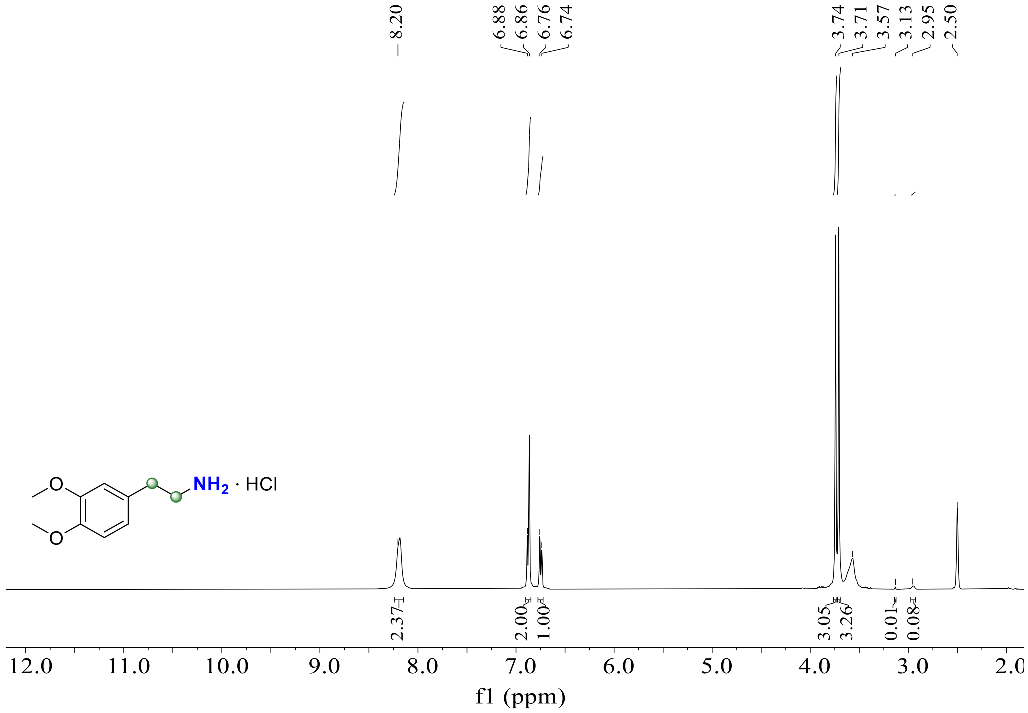

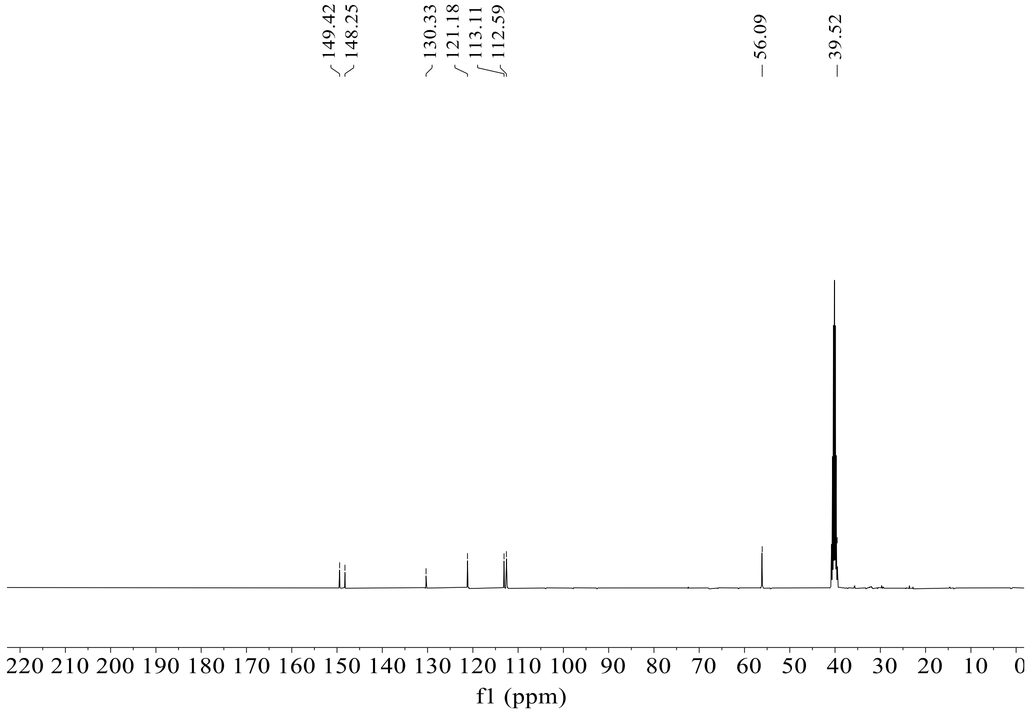

^{1}H NMR (400 MHz, DMSO-d_6) δ [ppm] 8.20 (s, 2.37H), 6.87 (d, J = 8.0 Hz, 2H), 6.75 (d, J = 8.0, 1H), 3.74 (s, 3H), 3.71 (s, 3H), 3.13 (s, 0.01H), 2.95 (s, 0.08H); **^{13}C NMR** (101 MHz, DMSO-d_6) δ [ppm] 149.42, 148.25, 130.33, 121.18, 113.11, 112.59, 56.09 (2C).

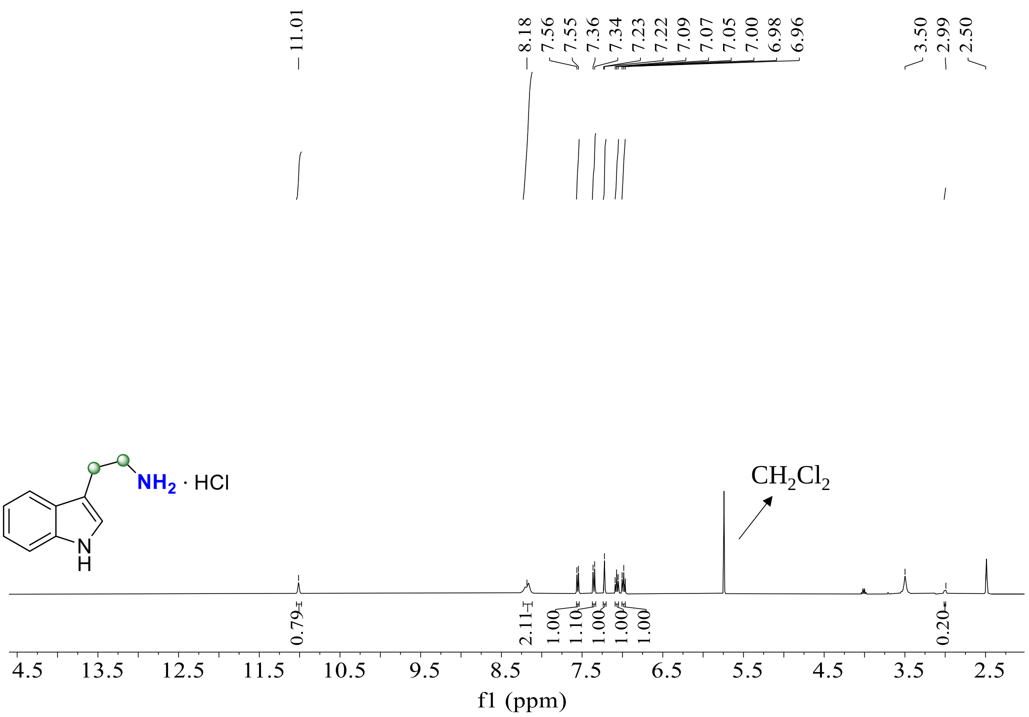

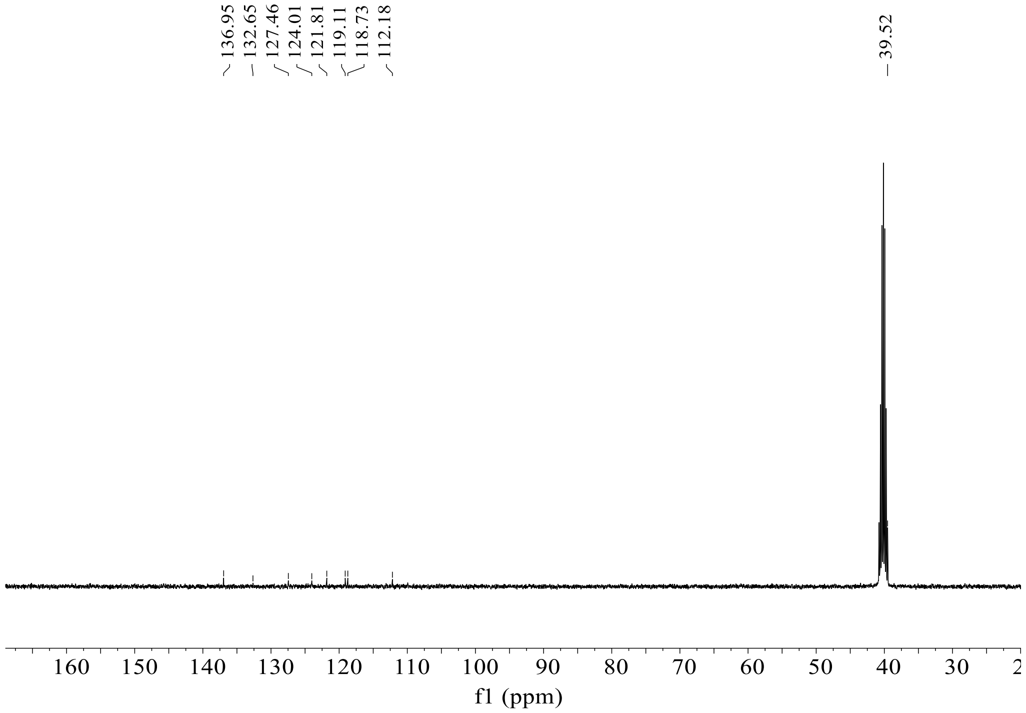

^{1}H NMR (400 MHz, DMSO-d_6) δ [ppm] 11.01 (s, 0.79H), 8.18 (s, 2.11H), 7.55 (d, J = 4.0 Hz, 1H), 7.35 (d, J = 8.0 Hz, 1H), 7.22 (d, J = 4.0 Hz, 1H), 7.07 (t, J = 8.0 Hz, 1H), 6.98 (t, J = 8.0 Hz, 1H), 2.99 (s, 0.20H); **^{13}C NMR** (101 MHz, DMSO-d_6) δ [ppm] 136.95, 132.65, 127.46, 124.01, 121.81, 119.11, 118.73, 112.18.

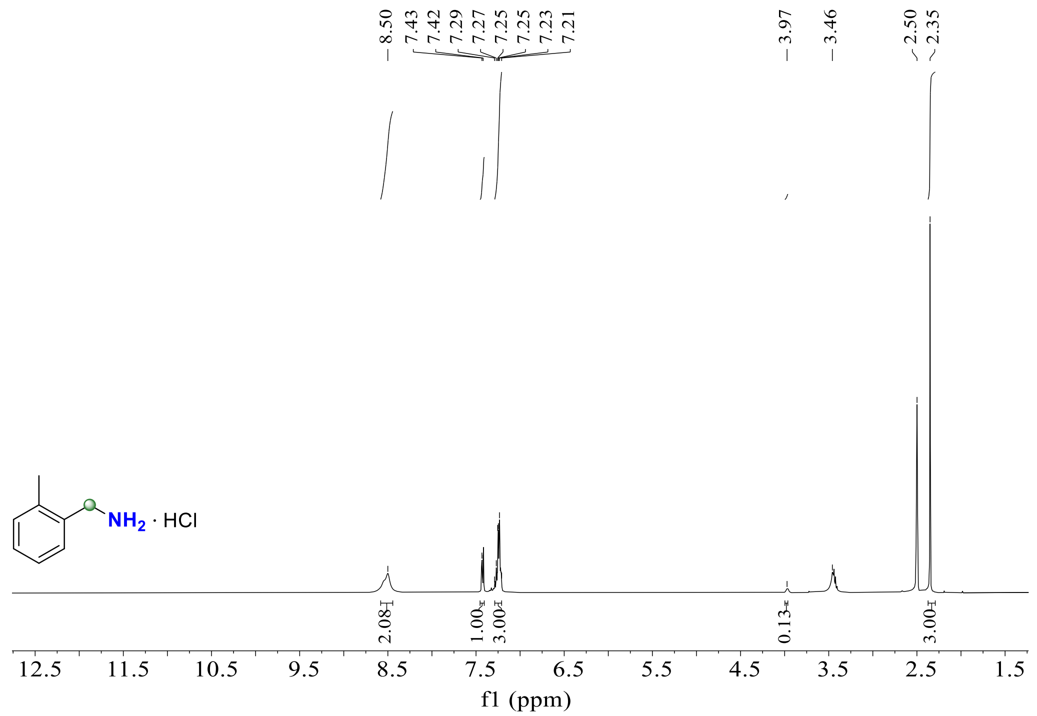

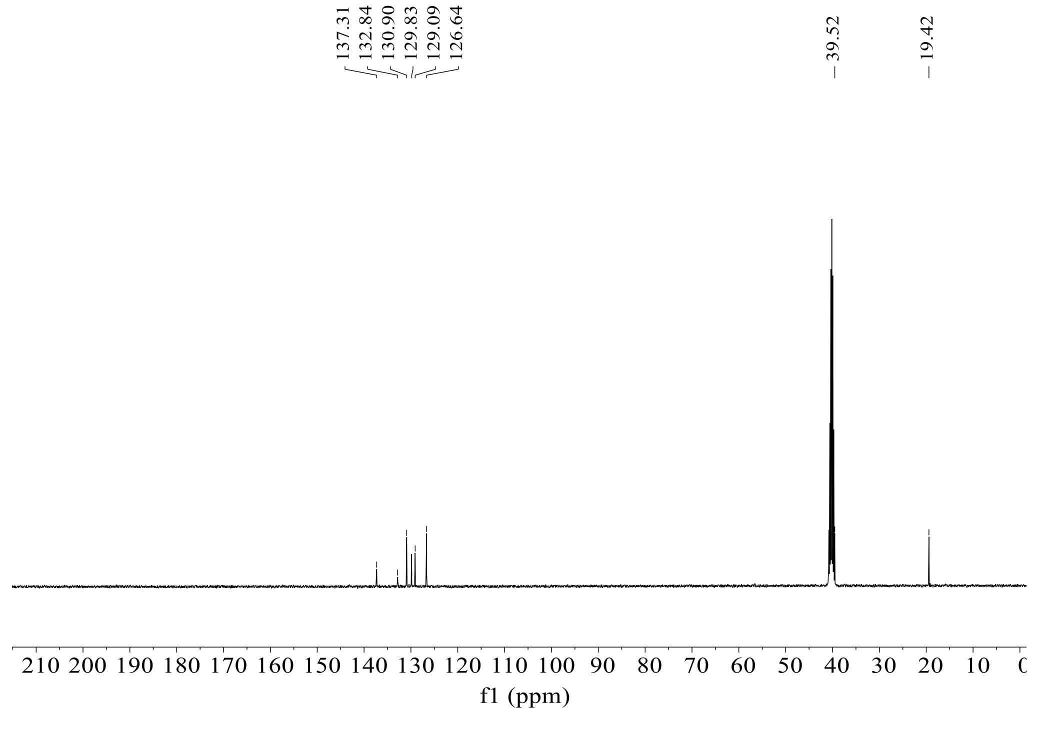

^{1}H NMR (400 MHz, DMSO-d_6) δ [ppm] 8.50 (s, 2.08H), 7.43 (d, J = 4.0 Hz, 1H), 7.29-7.21 (m, 3H), 3.97 (s, 0.13H), 2.35 (s, 3H); **^{13}C NMR** (101 MHz, DMSO-d_6) δ [ppm] 137.31, 132.84, 130.90, 129.83, 129.09, 126.64, 19.42.

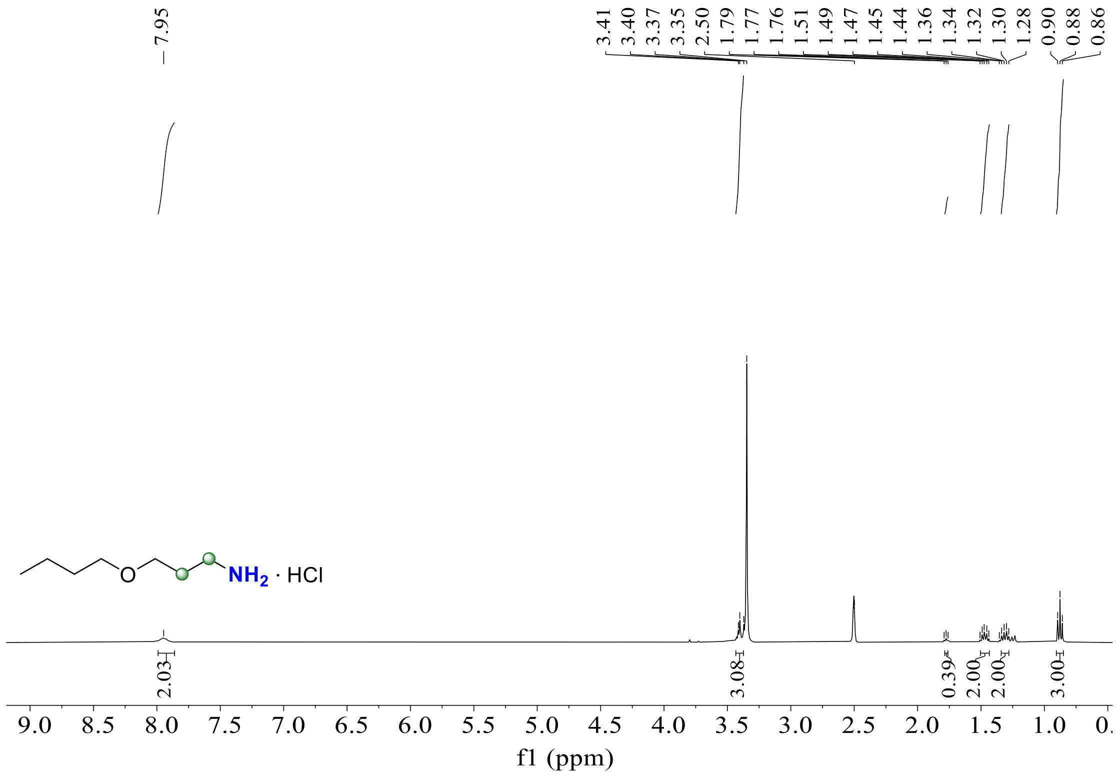

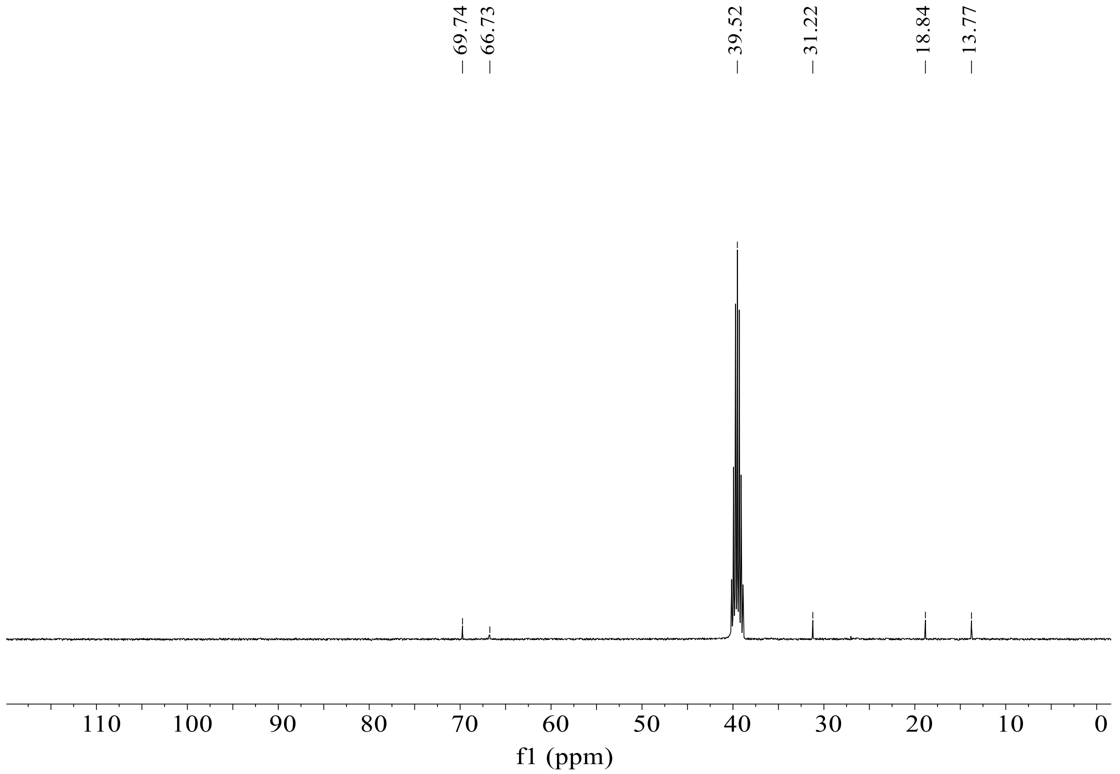

^{1}H NMR (400 MHz, DMSO-d_6) δ [ppm] 7.95 (s, 2.43H), 3.39 (t, $J_1 = 4.0$ Hz, $J_2 = 12.0$ Hz, 3H), 1.77 (t, $J_1 = 8.0$ Hz, $J_2 = 4.0$ Hz 0.39H), 1.51 – 1.45 (m, 2H), 1.35 – 1.28 (m, 2H), 0.87 (t, $J = 8.0$ Hz, 3H); **^{13}C NMR** (101 MHz, DMSO-d_6) δ [ppm] 69.74, 66.73, 31.22, 18.84, 13.77.

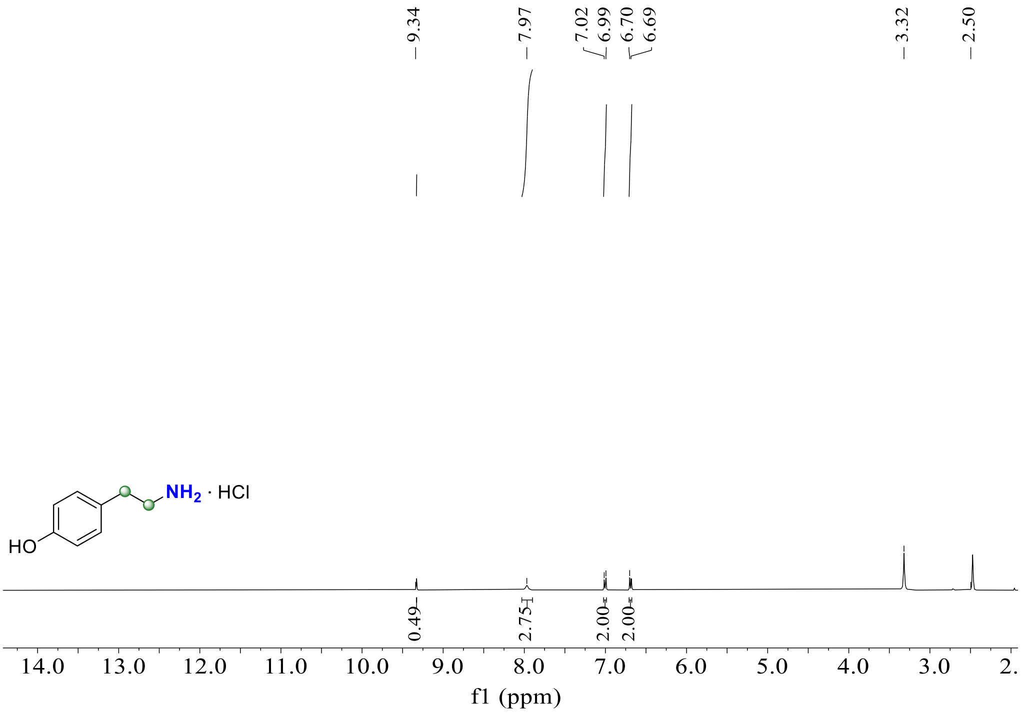

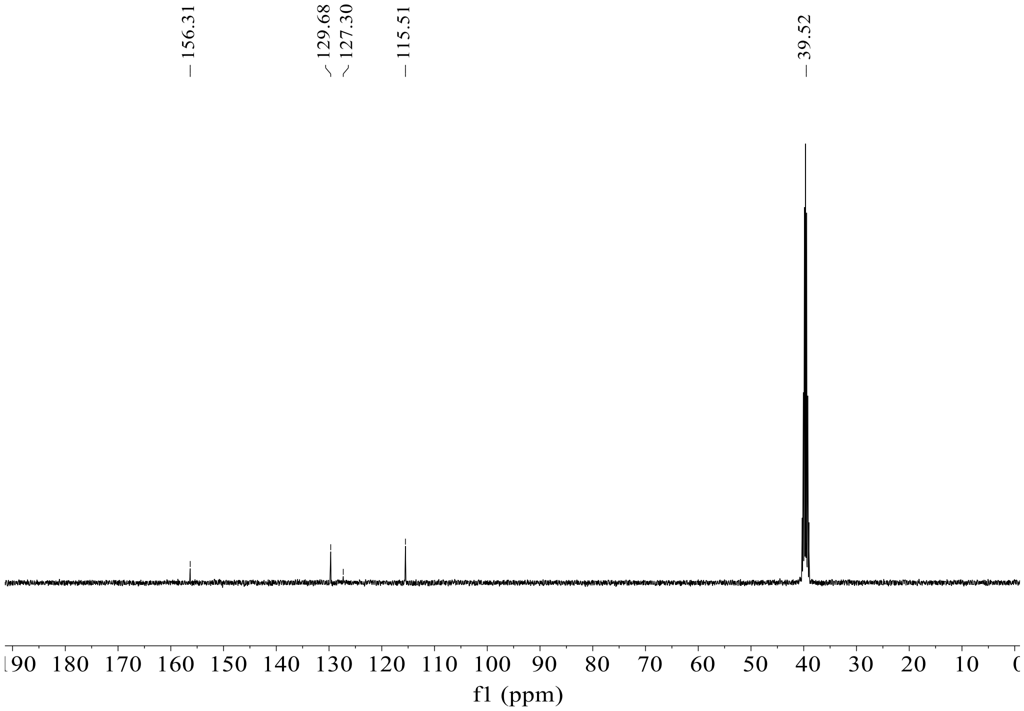

^{1}H NMR (400 MHz, DMSO-d_6) δ [ppm] 9.34 (s, 0.49H), 7.97 (s, 2.75H), 7.01 (d, J = 12.0 Hz, 2H), 6.70 (d, J = 8.0 Hz, 2H); **^{13}C NMR** (101 MHz, DMSO-d_6) δ [ppm] 156.31, 129.68 (2C), 127.30, 115.51 (2C).

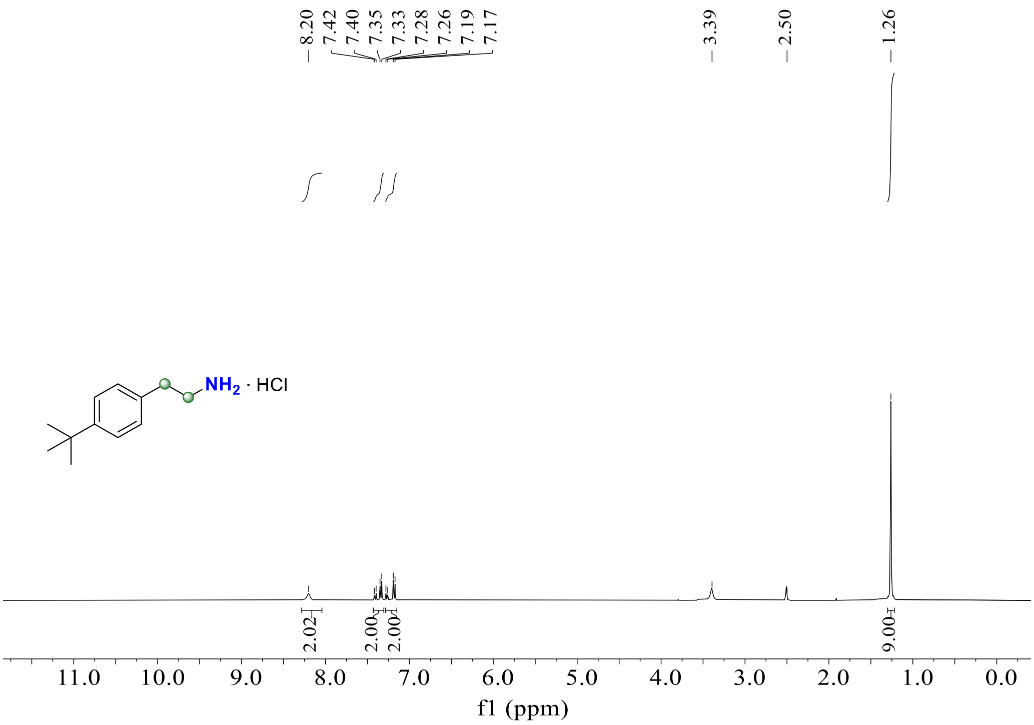

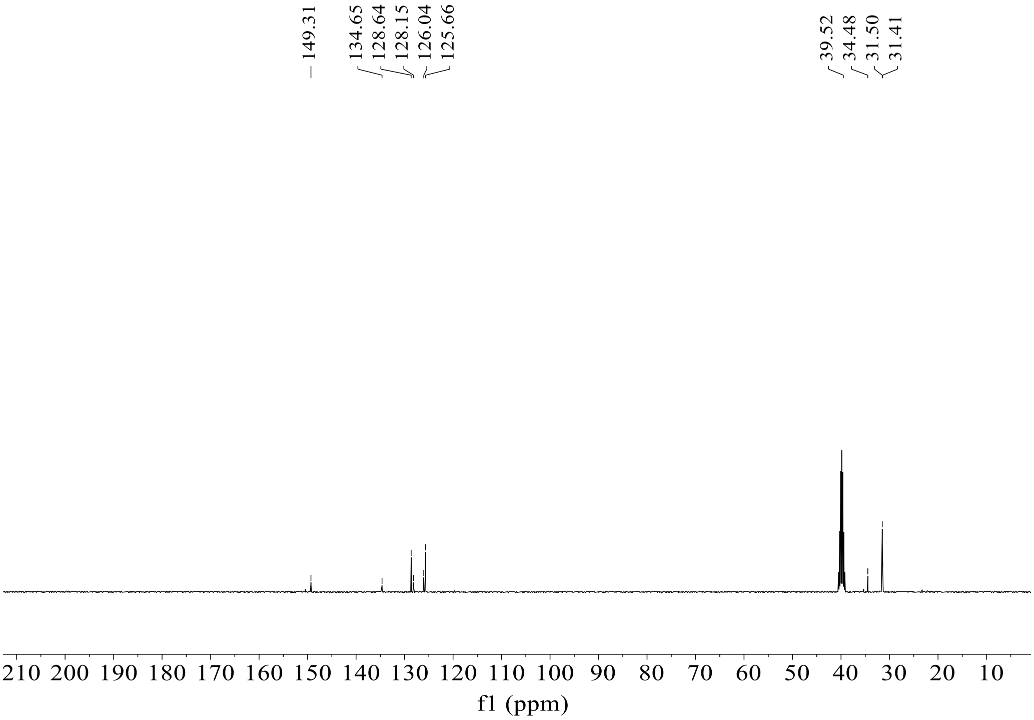

^{1}**H NMR** (400 MHz, DMSO-d_6) δ [ppm] 8.20 (s, 2.20H), 7.38 (dd, J_1 = 28.0, J_2 = 8.0 Hz, 2H), 7.23 (dd, J_1 = 36.0, J_2 = 8.0 Hz, 2H), 1.26 (s, 9H); ^{13}**C NMR** (101 MHz, DMSO-d_6) δ [ppm] 149.31, 134.65, 128.64, 128.15, 126.04, 125.66, 31.48, 31.50 (2C), 31.41.

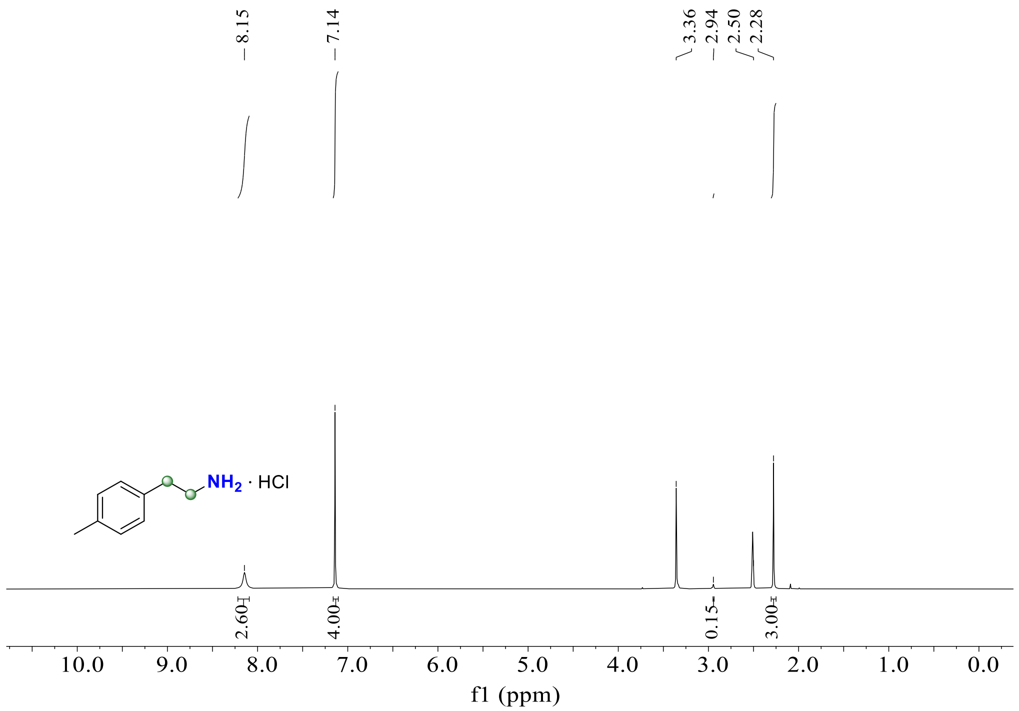

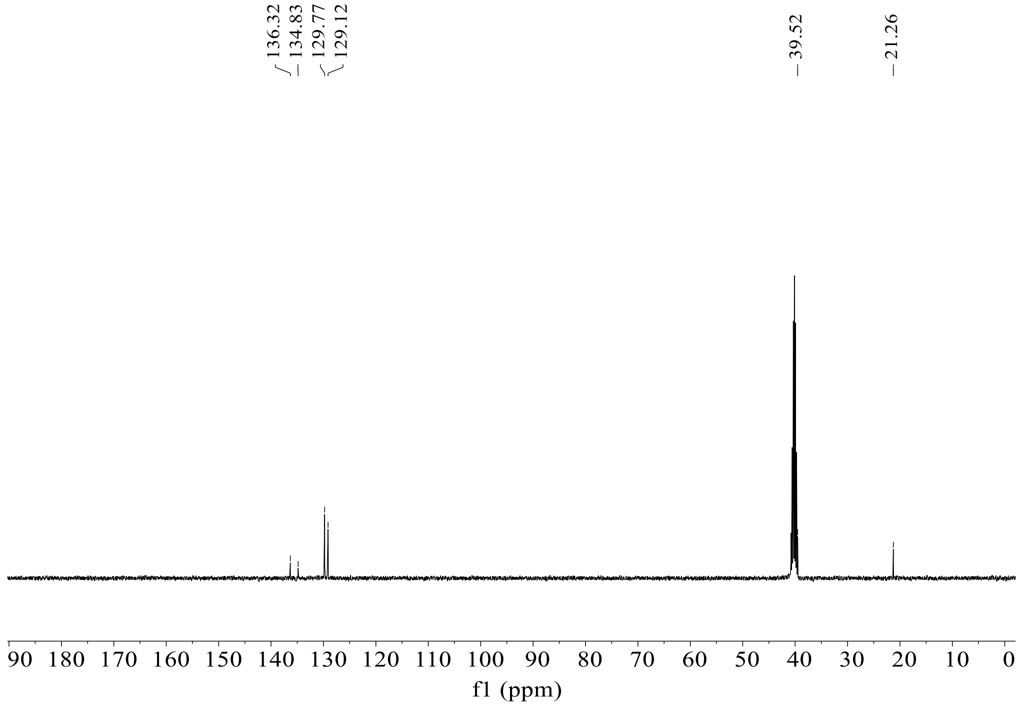

^{1}H NMR (400 MHz, DMSO-d_6) δ [ppm] 8.15 (s, 2.6H), 7.14 (s, 4H), 2.94 (s, 0.15H), 2.28 (s, 3H); **^{13}C NMR** (101 MHz, DMSO-d_6) δ [ppm] 136.32, 134.83, 129.77 (2C), 129.12 (2C), 21.26.

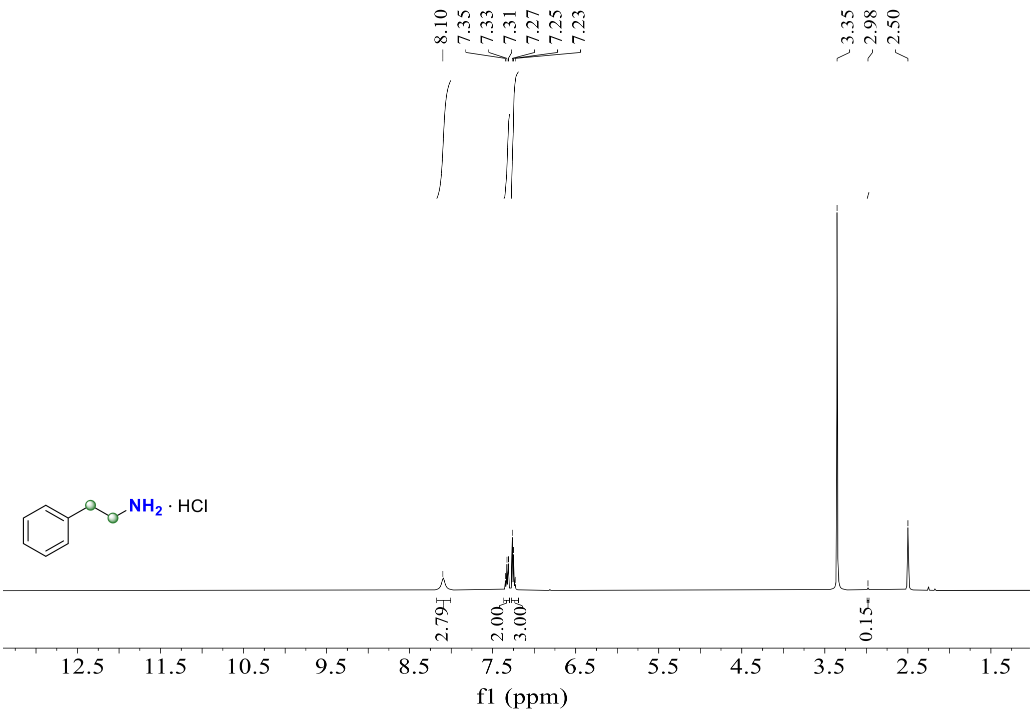

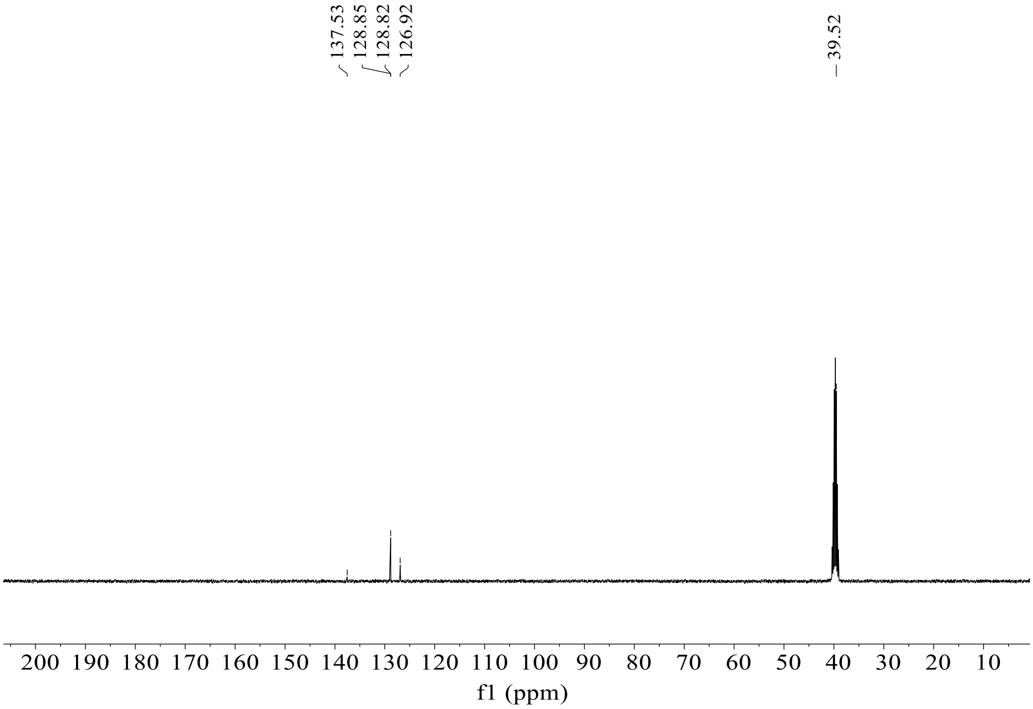

^{1}H NMR (400 MHz, DMSO-d_6) δ [ppm] 8.10 (s, 2.79H), 7.33 (t, J = 8.0 Hz, 2H), 7.25 (t, J = 8.0 Hz, 3H), 2.98 (s, 0.15H); **^{13}C NMR** (101 MHz, DMSO-d_6) δ [ppm] 137.53, 128.85 (2C), 128.82 (2C), 126.92.

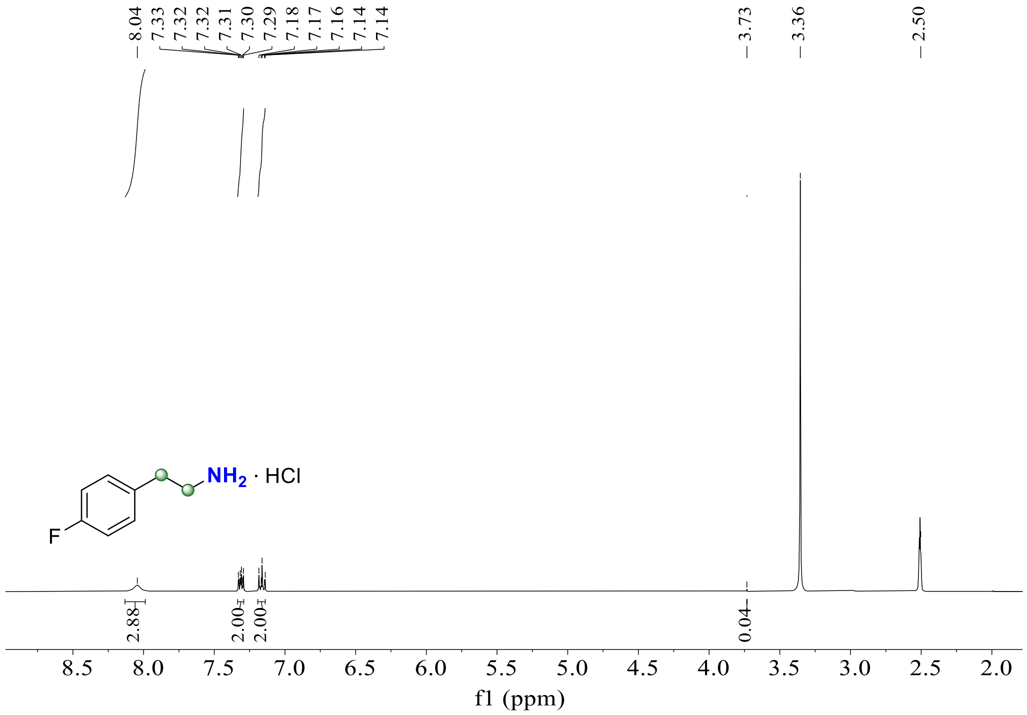

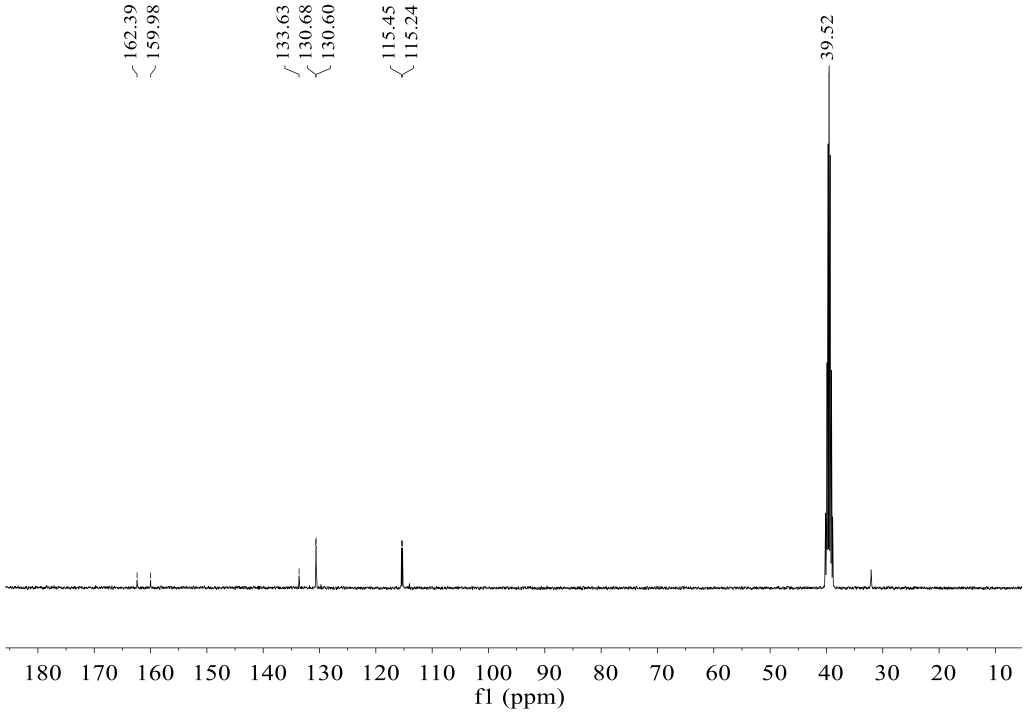

^{1}H NMR (400 MHz, DMSO-d_6) δ [ppm] 8.04 (s, 2.88H), 7.33–7.29 (m, 2H), 7.18–7.14 (m, 2H), 3.73 (s, 0.04H); **^{13}C NMR** (101 MHz, DMSO-d_6) δ [ppm] 161.19 (d, J = 243.41 Hz), 133.63, 130.64 (d, J = 8.08 Hz, 2C), 115.35 (d, J = 21.21 Hz, 2C).

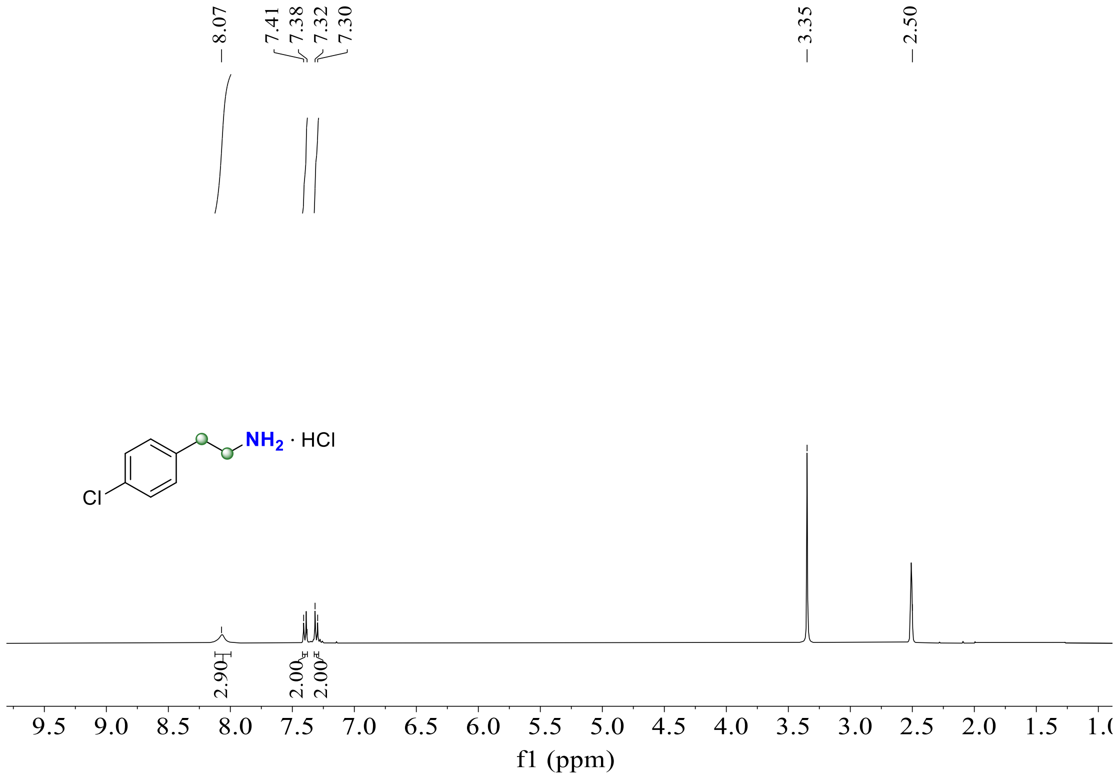

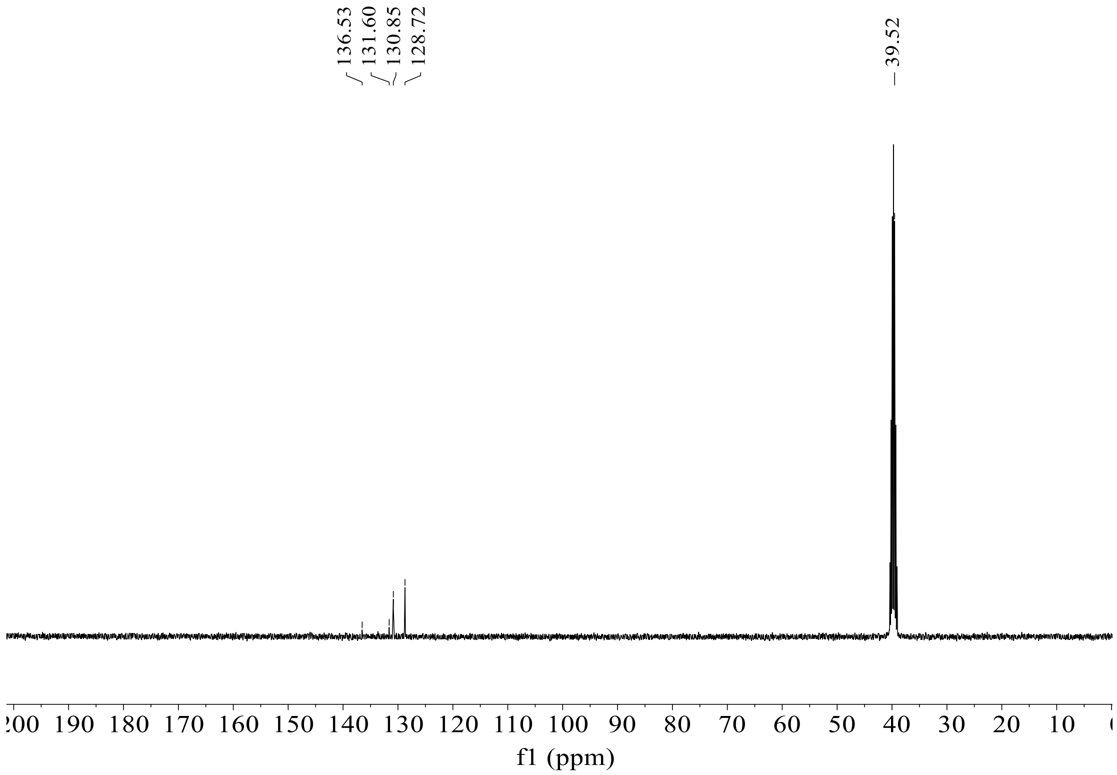

^{1}H NMR (400 MHz, DMSO-d_6) δ [ppm] 8.07 (s, 2.90H), 7.39 (d, J = 12.0 Hz, 2H), 7.31 (d, J = 8.0 Hz, 2H); **^{13}C NMR** (101 MHz, DMSO-d_6) δ [ppm] 136.53, 131.60, 130.85 (2C), 128.72 (2C).

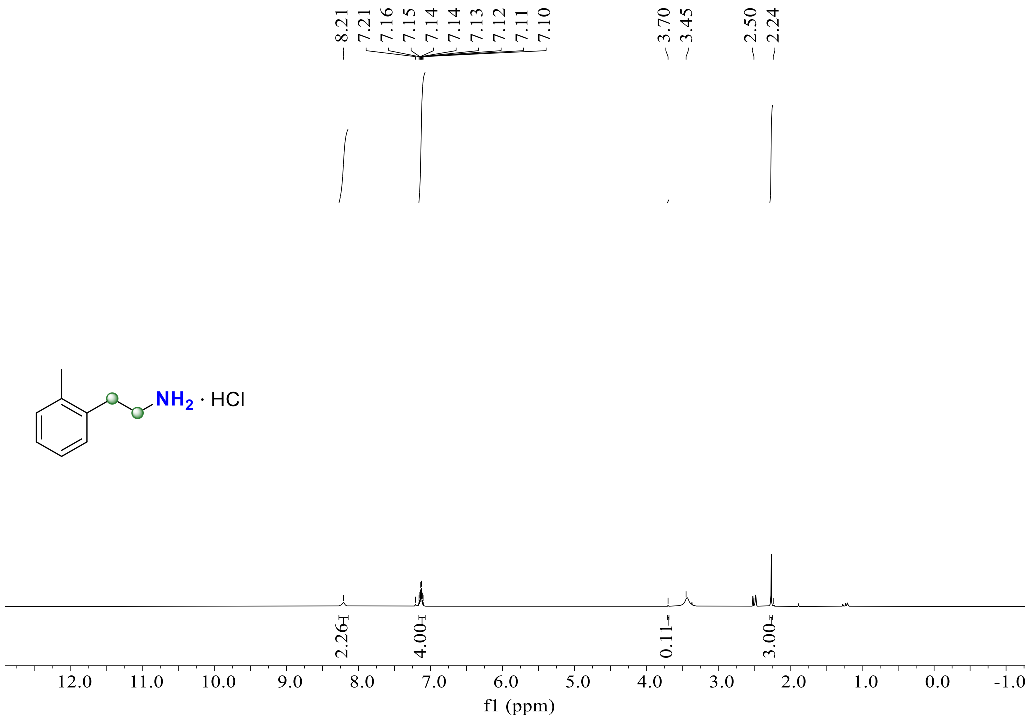

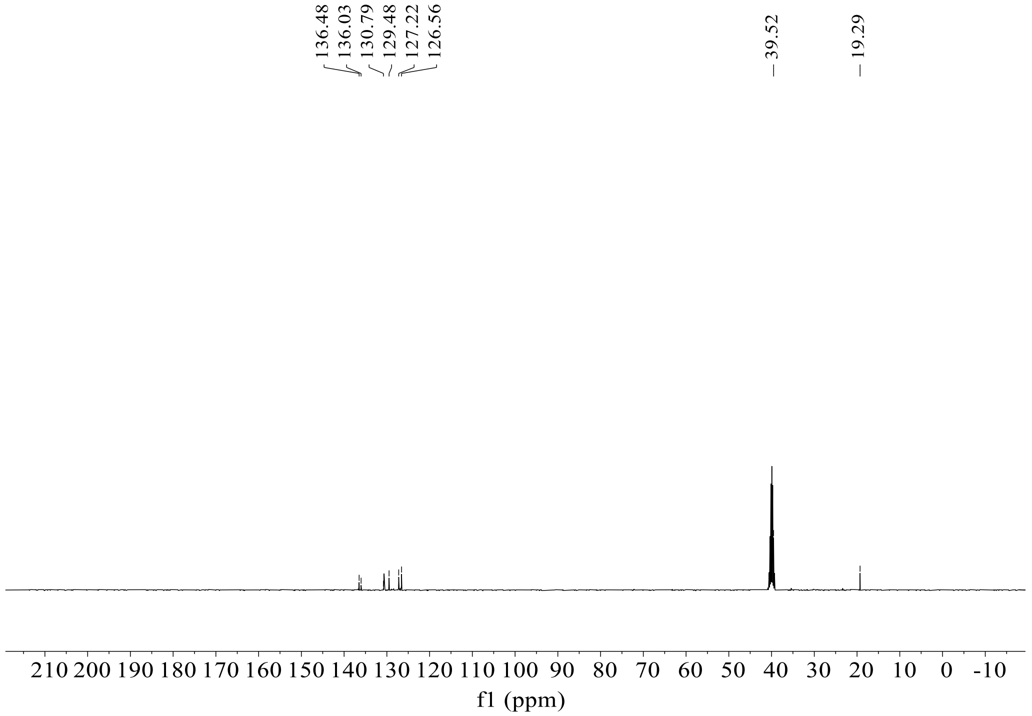

^{1}H NMR (400 MHz, DMSO-d_6) δ [ppm] 8.21 (s, 2.26H), 7.21–7.10 (m, 4H), 3.70 (s, 0.11H), 2.24 (s, 3H); **^{13}C NMR** (101 MHz, DMSO-d_6) δ [ppm] 136.48, 136.03, 130.79, 129.48, 127.22, 126.56, 19.29.

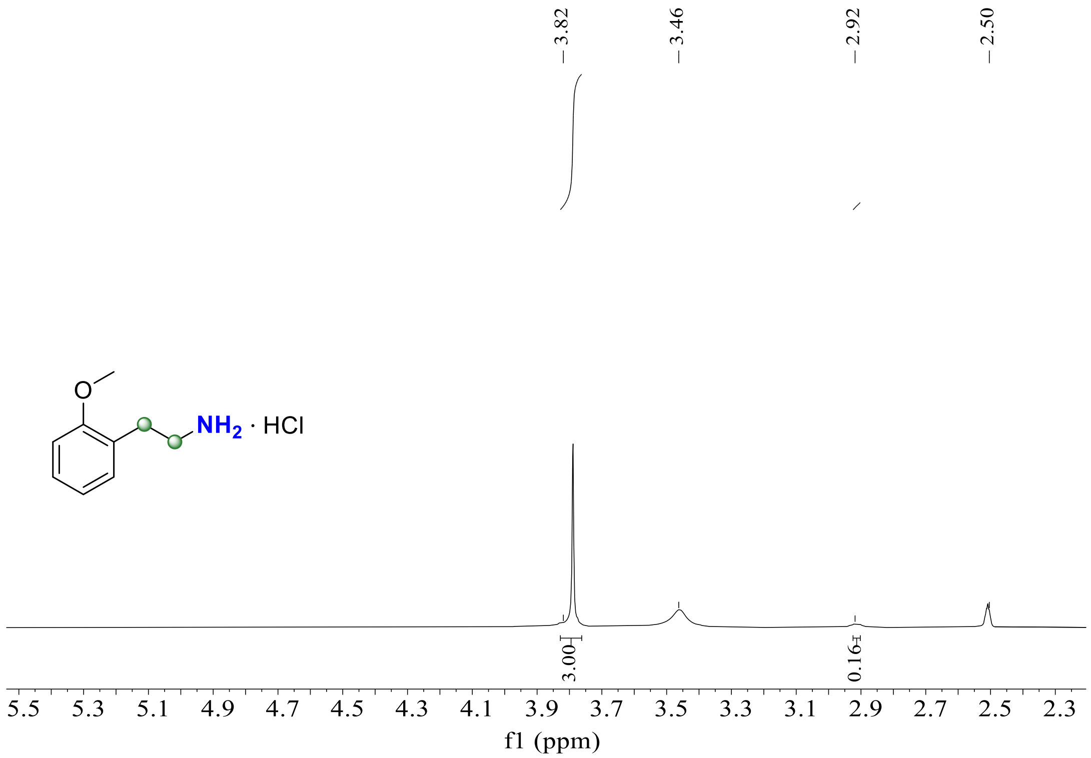

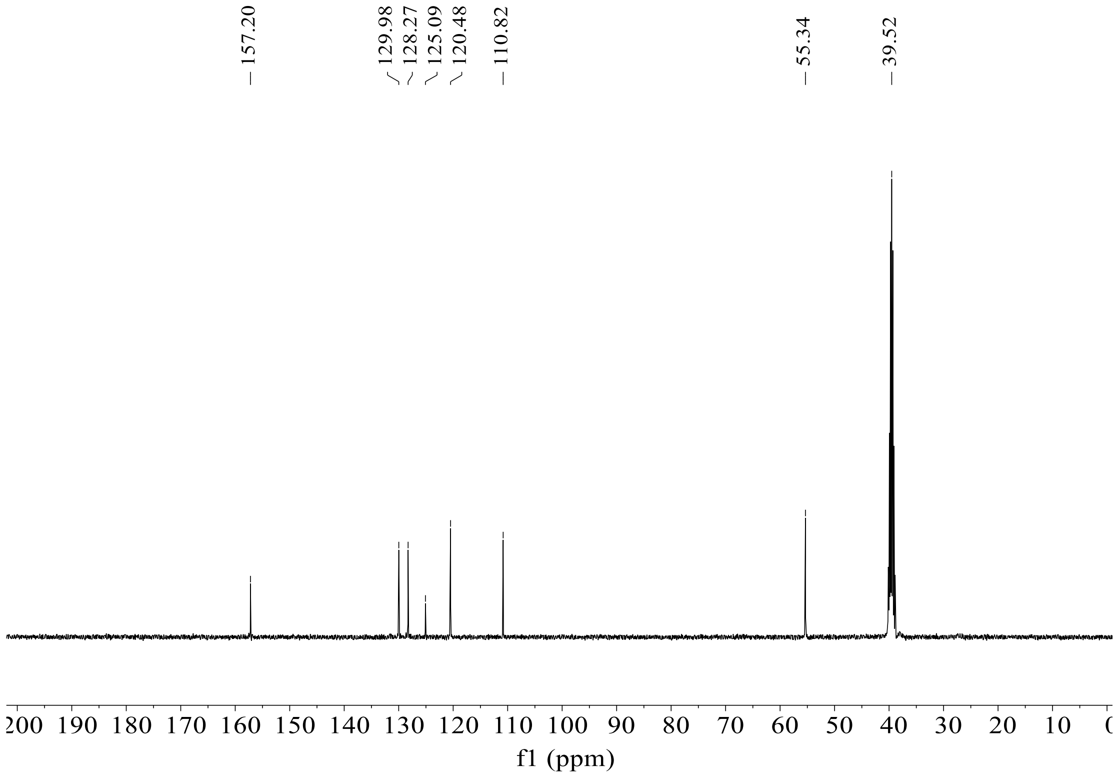

^{1}**H NMR** (400 MHz, DMSO-d_6) δ [ppm] 8.24 (s, 2.77H), 7.24 (t, J = 8.0 Hz, 1H), 7.17 (d, J = 4.0 Hz, 1H), 6.98 (d, J = 8.0 Hz, 2H), 6.90 (t, J_1 = 4.0 Hz, J_2 = 8.0 Hz, 1H), 3.82 (s, 3H), 2.93 (s, 0.16H); ^{13}**C NMR** (101 MHz, DMSO-d_6) δ [ppm] 157.20, 129.98, 128.27, 125.09, 120.48, 110.82, 55.34.

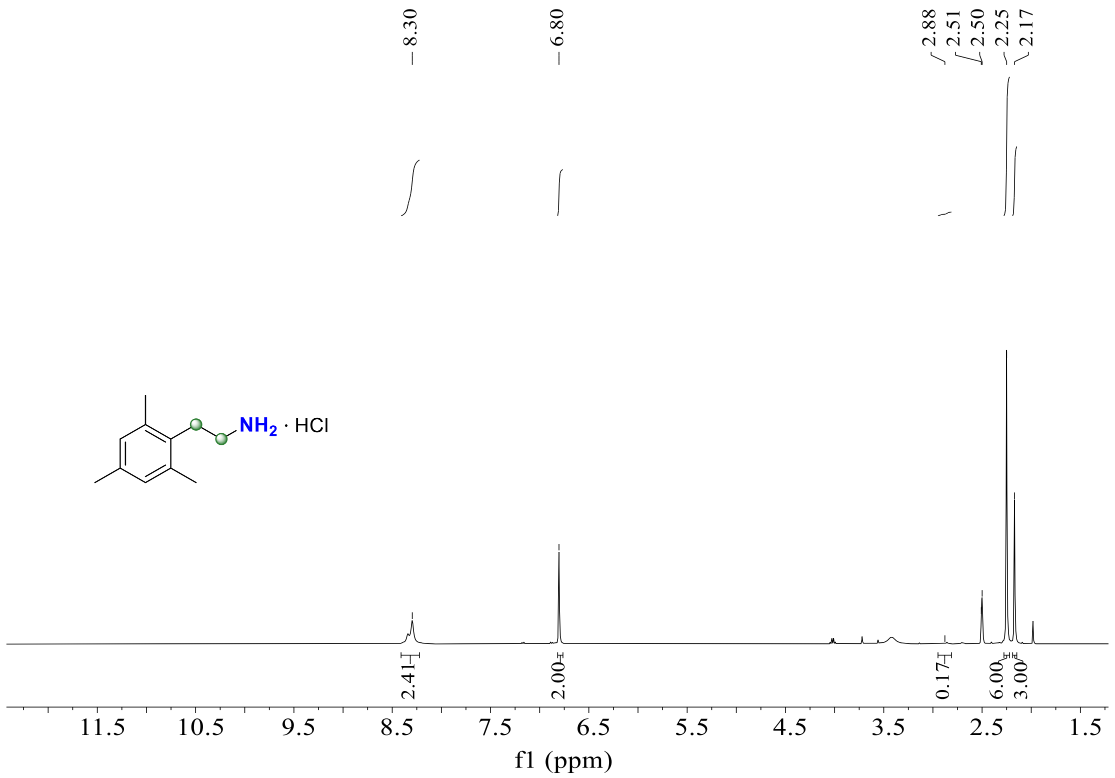

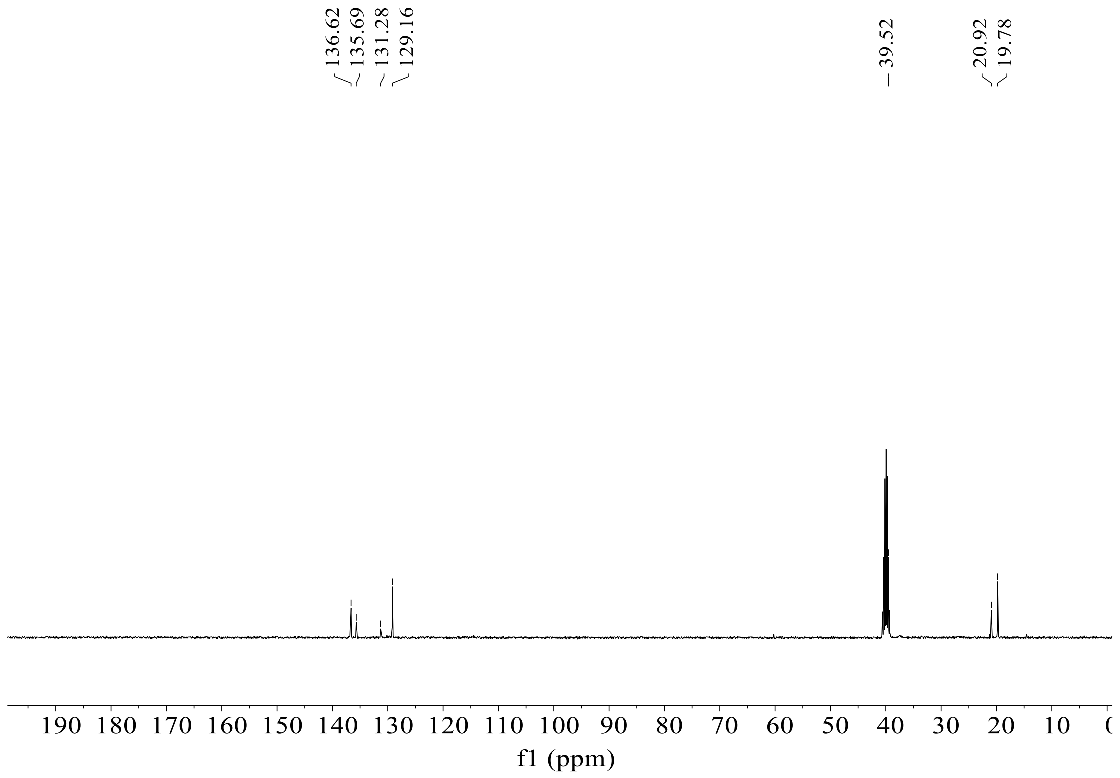

^{1}H NMR (400 MHz, DMSO-*d*$_6$) δ [ppm] 8.30 (s, 2.41H), 6.80 (s, 2H), 2.88 (s, 0.17H), 2.25 (s, 6H), 2.17 (s, 3H); **^{13}C NMR** (101 MHz, DMSO-*d*$_6$) δ [ppm] 136.62 (2C), 135.69, 131.28, 129.16 (2C), 20.92, 19.78 (2C).

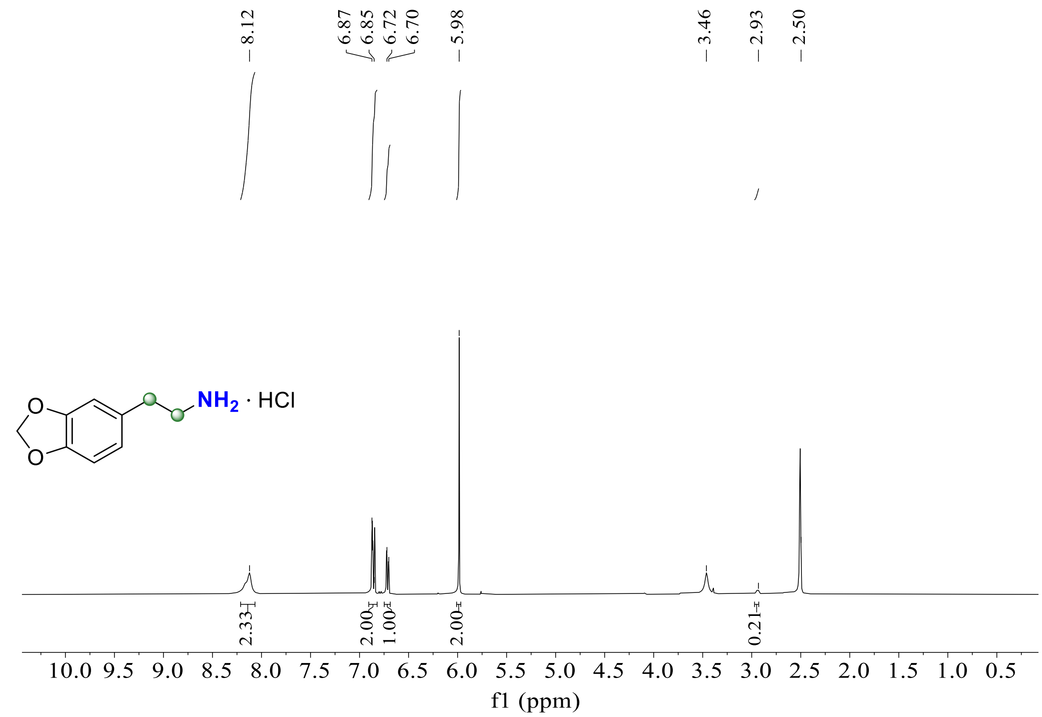

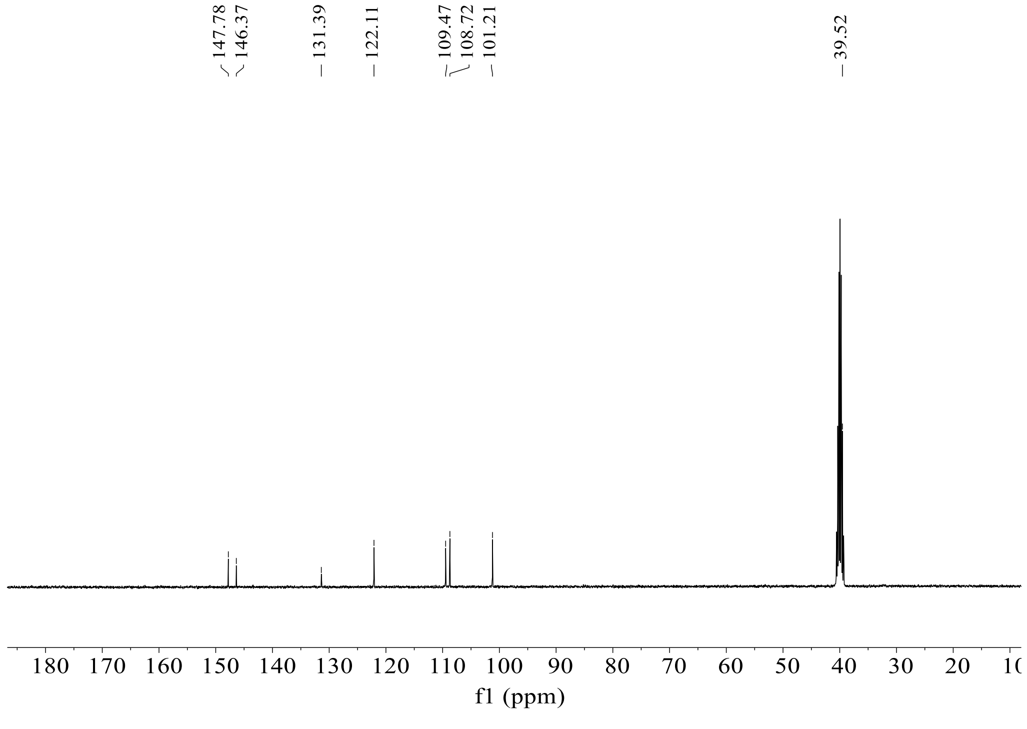

^{1}H NMR (400 MHz, DMSO-d_6) δ [ppm] 8.12 (s, 2.33H), 6.86 (d, J = 8.0 Hz, 2H), 6.71 (d, J = 8.0 Hz, 1H), 5.98 (s, 2H), 2.93 (s, 0.21H); **^{13}C NMR** (101 MHz, DMSO-d_6) δ [ppm] 147.78, 146.37, 131.39, 122.11, 109.47, 108.72, 101.21.

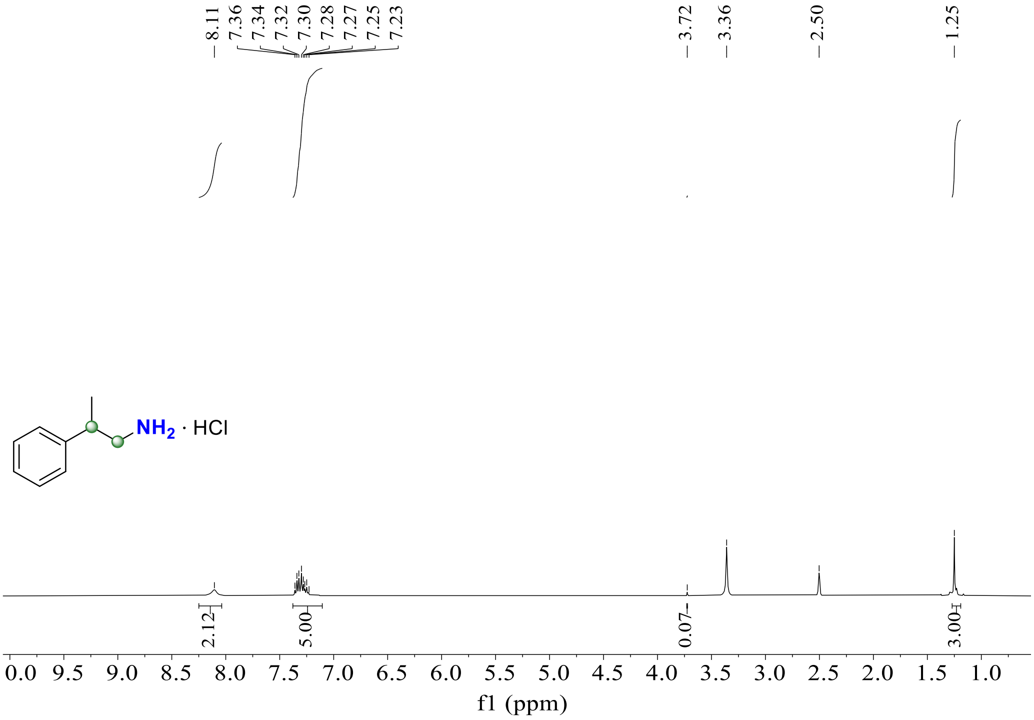

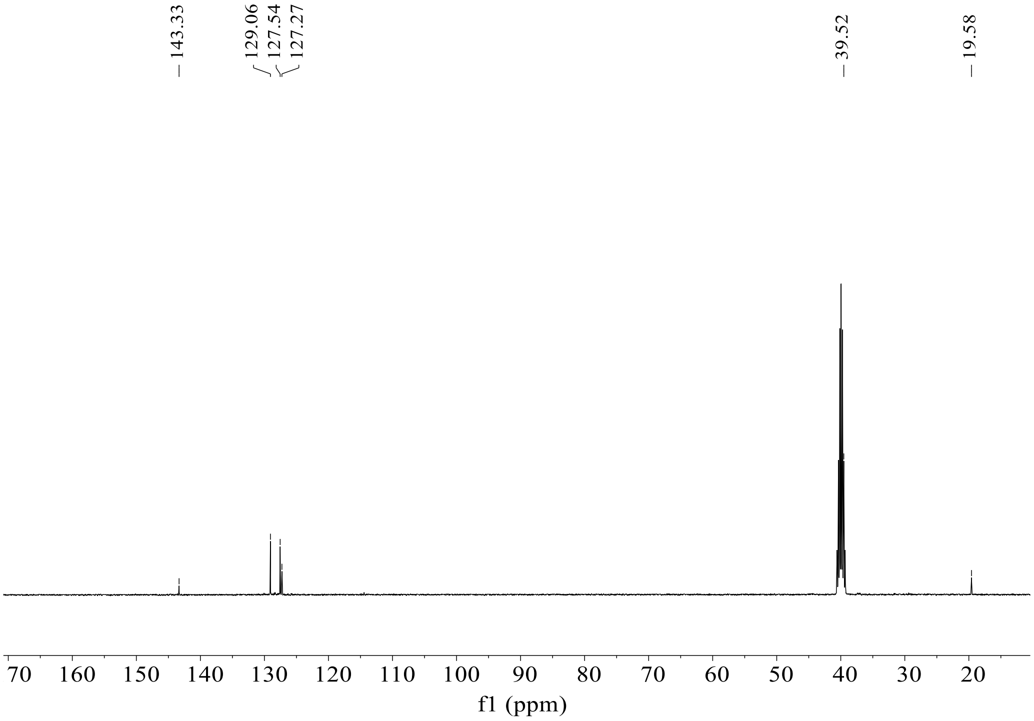

^{1}H NMR (400 MHz, CD_3CN-d_3) δ [ppm] 8.11 (s, 2.12H), 7.36 – 7.23 (m, 5H), 3.72 (s, 0.07H), 1.25 (s, 3H); **^{13}C NMR** (101 MHz, CD_3CN-d_3) δ [ppm] 143.33, 129.06 (2C), 127.54 (2C), 127.27, 19.58.

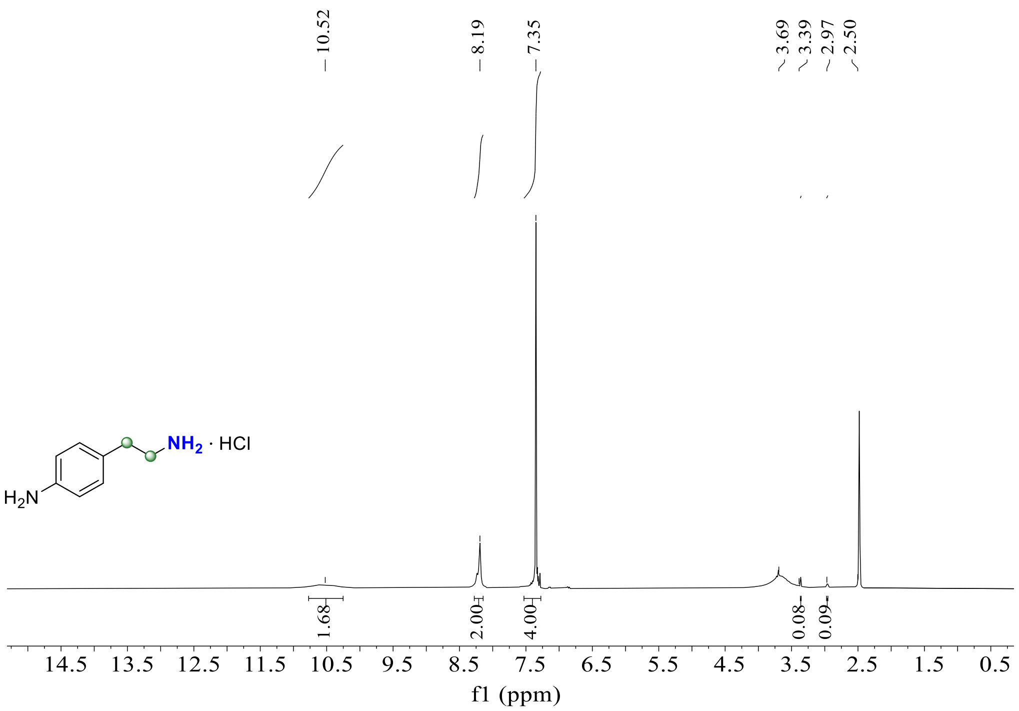

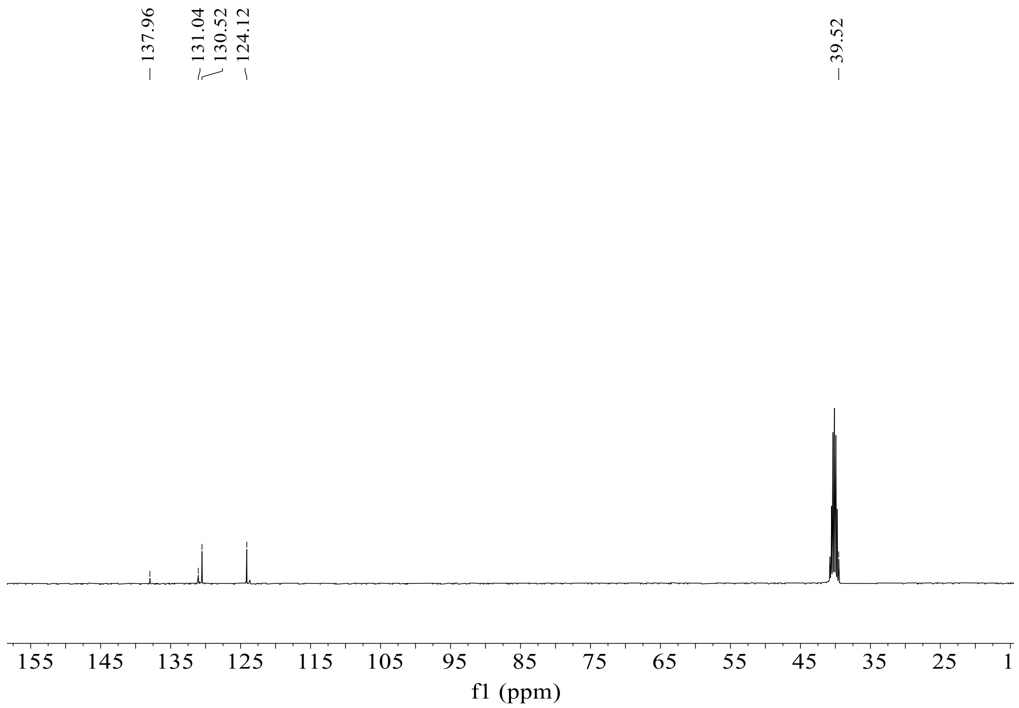

^{1}H NMR (400 MHz, DMSO-d_6) δ [ppm] 10.52 (s, 1.68H), 8.19 (s, 2H), 7.44 – 7.29 (m, 4H), 3.39 (s, 0.08H), 2.97 (s, 0.09H); **^{13}C NMR** (101 MHz, DMSO-d_6) δ [ppm] 137.96, 131.04, 130.52 (2C), 124.12 (2C).

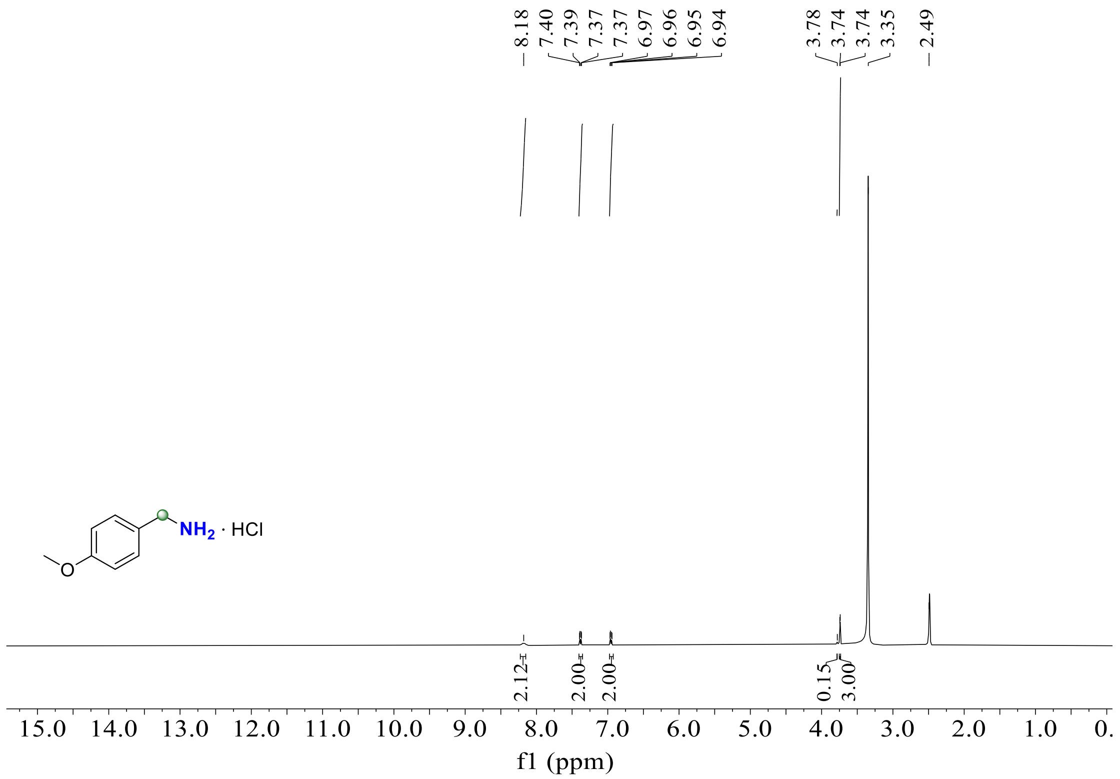

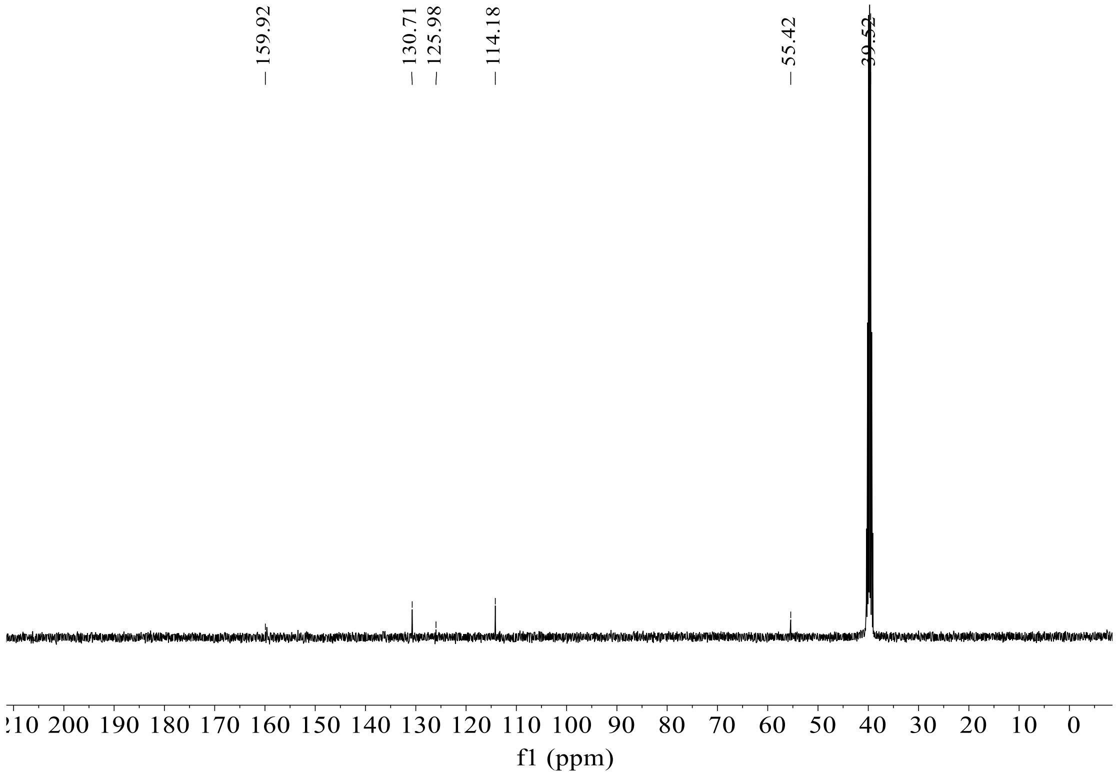

^{1}H NMR (400 MHz, DMSO-d_6) δ [ppm] 8.19 (s, 2.12H), 7.41–7.38 (m, 2H), 6.98–6.95 (s, 2H), 3.79 (s, 0.15H), 3.75 (s, 3H); **^{13}C NMR** (101 MHz, DMSO-d_6) δ [ppm] 159.92, 130.71 (2C), 125.98, 114.18 (2C), 55.42.

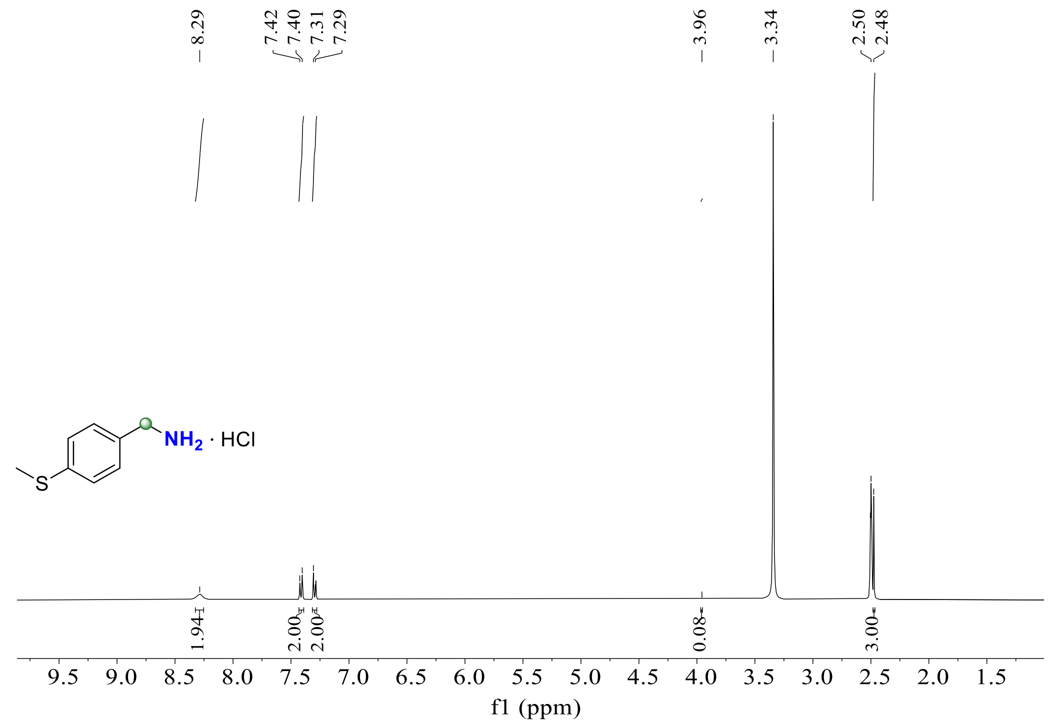

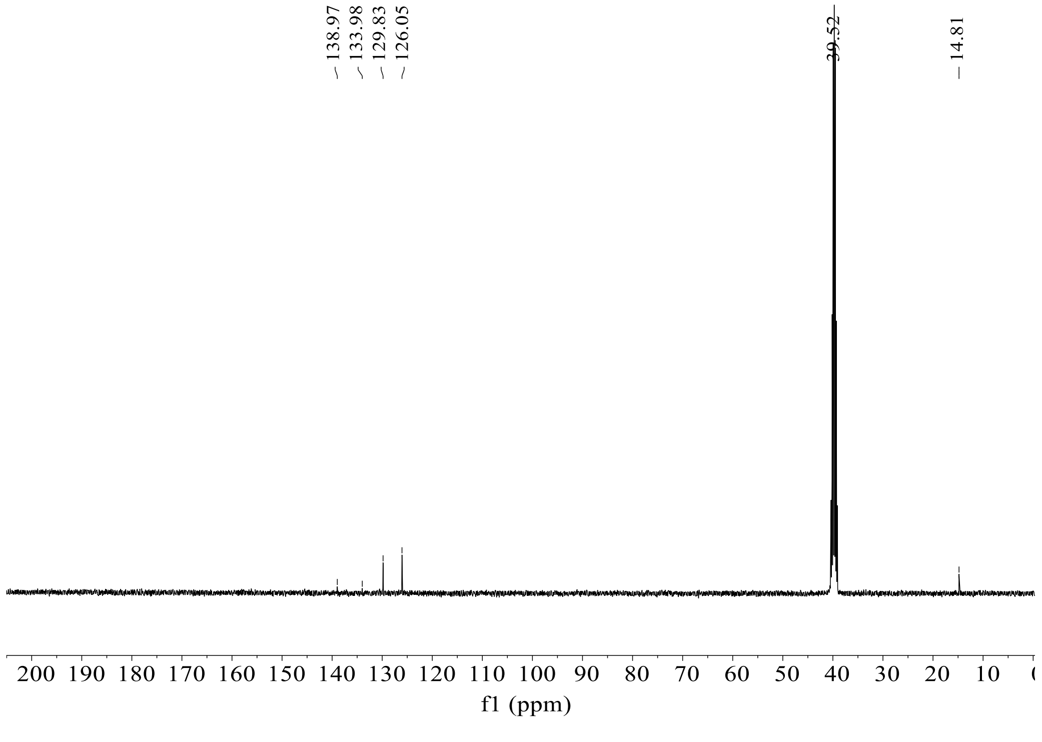

^{1}H NMR (400 MHz, DMSO-d_6) δ [ppm] 8.29 (s, 1.94H), 7.41 (d, J = 8.0 Hz, 2H), 7.30 (d, J = 8.0 Hz, 2H), 3.96 (s, 0.08H), 2.48 (s, 3H); **^{13}C NMR** (101 MHz, DMSO-d_6) δ [ppm] 138.97, 133.98, 129.83 (2C), 126.05 (2C), 14.81.

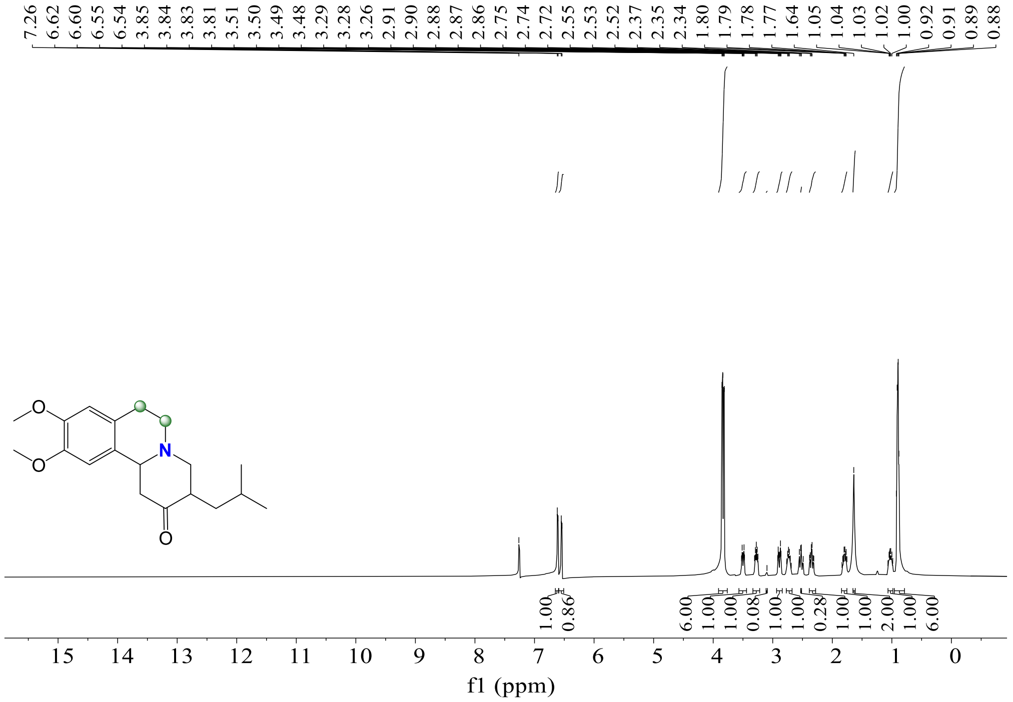

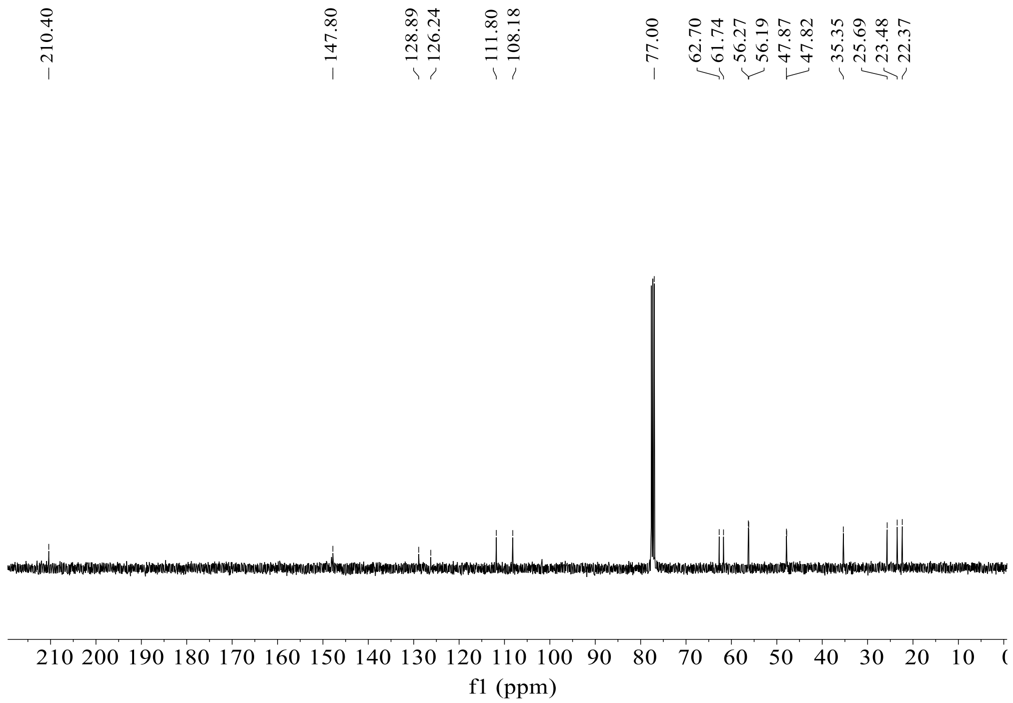

^{1}H NMR (400 MHz, $CDCl_3$) δ [ppm] 6.61 (d, J = 8.0 Hz, 1H), 6.55 (d, J = 4.0 Hz, 1H), 3.85 – 3.81 (m, 6H), 3.51 – 3.48, (m, 1H), 3.29 – 3.26 (m, 1H), 3.10 (s, 0.08H), 2.91 – 2.86 (m, 1H), 2.75 – 2.72 (m, 1H), 2.55 – 2.52 (m, 0.28H), 2.37 – 2.34 (m, 1H), 1.80 – 1.77 (m, 1H), 1.64 (s, 2H), 1.05 – 1.00 (m, 1H), 0.92 – 0.88 (m, 1H); **^{13}C NMR** (101 MHz, $CDCl_3$) δ [ppm] 210.40, 147.80, 128.89, 126.24, 111.80, 108.18, 62.70, 61.74, 56.27, 56.19, 47.87, 47.82, 35.35, 25.69, 23.48, 22.37.

References

1. Fu, K., Han, D., Ma, C. & Bohn, P. W. Ion selective redox cycling in zero-dimensional nanopore electrode arrays at low ionic strength. *Nanoscale* **9**, 5164–5171 (2017).
2. Gao, F. Y. et al. High-curvature transition-metal chalcogenide nanostructures with a pronounced proximity effect enable fast and selective CO_2 electroreduction. *Angew. Chem. Int. Ed.* **59**, 8706–8712 (2020).
3. Xiong, G. et al. Bioinspired leaves-on-branchlet hybrid carbon nanostructure for supercapacitors. *Nat. Commun.* **9**, 790 (2018).
4. Nairan, A. et al. Proton selective adsorption on Pt–Ni nano-thorn array electrodes for superior hydrogen evolution activity. *Energy Environ. Sci.* **14**, 1594–1601 (2021).
5. Lee, S. H. & Rasaiah, J. C. Proton transfer and the mobilities of the H^+ and OH^- ions from studies of a dissociating model for water. *J. Chem. Phys.* **135**, 124505 (2011).
6. Pulagara, N.V., Kaur, G., and Lahiri, I. Enhanced field emission performance of growth-optimized CuO nanorods. Appl. Phys. A *127* (2021).
7. Zhang, D. et al. Highly efficient electrochemical hydrogenation of acetonitrile to ethylamine for primary amine synthesis and promising hydrogen storage. *Chem Catal.* **1**, 393–406 (2021).
8. Andreiadis, E. S. et al. Molecular Engineering of a Cobalt-Based Electrocatalytic Nanomaterial for H_2 Evolution under Fully Aqueous Conditions. *Nat. Chem.* **5**, 48–53 (2013).
9. Kresse, G. & Furthmüller, J. Efficient iterative schemes for *ab initio* total-energy calculations using a plane-wave basis set. *Phys. Rev. B* **54**, 11169–11186 (1996).
10. Blochl, P. E. Projector augmented-wave method. *Phys. Rev., B Condens. Matter* **50**, 17953–17979 (1994).
11. Perdew, J. P., Burke, K. & Ernzerhof, M. Generalized gradient approximation made simple. *Phys. Rev. Lett.* **77**, 3865–3868 (1996).
12. Mathew, K., Sundararaman, R., Letchworth-Weaver, K., Arias, T. A. & Hennig, R. G. Implicit solvation model for density-functional study of nanocrystal surfaces and reaction pathways. *J. Chem. Phys.* **140**, 084106 (2014).
13. Xia, G.; Benmohamed, R.; Morimoto, R. I.; Kirsch, D. R.; Silverman, R. B. Deuteration and Fluorination of 1,3-Bis(2-Phenylethyl)Pyrimidine-2,4,6(1h,3h,5h)-Trione to Improve Its Pharmacokinetic Properties. *Bioorg. Med.*

Chem. Lett. **24**, 5098–5101 (2014).

14. Tsukinoki, T. et al. Preparation of deuteriated benzylamines and phenethylamine with raney alloys in an alkaline deuterium oxide solution. *J. Label. Comp. Radiopharm.* **34**, 839–844 (1994).
15. Mándity, I. M.; Martinek, T. A.; Darvas, F.; Fülöp, F. A Simple, Efficient, and Selective Deuteration Via a Flow Chemistry Approach. *Tetrahedron Lett.* **50**, 4372–4374 (2009).
16. Sumii, Y. et al. 3-(phenethylamino)demethyl(oxy)aaptamine as an anti-dormant mycobacterial substance: Isolation, evaluation and total synthesis. *Tetrahedron Lett.* **61**, 151924 (2020).
17. Yu, Q. S. et al. Preparation and characterization of tetrabenazine enantiomers against vesicular monoamine transporter 2. *ACS Medicinal Chem. Lett.* **1**, 105–109 (2010).
18. Wang, X. et al. General and practical potassium methoxide/disilane-mediated dehalogenative deuteration of (hetero)arylhalides. *J. Am. Chem. Soc.* **140**, 10970–10974 (2018).
19. Yu, R. P., Hesk, D., Rivera, N., Pelczer, I. & Chirik, P. J. Iron-catalysed tritiation of pharmaceuticals. *Nature* **529**, 195-199 (2016).
20. Loh, Y. Y. et al. Photoredox-catalyzed deuteration and tritiation of pharmaceutical compounds. *Science* **358**, 1182–1187 (2017).
