# Supplementary material for: Microenvironment regulation breaks the Faradaic efficiency-current density trade-off for electrocatalytic deuteration using D2O
Source: Nat Commun. 2024 Jun 19;15:5231. doi: 10.1038/s41467-024-49544-y (PMC11187139; doi:10.1038/s41467-024-49544-y)
Supplement: Supplementary file 3 — Description of Additional Supplementary Files [file 41467_2024_49544_MOESM3_ESM.pdf]

Description of Additional Supplementary Files

Supplementary Data 1: The optimized DFT computational models. Atomic coordinates of Cu model without and with surfactant in AIMD and DFT processes.
