# Supplementary material for: Microenvironment regulation breaks the Faradaic efficiency-current density trade-off for electrocatalytic deuteration using D2O
Source: Nat Commun. 2024 Jun 19;15:5231. doi: 10.1038/s41467-024-49544-y (PMC11187139; doi:10.1038/s41467-024-49544-y)
Supplement: Supplementary file 4 — Supplementary Data 1 [file 41467_2024_49544_MOESM4_ESM.pdf]

AIMD initial state of without surfactant

```
   1.000000000000000
    10.2424999999999997    0.0000000000000000    0.0000000000000000
     0.0000000000000000    8.8702000000000005    0.0000000000000000
     0.0000000000000000    0.0000000000000000   30.6609000000000016
   Cu   H    O    C    N
    64  103   49    8    1
Direct
  0.50271000  0.50257000  0.27251000
  0.12884000  0.75026000  0.27216000
  0.62297000  0.25196000  0.27204000
  0.87849000  0.75594000  0.27146000
  0.75273000  0.99964000  0.27134000
  0.50035000  0.00067000  0.27118000
  0.87270000  0.25755000  0.27113000
  0.74794000  0.50470000  0.27074000
  0.62741000  0.75097000  0.26935000
  0.12215000  0.25488000  0.26921000
  0.37175000  0.25581000  0.26902000
  0.25621000  0.50399000  0.26849000
  0.99712000  0.50462000  0.26828000
  0.25116000  0.00202000  0.26791000
  0.37495000  0.75298000  0.26786000
  0.00222000  0.00387000  0.26739000
  0.74821000  0.33581000  0.20407000
  0.49861000  0.34178000  0.20260000
  0.87110000  0.59014000  0.20239000
  0.24822000  0.83275000  0.20229000
  0.99922000  0.83831000  0.20224000
  0.99926000  0.33748000  0.20213000
  0.62378000  0.08277000  0.20172000
  0.75293000  0.83883000  0.20147000
  0.49938000  0.83539000  0.20147000
  0.12503000  0.58490000  0.20132000
  0.62700000  0.58772000  0.20127000
  0.12627000  0.08241000  0.20038000
  0.37983000  0.58732000  0.20036000
  0.24766000  0.33559000  0.20011000
  0.87397000  0.08675000  0.19998000
  0.37350000  0.08615000  0.19854000
  0.86605000  0.41938000  0.13612000
  0.37307000  0.91231000  0.13506000
  0.99921000  0.66466000  0.13456000
  0.75160000  0.16471000  0.13437000
```

0.37992000	0.41581000	0.13433000
0.62532000	0.92546000	0.13403000
0.24743000	0.65937000	0.13397000
0.11780000	0.42376000	0.13392000
0.99833000	0.17032000	0.13392000
0.75263000	0.66996000	0.13380000
0.12652000	0.91323000	0.13337000
0.50058000	0.66588000	0.13326000
0.62934000	0.41809000	0.13304000
0.25040000	0.16690000	0.13270000
0.87556000	0.91919000	0.13248000
0.50231000	0.17180000	0.13111000
0.25000000	0.00000000	0.06523000
0.62500000	0.75000000	0.06523000
0.87500000	0.25000000	0.06523000
0.00000000	0.50000000	0.06523000
0.87500000	0.75000000	0.06523000
0.12500000	0.75000000	0.06523000
0.37500000	0.25000000	0.06523000
0.62500000	0.25000000	0.06523000
0.75000000	0.50000000	0.06523000
0.12500000	0.25000000	0.06523000
0.25000000	0.50000000	0.06523000
0.50000000	0.00000000	0.06523000
0.00000000	0.00000000	0.06523000
0.75000000	0.00000000	0.06523000
0.50000000	0.50000000	0.06523000
0.37500000	0.75000000	0.06523000
0.14188000	0.66203000	0.98494000
0.74391000	0.91020000	0.98480000
0.72407000	0.29325000	0.98289000
0.09434000	0.03267000	0.98145000
0.67236000	0.13193000	0.97940000
0.49241000	0.59235000	0.97914000
0.09045000	0.81660000	0.97593000
0.40312000	0.03290000	0.96417000
0.89997000	0.37927000	0.95518000
0.82818000	0.52188000	0.95376000
0.47851000	0.73522000	0.95145000
0.53162000	0.95582000	0.94994000
0.79669000	0.91037000	0.93690000
0.10842000	0.05892000	0.93327000
0.67230000	0.61867000	0.92703000
0.25510000	0.47151000	0.90705000

0.55874000	0.19544000	0.90466000
0.76841000	0.56841000	0.89023000
0.08353000	0.30937000	0.88375000
0.95285000	0.19413000	0.87879000
0.29675000	0.34746000	0.87582000
0.13336000	0.98090000	0.87014000
0.37763000	0.07835000	0.86184000
0.58004000	0.16380000	0.85543000
0.86237000	0.42695000	0.85529000
0.80494000	0.77013000	0.85118000
0.00787000	0.90096000	0.84848000
0.72776000	0.42539000	0.82546000
0.33023000	0.96152000	0.82495000
0.75734000	0.90745000	0.82272000
0.55877000	0.97248000	0.79332000
0.05960000	0.71620000	0.79228000
0.27144000	0.71253000	0.79208000
0.66392000	0.08964000	0.79122000
0.06069000	0.33666000	0.79071000
0.79880000	0.10498000	0.76270000
0.55881000	0.31828000	0.76255000
0.46118000	0.66844000	0.76255000
0.62136000	0.49755000	0.76251000
0.39441000	0.43665000	0.76233000
0.01220000	0.59936000	0.75539000
0.22404000	0.59570000	0.75520000
0.01329000	0.21982000	0.75383000
0.81404000	0.20183000	0.72111000
0.57405000	0.41512000	0.72096000
0.47641000	0.76528000	0.72096000
0.63660000	0.59440000	0.72092000
0.40964000	0.53349000	0.72074000
0.47738000	0.08260000	0.68212000
0.23683000	0.08562000	0.66079000
0.69404000	0.54267000	0.66052000
0.59491000	0.83204000	0.65834000
0.98928000	0.19703000	0.64809000
0.60920000	0.41557000	0.64340000
0.51007000	0.70494000	0.64122000
0.84437000	0.83760000	0.63791000
0.49970000	0.03672000	0.63214000
0.75953000	0.71050000	0.62079000
0.00586000	0.71704000	0.61740000
0.09748000	0.99527000	0.59410000

0.04035000	0.57295000	0.58976000
0.75670000	0.70452000	0.58809000
0.59986000	0.16615000	0.58217000
0.46645000	0.68292000	0.56348000
0.01882000	0.87284000	0.56335000
0.49543000	0.32632000	0.55773000
0.64144000	0.54405000	0.55083000
0.44612000	0.13793000	0.54915000
0.75364000	0.74315000	0.54473000
0.35939000	0.79514000	0.54210000
0.30098000	0.32246000	0.53717000
0.22875000	0.63146000	0.52755000
0.55920000	0.09852000	0.52551000
0.19428000	0.46717000	0.52396000
0.68686000	0.40531000	0.52374000
0.07654000	0.32959000	0.52248000
0.32820000	0.04744000	0.52221000
0.22858000	0.92889000	0.52099000
0.53619000	0.95595000	0.49826000
0.88735000	0.91606000	0.49322000
0.94369000	0.75940000	0.49195000
0.56560000	0.49059000	0.47770000
0.66264000	0.30682000	0.47701000
0.57090000	0.77449000	0.46907000
0.62585000	0.57463000	0.43616000
0.72289000	0.39085000	0.43547000
0.57781000	0.89498000	0.43407000
0.29889000	0.65570000	0.42576000
0.38406000	0.06628000	0.42563000
0.19615000	0.33428000	0.42285000
0.78503000	0.90571000	0.39908000
0.34140000	0.53040000	0.38716000
0.23866000	0.20897000	0.38426000
0.44946000	0.13859000	0.38257000
0.79330000	0.45177000	0.37404000
0.62930000	0.28232000	0.36615000
0.15248000	0.65352000	0.36444000
0.05764000	0.42401000	0.36165000
0.75108000	0.88444000	0.35176000
0.64298000	0.12448000	0.35115000
0.04625000	0.77254000	0.35059000
0.66193000	0.51050000	0.34889000
0.95142000	0.54303000	0.34780000
0.64793000	0.23002000	0.99451000

0.06460000	0.72195000	0.99073000
0.84638000	0.43698000	0.97330000
0.74808000	0.96826000	0.95806000
0.14119000	0.99147000	0.95731000
0.49510000	0.62676000	0.94900000
0.43687000	0.94580000	0.94696000
0.76285000	0.64451000	0.91740000
0.21585000	0.40667000	0.88421000
0.97972000	0.30873000	0.87881000
0.51867000	0.20638000	0.87558000
0.03991000	0.97822000	0.87097000
0.30415000	0.03839000	0.84560000
0.78743000	0.49110000	0.84225000
0.83579000	0.85140000	0.83224000
0.62763000	0.01297000	0.81108000
0.98612000	0.67624000	0.77605000
0.19796000	0.67258000	0.77585000
0.98720000	0.29670000	0.77448000
0.76620000	0.20341000	0.74850000
0.52621000	0.41671000	0.74836000
0.42858000	0.76687000	0.74836000
0.58876000	0.59598000	0.74832000
0.36181000	0.53507000	0.74814000
0.65208000	0.45207000	0.66967000
0.55295000	0.74144000	0.66749000
0.54598000	0.03756000	0.66137000
0.80241000	0.74701000	0.64706000
0.41516000	0.19644000	0.60963000
0.96590000	0.65219000	0.59716000
0.37329000	0.71740000	0.56617000
0.09030000	0.94245000	0.56606000
0.69474000	0.68726000	0.56529000
0.19086000	0.54745000	0.54546000
0.61032000	0.44616000	0.53677000
0.50246000	0.01048000	0.52284000
0.90468000	0.83127000	0.51338000
0.24808000	0.02705000	0.50716000
0.57549000	0.88409000	0.46611000
0.56343000	0.58409000	0.46025000
0.66047000	0.40031000	0.45957000
0.29769000	0.62546000	0.39337000
0.37931000	0.07442000	0.39276000
0.19496000	0.30403000	0.39047000
0.71323000	0.92717000	0.37917000

0.69881000	0.46298000	0.37684000
0.06792000	0.70326000	0.37502000
0.97309000	0.47375000	0.37223000
0.58367000	0.20933000	0.34626000
0.20380000	0.16541000	0.63499000
0.07107000	0.17350000	0.62600000
0.28346000	0.21251000	0.60001000
0.01998000	0.25781000	0.58139000
0.49911000	0.21486000	0.57271000
0.89340000	0.26628000	0.57118000
0.23723000	0.27515000	0.56160000
0.10570000	0.28610000	0.55484000
0.78582000	0.27652000	0.55834000

AIMD final state of without surfactant

1.000000000000000

10.2424999999999997	0.0000000000000000	0.0000000000000000
0.0000000000000000	8.8702000000000005	0.0000000000000000
0.0000000000000000	0.0000000000000000	30.6609000000000016

Cu	H	O	C	N
64	103	49	8	1

Direct

0.4830294160899061	0.4932457139325936	0.2702329775354134
0.1177246357110614	0.7345789605685467	0.2700426127488939
0.6151831415431988	0.2365808997893808	0.2696599780666146
0.8673741489754667	0.7393567688218853	0.2713496859328773
0.7367430877693480	0.9811608731380843	0.2739679377695693
0.4886662224550036	0.9818386606342990	0.2706694476603893
0.8625782259844174	0.2342799336272915	0.2676239106153578
0.7385428896072100	0.4883853898605611	0.2692606811834957
0.6167458635974521	0.7370927266148262	0.2712988361945091
0.1048757863847356	0.2360956066816831	0.2712707653381716
0.3657113064152621	0.2472683329629654	0.2717515344435947
0.2324983916126487	0.4874911124084593	0.2728920621956474
0.9949151570381439	0.4893429369410501	0.2725337115537720
0.2417876583683533	0.9899024766311759	0.2668193793583440
0.3644012739394673	0.7404850418817237	0.2676084221608905
0.9924745190537382	0.9841322829008370	0.2704724960102519
0.7396583493252434	0.3267271414082949	0.2010459500037433
0.4899309333430230	0.3214156954335649	0.2044147585140474
0.8704684468778372	0.5783534011027676	0.2027325735531716
0.2381329759828767	0.8260619349039664	0.1999394185070247
0.9996498668848822	0.8269911982801511	0.2016294920931817
0.9917308951667831	0.3220125923518978	0.2027705741529968
0.6081370874145258	0.0692183829076171	0.2001686138453496

0.7471370994875381	0.8275420466993114	0.2027341693387308
0.4990773189843442	0.8147332154374236	0.1990296467634878
0.1119860786839996	0.5800202647021812	0.1996437671909748
0.6152419643397332	0.5772026379879853	0.1999893808988714
0.1140124664578336	0.0765620003788922	0.2014078454338328
0.3590433484036925	0.5725294182057038	0.1999135124821977
0.2382870791199025	0.3243940889540833	0.2046470550255210
0.8597789180617450	0.0663840889269319	0.2023823511005786
0.3682937488061062	0.0809558136998298	0.2011326310305115
0.8712244282222792	0.4121016688139209	0.1350200501835086
0.3698045269021746	0.9110798462627752	0.1338774067726038
0.9969332626070122	0.6669503991774627	0.1325883480093586
0.7476124157258097	0.1576453929762371	0.1332264577673188
0.3723799178740781	0.4020137193858440	0.1320672321370076
0.6245210404471186	0.9069561878326026	0.1334391388154500
0.2411253888312633	0.6661617797927689	0.1341778536819334
0.1197721923363143	0.4100957240810489	0.1352041793624582
0.9998255091621435	0.1607805392495859	0.1353097823613210
0.7459647603190104	0.6636920869295756	0.1376490636994000
0.1230442193508370	0.9161893088494811	0.1317627090230193
0.4935585056056478	0.6577873475670658	0.1318719859204475
0.6228303670691991	0.4126447647511272	0.1339190686032390
0.2489005290905486	0.1669813390838790	0.1320130365960440
0.8724063286861112	0.9081040326116856	0.1337437350125055
0.4970521734656399	0.1612828150774199	0.1320955483002731
0.2500000000000000	0.0000000000000000	0.0652299999999997
0.6250000000000000	0.7500000000000000	0.0652299999999997
0.8750000000000000	0.2500000000000000	0.0652299999999997
0.0000000000000000	0.5000000000000000	0.0652299999999997
0.8750000000000000	0.7500000000000000	0.0652299999999997
0.1250000000000000	0.7500000000000000	0.0652299999999997
0.3750000000000000	0.2500000000000000	0.0652299999999997
0.6250000000000000	0.2500000000000000	0.0652299999999997
0.7500000000000000	0.5000000000000000	0.0652299999999997
0.1250000000000000	0.2500000000000000	0.0652299999999997
0.2500000000000000	0.5000000000000000	0.0652299999999997
0.5000000000000000	0.0000000000000000	0.0652299999999997
0.0000000000000000	0.0000000000000000	0.0652299999999997
0.7500000000000000	0.0000000000000000	0.0652299999999997
0.5000000000000000	0.5000000000000000	0.0652299999999997
0.3750000000000000	0.7500000000000000	0.0652299999999997
0.6225137761044864	0.7741709698185556	0.6683089802454267
0.6341792669957655	0.3570145403613862	0.9476047788435030
0.1008006056980729	0.2734363743003376	0.9410943879395959

0.0687791337745269	0.7674783042518568	0.9562882436035515
0.1961453347466688	0.1270509371984634	0.9356081175493534
0.3021046059075140	0.7204726820529580	0.8548589682060910
0.6952694432342786	0.8599461416042298	0.6289673478591155
0.3776484626145796	0.3510810954793995	0.8127003736061734
0.9557388725492626	0.0021774766787263	0.9188417224790459
0.8835505663184514	0.0622294521101731	0.8793699825993643
0.3902739733248323	0.8511737075660565	0.8782605774691994
0.4538749182737227	0.2042201716316134	0.8143285685031955
0.4990309533365410	0.3858801028334247	0.9282439924076068
0.0431209193435190	0.7499310780405092	0.9086359047576898
0.6969231482082552	0.5063143425095514	0.9902836065319545
0.6133851906422778	0.0252488239490952	0.8540700773588616
0.8190891876928857	0.7615803177921047	0.6916032474945907
0.8014576774169127	0.4019203133423712	0.0046562577336714
0.1100141775321892	0.9469530565415462	0.6490563127452333
0.1825294539849435	0.9756138702355097	0.6894782926417050
0.5642789469984234	0.1648688361539454	0.8793143790816846
0.7919106411934588	0.8346266101592892	0.8357772400878267
0.1465132704388494	0.5407260535090213	0.8269187661774932
0.8275246594139481	0.7651260390823287	0.7447341641613070
0.7206415129257229	0.8445206086993228	0.9675744404495756
0.5511513921505188	0.6819288934617058	0.8892143330330248
0.7446732616259958	0.8545328814533630	0.7912556309010748
0.7942389951319926	0.9813254952147509	0.9534844983930454
0.1534352893690826	0.7177900090386363	0.8062134530088084
0.5750041007277343	0.6293700620770248	0.9354981506720991
0.4412626151456020	0.6330998727865302	0.6909238302200298
0.0550646754582519	0.7931313899261607	0.7511816072171256
0.6913279528138789	0.4213949331075993	0.8205052142710964
0.4701412841660594	0.7766066022281589	0.7207051767844058
0.7722946624189716	0.2242921830644908	0.7042385316559269
0.1215979718360699	0.1406548511442320	0.7538043546235031
0.3404468079481320	0.5818575669222700	0.9030296307730651
0.5395577211415704	0.6236990478374143	0.7706913683421315
0.9643007488985339	0.3331192821944475	0.8459391705570173
0.9744788892141339	0.5708226831455016	0.7149466838726229
0.1405523678282411	0.9248505334110506	0.7704740090466610
0.7003141206485378	0.3949951338961560	0.8722231880035743
0.7074285625114665	0.1838359528710422	0.7487604560934511
0.2438821267225249	0.1714042172698266	0.7817258586698048
0.2491767156052415	0.4344380111906773	0.9133963894684844
0.5533482511416676	0.7298886915144576	0.8092365704941147
0.0769353441149103	0.2513567065011932	0.8284154048242063

0.1267689001349379	0.5128864804619060	0.7170231435051846
0.2658025565715847	0.8954289410962581	0.9521295413464966
0.4551218391556944	0.9041727466239622	0.6323393010123779
0.9871543324209053	0.6599390465058617	0.8406378315095735
0.9097355396318871	0.4345674082933007	0.6134699118851971
0.4474478414882670	0.0738205666147426	0.6899344684522374
0.8311505872634671	0.6060150893437014	0.8483119289522359
0.0421738768346172	0.4140942571275650	0.6346190374199037
0.4707681007754977	0.2616228331669978	0.9806414355785115
0.3763963112364446	0.0066790476435358	0.9724024894568468
0.3165436462106130	0.2160593049786937	0.9784024068251245
0.6446414682182769	0.3985200667104397	0.7424619580952913
0.6111956268550308	0.3687735551888940	0.4824003853606733
0.5106690538308704	0.3811571613089096	0.7699880421977203
0.7877056645820746	0.9787860539627350	0.3637994752083516
0.5318797334886106	0.8457375227348618	0.5138881174012443
0.1657622209983904	0.0215427180336617	0.3622531902375913
0.5921775711645533	0.2345156791545064	0.4533093695239716
0.3820827633740712	0.7734679275964444	0.5426827325799235
0.9685105542646117	0.3344063034279332	0.6973530394718972
0.5262788788485524	0.8312789708699757	0.5713566635818071
0.7839805815998215	0.8121131064876269	0.3818564888381677
0.2451772652657564	0.9072649431324873	0.3326235753974101
0.3378832926850795	0.2556398187549794	0.5433060071701692
0.2994138463963867	0.6269945937002175	0.3950195524895307
0.8283332401873374	0.0747502902613863	0.5739471931770201
0.2584784260924654	0.7872595677185806	0.3993658395025930
0.9754794689572465	0.1770337716664465	0.6757029950856525
0.3075969695668916	0.4396497068424723	0.6080609958648245
0.0005461876269055	0.5884407028429474	0.4707872690405226
0.0883552513503750	0.6941868618546544	0.4431988095722295
0.8580386597187070	0.9137697846779749	0.5812222127152386
0.3914743104525339	0.3506242444128090	0.3598676010101548
0.3428471346666445	0.1764863826178998	0.3574998233538361
0.2883463755121267	0.4315796558617347	0.4649475218011058
0.9059300916796588	0.9880303962416899	0.4693867702263833
0.7390458274796267	0.4774217957076038	0.5482128925586423
0.2025690258754926	0.2986070795484765	0.4839268609403989
0.0214972451760498	0.0732728085541253	0.4484258373031628
0.8359917159371237	0.3683449104103012	0.5217860451408284
0.9616412926666995	0.1845593247627700	0.5166483404706982
0.0418869182431120	0.6702786949782575	0.5811855738618484
0.6755647748350440	0.4376449362320507	0.6406310881932641
0.9877426670306632	0.6919059204191984	0.3617436980438413

0.9927295885227951	0.2740698508973345	0.5648913194460587
0.7116277772465714	0.5962286190556065	0.6241299479958240
0.0941273499267947	0.8078499293871859	0.5565935365706381
0.5171808010411015	0.4259710834804639	0.4185447289940722
0.8460180337583350	0.4562944418293178	0.3721115854564550
0.1055938456963246	0.2853384309050147	0.4121766254413810
0.6820845011602790	0.0395480569539715	0.4262649805777412
0.9347962891242075	0.6639711541541020	0.4087566507625618
0.8295088717898731	0.3297449104497838	0.3434172695345551
0.0107555630507426	0.2384377076489108	0.3716423131978668
0.5665609977710413	0.4509815628536497	0.3730732087852658
0.5553003368000740	0.9968453457611351	0.4517124989947572
0.1910604295276211	0.2415806728740574	0.9368176635885015
0.7105217468892904	0.7999021108233234	0.6555412020707776
0.8825664409090490	0.0714324652294200	0.9116364456658791
0.5644878759387660	0.3060779518639675	0.9323005479389473
0.0774494028714551	0.8256228699319886	0.9294767766465554
0.3889896278403928	0.7489743517893007	0.8655771075237469
0.4021063489043046	0.2709660780907390	0.7931115304691048
0.7751501618647092	0.4567958476216802	0.9773075319201305
0.5385026960739019	0.0913373497581486	0.8592306210082090
0.1406918645824258	0.0312280914965499	0.6653464497428941
0.8770661240158210	0.7420991546043169	0.7196527469933600
0.7386118638917791	0.9054497291739244	0.8196578834582108
0.1493386265635104	0.6525731716894559	0.8338105866910753
0.7692453085393665	0.9268806543273159	0.9797709987101955
0.6208904255313148	0.6677157245009170	0.9099984374399667
0.4789574094512309	0.7311213444684335	0.6905872043396341
0.1422496142545598	0.8172213468164526	0.7637296535461947
0.7533967786696695	0.4046207728142265	0.8450603276985741
0.6802956927819238	0.2025370554236350	0.7182924460421605
0.1661761205876884	0.1100439828682938	0.7800315559804022
0.3382661050733544	0.4708313494498096	0.9129693139872406
0.5545947021279852	0.7337462707858088	0.7772318023759628
0.0604496229846506	0.3443167866784825	0.8463544370701133
0.0344339356194045	0.4899168902574080	0.7107906662849323
0.8969587502658368	0.6871860017904756	0.8469029820898191
0.0036054520280232	0.4578506423465309	0.6083901801404250
0.3375092679426751	0.9661200815783365	0.9456892971626263
0.3994347559600564	0.2162887957267535	0.9970975296130706
0.4099489142795000	0.9959318291059801	0.5444607110756059
0.5966777366052534	0.4310228823884202	0.7677865861941036
0.2447019776240514	0.9597215002739398	0.3608638864381887
0.5445938783853552	0.3333395274188242	0.4595234096837669

0.7381240644655322	0.9109459715270083	0.3818963475330923
0.2488540245528641	0.6900783351559463	0.4149612098669992
0.9134995355710498	0.2446266469646011	0.6874230034530557
0.7865984709213175	0.9819476622325520	0.5840892297897128
0.3411933622527523	0.2708254786274049	0.3388233721109562
0.0008412212384991	0.6864726184447592	0.4560969426611976
0.7726318516926943	0.4506206283990701	0.5187328788149133
0.2024216628050852	0.3857715547770572	0.4651146632680657
0.9668761362185022	0.0738659085571525	0.4739131210790770
0.9291120492741619	0.2259693545974450	0.5451836089820101
0.0771172456422756	0.7724826058947799	0.5859577739846548
0.7360796033029814	0.4887229353598171	0.6215169197335115
0.9101918350947086	0.6588582153515671	0.3776686395722917
0.4873756721319933	0.4742112992331194	0.3901004737744725
0.0902640984635478	0.2127481724572380	0.3884799415669618
0.6271252245148394	0.0705226208692371	0.4509845424201175
0.8043825610716780	0.3553076209105979	0.3739373960079926
0.4492755982365019	0.0219302830203610	0.6230373945054096
0.4438051680046146	0.1193796954249414	0.6570832615802813
0.4019616559604003	0.0755546146929954	0.5807845029280894
0.3969926085784041	0.2669391165267077	0.6510962142916108
0.4671740851983061	0.8461534783666816	0.5422969322697065
0.3604704141898989	0.3619168091673077	0.6863650153415496
0.3706293803738435	0.2337854297325630	0.5754663757497196
0.3520280626701650	0.3252203215743168	0.6105652267956186
0.3402580173680650	0.4485916928951146	0.7139651913480359

AIMD initial state of with surfactant

1.00000000000000

10.2424999999999997	0.0000000000000000	0.0000000000000000
0.0000000000000000	8.8702000000000005	0.0000000000000000
0.0000000000000000	0.0000000000000000	30.6609000000000016

Cu	H	O	C	N
64	109	43	15	2

Direct

0.50271000	0.50257000	0.27251000
0.12884000	0.75026000	0.27216000
0.62297000	0.25196000	0.27204000
0.87849000	0.75594000	0.27146000
0.75273000	0.99964000	0.27134000
0.50035000	0.00067000	0.27118000
0.87270000	0.25755000	0.27113000
0.74794000	0.50470000	0.27074000
0.62741000	0.75097000	0.26935000

0.12215000	0.25488000	0.26921000
0.37175000	0.25581000	0.26902000
0.25621000	0.50399000	0.26849000
0.99712000	0.50462000	0.26828000
0.25116000	0.00202000	0.26791000
0.37495000	0.75298000	0.26786000
0.00222000	0.00387000	0.26739000
0.74821000	0.33581000	0.20407000
0.49861000	0.34178000	0.20260000
0.87110000	0.59014000	0.20239000
0.24822000	0.83275000	0.20229000
0.99922000	0.83831000	0.20224000
0.99926000	0.33748000	0.20213000
0.62378000	0.08277000	0.20172000
0.75293000	0.83883000	0.20147000
0.49938000	0.83539000	0.20147000
0.12503000	0.58490000	0.20132000
0.62700000	0.58772000	0.20127000
0.12627000	0.08241000	0.20038000
0.37983000	0.58732000	0.20036000
0.24766000	0.33559000	0.20011000
0.87397000	0.08675000	0.19998000
0.37350000	0.08615000	0.19854000
0.86605000	0.41938000	0.13612000
0.37307000	0.91231000	0.13506000
0.99921000	0.66466000	0.13456000
0.75160000	0.16471000	0.13437000
0.37992000	0.41581000	0.13433000
0.62532000	0.92546000	0.13403000
0.24743000	0.65937000	0.13397000
0.11780000	0.42376000	0.13392000
0.99833000	0.17032000	0.13392000
0.75263000	0.66996000	0.13380000
0.12652000	0.91323000	0.13337000
0.50058000	0.66588000	0.13326000
0.62934000	0.41809000	0.13304000
0.25040000	0.16690000	0.13270000
0.87556000	0.91919000	0.13248000
0.50231000	0.17180000	0.13111000
0.25000000	0.00000000	0.06523000
0.62500000	0.75000000	0.06523000
0.87500000	0.25000000	0.06523000
0.00000000	0.50000000	0.06523000
0.87500000	0.75000000	0.06523000

0.12500000	0.75000000	0.06523000
0.37500000	0.25000000	0.06523000
0.62500000	0.25000000	0.06523000
0.75000000	0.50000000	0.06523000
0.12500000	0.25000000	0.06523000
0.25000000	0.50000000	0.06523000
0.50000000	0.00000000	0.06523000
0.00000000	0.00000000	0.06523000
0.75000000	0.00000000	0.06523000
0.50000000	0.50000000	0.06523000
0.37500000	0.75000000	0.06523000
0.14188000	0.66203000	0.98494000
0.74391000	0.91020000	0.98480000
0.72407000	0.29325000	0.98289000
0.09434000	0.03267000	0.98145000
0.67236000	0.13193000	0.97940000
0.49241000	0.59235000	0.97914000
0.09045000	0.81660000	0.97593000
0.40312000	0.03290000	0.96417000
0.89997000	0.37927000	0.95518000
0.82818000	0.52188000	0.95376000
0.47851000	0.73522000	0.95145000
0.53162000	0.95582000	0.94994000
0.79669000	0.91037000	0.93690000
0.10842000	0.05892000	0.93327000
0.67230000	0.61867000	0.92703000
0.25510000	0.47151000	0.90705000
0.55874000	0.19544000	0.90466000
0.76841000	0.56841000	0.89023000
0.08353000	0.30937000	0.88375000
0.95285000	0.19413000	0.87879000
0.29675000	0.34746000	0.87582000
0.13336000	0.98090000	0.87014000
0.37763000	0.07835000	0.86184000
0.58004000	0.16380000	0.85543000
0.86237000	0.42695000	0.85529000
0.80494000	0.77013000	0.85118000
0.00787000	0.90096000	0.84848000
0.72776000	0.42539000	0.82546000
0.33023000	0.96152000	0.82495000
0.75734000	0.90745000	0.82272000
0.55877000	0.97248000	0.79332000
0.05960000	0.71620000	0.79228000
0.27144000	0.71253000	0.79208000

0.66392000	0.08964000	0.79122000
0.06069000	0.33666000	0.79071000
0.79880000	0.10498000	0.76270000
0.46118000	0.66844000	0.76255000
0.55881000	0.31828000	0.76255000
0.62136000	0.49755000	0.76251000
0.39441000	0.43665000	0.76233000
0.01220000	0.59936000	0.75539000
0.22404000	0.59570000	0.75520000
0.01329000	0.21982000	0.75383000
0.81404000	0.20183000	0.72111000
0.57405000	0.41512000	0.72096000
0.47641000	0.76528000	0.72096000
0.63660000	0.59440000	0.72092000
0.40964000	0.53349000	0.72074000
0.47738000	0.08260000	0.68212000
0.69404000	0.54267000	0.66052000
0.59491000	0.83204000	0.65834000
0.23076000	0.02310000	0.64722000
0.98724000	0.15280000	0.64358000
0.60920000	0.41557000	0.64340000
0.51007000	0.70494000	0.64122000
0.84437000	0.83760000	0.63791000
0.49970000	0.03672000	0.63214000
0.75953000	0.71050000	0.62079000
0.00586000	0.71704000	0.61740000
0.42036000	0.96483000	0.60530000
0.09748000	0.99527000	0.59410000
0.04035000	0.57295000	0.58976000
0.75670000	0.70452000	0.58809000
0.59739000	0.15456000	0.57661000
0.37597000	0.81596000	0.56514000
0.49867000	0.33547000	0.56502000
0.01882000	0.87284000	0.56335000
0.61779000	0.87538000	0.56267000
0.33752000	0.01702000	0.55337000
0.64144000	0.54405000	0.55083000
0.58030000	0.07640000	0.55043000
0.44612000	0.13793000	0.54915000
0.30455000	0.35847000	0.54516000
0.75364000	0.74315000	0.54473000
0.08074000	0.38819000	0.53184000
0.22875000	0.63146000	0.52755000
0.19428000	0.46717000	0.52396000

0.68686000	0.40531000	0.52374000
0.49375000	0.79530000	0.49476000
0.73616000	0.84678000	0.49344000
0.88735000	0.91606000	0.49322000
0.94369000	0.75940000	0.49195000
0.45972000	0.99647000	0.48173000
0.70489000	0.04802000	0.47959000
0.66264000	0.30682000	0.47701000
0.66637000	0.64590000	0.44365000
0.49869000	0.72691000	0.44275000
0.72289000	0.39085000	0.43547000
0.29889000	0.65570000	0.42576000
0.38406000	0.06628000	0.42563000
0.85529000	0.80592000	0.42488000
0.19615000	0.33428000	0.42285000
0.46449000	0.99083000	0.42259000
0.82990000	0.00688000	0.40957000
0.60606000	0.11243000	0.40796000
0.58791000	0.69421000	0.39064000
0.34140000	0.53040000	0.38716000
0.23866000	0.20897000	0.38426000
0.44946000	0.13859000	0.38257000
0.79330000	0.45177000	0.37404000
0.77736000	0.85402000	0.37185000
0.55367000	0.95879000	0.37046000
0.62930000	0.28232000	0.36615000
0.15248000	0.65352000	0.36444000
0.05764000	0.42401000	0.36165000
0.64298000	0.12448000	0.35115000
0.04625000	0.77254000	0.35059000
0.66193000	0.51050000	0.34889000
0.95142000	0.54303000	0.34780000
0.64793000	0.23002000	0.99451000
0.06460000	0.72195000	0.99073000
0.84638000	0.43698000	0.97330000
0.74808000	0.96826000	0.95806000
0.14119000	0.99147000	0.95731000
0.49510000	0.62676000	0.94900000
0.43687000	0.94580000	0.94696000
0.76285000	0.64451000	0.91740000
0.21585000	0.40667000	0.88421000
0.97972000	0.30873000	0.87881000
0.51867000	0.20638000	0.87558000
0.03991000	0.97822000	0.87097000

0.30415000	0.03839000	0.84560000
0.78743000	0.49110000	0.84225000
0.83579000	0.85140000	0.83224000
0.62763000	0.01297000	0.81108000
0.98612000	0.67624000	0.77605000
0.19796000	0.67258000	0.77585000
0.98720000	0.29670000	0.77448000
0.76620000	0.20341000	0.74850000
0.42858000	0.76687000	0.74836000
0.52621000	0.41671000	0.74836000
0.58876000	0.59598000	0.74832000
0.36181000	0.53507000	0.74814000
0.65208000	0.45207000	0.66967000
0.55295000	0.74144000	0.66749000
0.54598000	0.03756000	0.66137000
0.80241000	0.74701000	0.64706000
0.41339000	0.16760000	0.60570000
0.96590000	0.65219000	0.59716000
0.09030000	0.94245000	0.56606000
0.69474000	0.68726000	0.56529000
0.19086000	0.54745000	0.54546000
0.61032000	0.44616000	0.53677000
0.90468000	0.83127000	0.51338000
0.66047000	0.40031000	0.45957000
0.29769000	0.62546000	0.39337000
0.37931000	0.07442000	0.39276000
0.19496000	0.30403000	0.39047000
0.69881000	0.46298000	0.37684000
0.06792000	0.70326000	0.37502000
0.97309000	0.47375000	0.37223000
0.58367000	0.20933000	0.34626000
0.20077000	0.12446000	0.62824000
0.06855000	0.14604000	0.62036000
0.28247000	0.19740000	0.59780000
0.02096000	0.26919000	0.58356000
0.89490000	0.29196000	0.57451000
0.49846000	0.21439000	0.57123000
0.41089000	0.93720000	0.56913000
0.23892000	0.29394000	0.56538000
0.10795000	0.31626000	0.55986000
0.54460000	0.95532000	0.54682000
0.53108000	0.91579000	0.49815000
0.66630000	0.92799000	0.47651000
0.59854000	0.72827000	0.42631000

0.78746000	0.88829000	0.40752000
0.56420000	0.99355000	0.40608000
0.78794000	0.31792000	0.56303000
0.65476000	0.88519000	0.42902000

AIMD final state of with surfactant

1.00000000000000

10.2424999999999997	0.0000000000000000	0.0000000000000000
0.0000000000000000	8.8702000000000005	0.0000000000000000
0.0000000000000000	0.0000000000000000	30.6609000000000016

Cu	H	O	C	N
64	109	43	15	2

Direct

0.5003015036685197	0.5031304030269955	0.2669927622031745
0.1261873942890793	0.7550528743176603	0.2724519095911718
0.6212574903653345	0.2484114285978271	0.2755916850537574
0.8745504173617428	0.7507007917476969	0.2722998667049207
0.7488216072685121	0.0034615894629552	0.2695026694683668
0.4974333378072026	0.0029872249799410	0.2686569264631785
0.8730212402097753	0.2599083110332480	0.2677061940473157
0.7503122915412023	0.4981856914071096	0.2709886491832543
0.6252239097712931	0.7471426508653298	0.2697939678218879
0.1266265329086080	0.2601871077884534	0.2688673278971739
0.3770219306674450	0.2529846646025720	0.2692454430615382
0.2522753706571943	0.5073255300030901	0.2697741799063209
0.0016606985762457	0.5074852383640964	0.2708100111402227
0.2531563927520059	0.0099475812427597	0.2682967238901333
0.3732458675580294	0.7552703278319897	0.2713330055880835
0.9991718958738351	0.0050682789434649	0.2680277086814263
0.7501217832607052	0.3348717573269538	0.2010391013100159
0.5054090936613589	0.3333364823530691	0.2005184930539639
0.8737699662940938	0.5852206377487188	0.2019522955710970
0.2491973938028792	0.8288715780390244	0.2012734423153861
0.9994863707961966	0.8343965664414196	0.2019246321896383
0.0004110912561963	0.3410347018423692	0.1994259915166632
0.6253327485469464	0.0903788232395894	0.2022575200013128
0.7528185821257644	0.8392281586401048	0.2022904320066790
0.5015602478041423	0.8356292190295687	0.1994866506000514
0.1251097446924371	0.5837530654810519	0.2009880474123399
0.6261993419465591	0.5853088569225278	0.1988107124640021
0.1249017276838780	0.0864856287754144	0.2003667172584711
0.3758249563705117	0.5855579994188340	0.1993369681030814
0.2519757984885382	0.3387002861135424	0.1983533467184032
0.8756486270258488	0.0892834451438875	0.2027923929179921
0.3748479406821817	0.0812512232486212	0.1992319987678179

0.8741307460973761	0.4209980157661655	0.1333270407676401
0.3723986188787008	0.9161969490453257	0.1332534748928857
0.9963325731232547	0.6686562045850380	0.1340965060300524
0.7492655067048790	0.1703084826133869	0.1349381645542846
0.3782518993509204	0.4125467138678683	0.1321031508031123
0.6241907295671103	0.9179402446168217	0.1341868659463325
0.2505482451931992	0.6673115765938468	0.1328646540310311
0.1276927562647173	0.4191614966467889	0.1326872216329955
0.9995097177098362	0.1684694896476203	0.1322291625541850
0.7497368657120843	0.6700287382247913	0.1346856394407271
0.1243387050604369	0.9148026612945395	0.1349103178354830
0.4962111794246009	0.6650871684244194	0.1333043823666278
0.6232344758469802	0.4204265454289566	0.1323160246073922
0.2512533560894755	0.1665273161618434	0.1326814075489428
0.8763678567915547	0.9226131460382913	0.1348979814706740
0.5003331428317512	0.1675285297504804	0.1326251304204921
0.2500000000000000	0.0000000000000000	0.0652299999999997
0.6250000000000000	0.7500000000000000	0.0652299999999997
0.8750000000000000	0.2500000000000000	0.0652299999999997
0.0000000000000000	0.5000000000000000	0.0652299999999997
0.8750000000000000	0.7500000000000000	0.0652299999999997
0.1250000000000000	0.7500000000000000	0.0652299999999997
0.3750000000000000	0.2500000000000000	0.0652299999999997
0.6250000000000000	0.2500000000000000	0.0652299999999997
0.7500000000000000	0.5000000000000000	0.0652299999999997
0.1250000000000000	0.2500000000000000	0.0652299999999997
0.2500000000000000	0.5000000000000000	0.0652299999999997
0.5000000000000000	0.0000000000000000	0.0652299999999997
0.0000000000000000	0.0000000000000000	0.0652299999999997
0.7500000000000000	0.0000000000000000	0.0652299999999997
0.5000000000000000	0.5000000000000000	0.0652299999999997
0.3750000000000000	0.7500000000000000	0.0652299999999997
0.4954421708602467	0.4547193346616933	0.7092272958764470
0.7126252669793037	0.1599350181954081	0.9770848020868518
0.0397781871331031	0.3007039445863214	0.7987898702943121
0.9453524869089006	0.7854392515167565	0.8778454070679851
0.0455584562636526	0.2214961566871723	0.8444067540192817
0.5656649393511328	0.4496001866806049	0.8524114732716300
0.4891526488606832	0.5891997943449840	0.6753625133379878
0.3265463596697091	0.3355344003535105	0.9402773610589100
0.7435526223483124	0.9322269973322000	0.8647169516233856
0.6500471190821439	0.8921065897021614	0.8231602478119830
0.5975264960842092	0.5660601013849635	0.8872513921523464
0.2812468638082327	0.1779111784461113	0.9450173277359465

0.6720021435036601	0.0770235374635711	0.9354521356618861
0.8580606039368226	0.7844818847343421	0.9205204729867901
0.1971662687939405	0.5462453342886840	0.9835137541189664
0.2117949586565937	0.9134670520940527	0.7378251466751639
0.6084760583497336	0.9026877862121400	0.9718029954456747
0.3333138061977015	0.5988628309715329	0.9689899297809360
0.2253503252166779	0.8600833324797772	0.9808988002070615
0.1909073015240077	0.8612899995094556	0.9310566094240110
0.3358054563524255	0.9768027697370839	0.7573945419687598
0.7441012429818564	0.2861170154376236	0.8034933213326559
0.6766622880279288	0.6231012130753485	0.9490203268087148
0.4867411594718677	0.8391239678080658	0.9518599808814989
0.7289343043747487	0.7564312736729175	0.7536103608414386
0.7898821099638402	0.3805960563470355	0.6552530580841517
0.6705485718623054	0.1924279249256622	0.8422265198878246
0.6875589672527082	0.6507887957924418	0.7180654911789349
0.6952209578999056	0.4781640954454183	0.9785269340484285
0.7686023289765110	0.5287188750399296	0.6704385200680477
0.6703969686734581	0.8991821371334129	0.5589003270188501
0.0182721563877783	0.8177450405883459	0.7677237967386351
0.0090143539381979	0.8691244828034459	0.9850399337934328
0.6479001937321788	0.9355989981889195	0.6052117531207198
0.8472905029267540	0.9706975409443693	0.8053798307132632
0.4702619072169583	0.8398696987753480	0.6603332016992565
0.4264913206163474	0.2858622235169156	0.7717053131728174
0.8810201345605916	0.2327060549390574	0.9245849736038874
0.0353652541816166	0.9217377596592172	0.8341888533174847
0.5212787263070818	0.6612517229658756	0.7638713789789190
0.1111726839073670	0.6861061327306819	0.7520500720347656
0.9571218777345151	0.7014444451019729	0.9875347122791864
0.9445271470984123	0.0827099431936142	0.7837750401930297
0.3166561310794875	0.8124933179451517	0.6456482358663063
0.9606521149544316	0.3933056294622009	0.9253000871348146
0.3694158491183259	0.2176328652574706	0.7279250421120588
0.1257838833777181	0.7788641241872447	0.8283163718922234
0.5555646567329174	0.6326256913267091	0.8127449102352392
0.7644364962420294	0.2454186249331329	0.7248577979274404
0.6782975100433603	0.8467322638914341	0.6774948740341102
0.9118886900051318	0.4407805554909818	0.7919845062490780
0.0573562953214526	0.1693408009658734	0.6765988525911621
0.9510863428423139	0.9287952173206883	0.6611531700867542
0.6428448944813857	0.0195876130196342	0.6726786900991916
0.9510181599789033	0.4275280215693253	0.7451244339370670
0.2670319771919629	0.2529383873604092	0.8204273172538261

0.6181189804274807	0.2764112704657478	0.7167804262895714
0.3691339571574481	0.2355435860488841	0.8559842607639226
0.4050129091228484	0.3971754208639994	0.4820727737568443
0.9708465602802090	0.2941286151543386	0.5666793366426547
0.0413438809478495	0.1663944219925062	0.9556378455677929
0.3806247552516925	0.2246550734174990	0.4968865376105943
0.1407323383289962	0.5050671649527489	0.6921275177698337
0.4349208123728882	0.2391659948160446	0.6227913156211594
0.0715192949192265	0.2374492501489272	0.5189748747353675
0.4021239148446326	0.4291827317525704	0.6375089450907440
0.1011539942314667	0.1276198485221467	0.0043630930814288
0.7787281120743734	0.2055796997292816	0.5320091565258416
0.9994626351654586	0.0982847555152451	0.5484241864643752
0.6437011419099475	0.9754343430741502	0.4044735259491279
0.8528552363390082	0.1187260368218219	0.4877263137128537
0.3341537738487585	0.3614993991359511	0.5885778273035148
0.3147890583123418	0.1424932861499250	0.5735364792474357
0.9916245707573352	0.5183319053980630	0.6887937114481254
0.2280582840196110	0.8870507347094674	0.5536603459688410
0.4292141564996840	0.7386038986607645	0.5929045982756567
0.3814197426044724	0.7227396355639569	0.5442449134263532
0.4951581014885076	0.9656187478310524	0.4163576198647897
0.7657478336349359	0.3259469269015506	0.4594219609408525
0.0192680587770890	0.3418714786057487	0.4418283044114299
0.6103284968426075	0.6368256612695695	0.5580973959806257
0.7250480854334914	0.5575585733538988	0.5848665455662647
0.7994833478939471	0.4425136011891683	0.5037865607242916
0.0299771154527985	0.4628541584836022	0.4872695542297896
0.8537189399462415	0.6277342805180474	0.8518152181205432
0.8356699428843209	0.4229742327242568	0.3934843570785839
0.7214840394357880	0.5458637687717028	0.4248937176337120
0.7946588366032887	0.4707128280551615	0.8452954172523364
0.3124709977778587	0.2728694486060871	0.4248194071552079
0.7731719217887065	0.8382708947672562	0.3436193941062821
0.0503492315506366	0.5121828855370441	0.3807014281332453
0.5909262738346429	0.2284419805404533	0.4092946795475801
0.8911046916245132	0.7995200797877393	0.4462850801077476
0.1383603980194963	0.6197040404068025	0.4205571486374141
0.8127940539310911	0.6750199177660354	0.4820315153636529
0.8121484928816807	0.6198556850612833	0.3791133336840443
0.3326315163027860	0.2762724953489187	0.3745235923673589
0.5690101552350156	0.3458538613997449	0.3768443228879250
0.8712934781589402	0.9417791671417316	0.3716543627451079
0.9984581129096927	0.1884809995105231	0.3394086840621565

0.0256890931023603	0.7147401675240652	0.3869772573497417
0.9881402636134250	0.7031032289606287	0.4872435125165878
0.5587902706196082	0.0279531113036003	0.4921282366466581
0.2357441323139862	0.9064473079317671	0.3649177936144928
0.3949925371202128	0.9391149513150786	0.4927487894479457
0.5296568916352360	0.0661442613575845	0.5391207628331719
0.2384749936024171	0.0326939293465076	0.4015473288615415
0.0830323915821886	0.1822783906023419	0.3814843032811634
0.3064264999199947	0.8619312493347326	0.4611375644481416
0.0708509013691822	0.2139392524680155	0.8132075940924588
0.5460989517835242	0.5125058132659533	0.6884120916074145
0.6936755008732886	0.9746573920661608	0.8397320633255461
0.7463180226186308	0.0935149398177743	0.9536052119505299
0.8516777962918383	0.8055218764974585	0.8887517457890466
0.5482123082905018	0.5548639367985023	0.8598007896964621
0.3418578234847787	0.2365800151130548	0.9271769336948610
0.2896524833464464	0.5296137482717098	0.9898488835571452
0.2720458312952545	0.9995730060747388	0.7341683440153252
0.2479703398467644	0.8200407466712941	0.9531883147955340
0.5795289670983206	0.8173718037558295	0.9529199176243563
0.7052301623927136	0.2901602718300579	0.8332066746673122
0.7012608539823528	0.5168245751066594	0.9482300758154075
0.7524068780228100	0.7343008030626729	0.7241612651651594
0.8364558060118608	0.4744238845037300	0.6545585261362789
0.6914404209199125	0.9795137834507022	0.5793851853521153
0.1094873277935599	0.7801422080972347	0.7684538732379764
0.9338564061676476	0.8013336561182626	0.9769841403988995
0.9161262411027428	0.9770119116926539	0.7841676866172148
0.4069240861354236	0.7769464491999504	0.6433193174276381
0.4456761837877065	0.2717891212633273	0.7403824023739478
0.9657829637074262	0.2826497506871473	0.9169064422389734
0.0819547998564927	0.8410632391506085	0.8498392102040198
0.5437904126275043	0.7117736159638277	0.7903972846069075
0.6228479592596059	0.9193896804235772	0.6612492899842483
0.9054922238105524	0.3671077234303539	0.7668620008253617
0.6944886980932041	0.2209898648673910	0.7048558426083860
0.3617887952240502	0.2381647446639557	0.8243720120887599
0.2490024067098252	0.2860184297749121	0.6433082593730592
0.3397044230395631	0.3166214331141070	0.4849806408102015
0.0942741985325296	0.0972854869023144	0.9736899672249038
0.0642821123115570	0.5407353500289798	0.7079267462436547
0.4594488734790146	0.7180241497028008	0.5625661512285790
0.5809496258798891	0.0094423433117441	0.4252775795647157
0.7094739621951552	0.6208311776331582	0.5577087742018308

0.8722075568088038	0.5263897872967965	0.8400473421414825
0.2686021863968960	0.2452492730788084	0.3971214185588327
0.7976338905522785	0.8673145725679460	0.3738400340975216
0.5877304008142433	0.3384601336942629	0.4081115512547902
0.9930956008690912	0.1971224055942812	0.3710261817575381
0.2480006351081601	0.9231879547049843	0.3966749324019730
0.3305223762131823	0.8584095620839661	0.4916229840977928
0.5065096854093795	0.0964498408860880	0.5098239106772260
0.0927473187387363	0.0957822933117680	0.6518524909300913
0.0320292359726015	0.9664311466926132	0.6407960363944232
0.1976448150536377	0.1538476359897205	0.6300661475401564
0.0862745290261650	0.8830370065814601	0.6057422871383243
0.0571439865161136	0.7252965646983613	0.5956189592229995
0.3633858498449184	0.3298594209174660	0.6219776475716834
0.9833826259123397	0.2178966956231046	0.5386832843810911
0.2363494164403890	0.0894172746280956	0.5906300964319404
0.1911571636338077	0.9465745561300577	0.5822888155677235
0.8643748835063214	0.2132190391851345	0.5109686310334220
0.8449791939655069	0.3577425048871470	0.4832762510035060
0.9657615063431857	0.4239961169647413	0.4614551589060477
0.8139775282203019	0.5373940776803479	0.4040850298430014
0.0456238179630817	0.6084126708087069	0.4036765613194760
0.9049067505279133	0.6946903010991434	0.4654046909063107
0.0548024514079178	0.6042335742535404	0.5799974040037520
0.9352849323351421	0.5680610295875110	0.4325010187690053

The molecular transport initial state of without surfactant

1.00000000000000

10.2424999999999997	0.0000000000000000	0.0000000000000000
0.0000000000000000	8.8702000000000005	0.0000000000000000
0.0000000000000000	0.0000000000000000	30.6609000000000016

Cu H O C N

64 103 49 8 1

Selective dynamics

Direct

0.5026758429288477	0.5127199650831227	0.2642605204474652	T	T	T
0.1259184639001965	0.7579541358749569	0.2651314705504300	T	T	T
0.6313422733064707	0.2600419078035051	0.2644762702638608	T	T	T
0.8769649288977553	0.7581829196088331	0.2642028774672431	T	T	T
0.7556275906924794	0.0083491870790786	0.2647328716634000	T	T	T
0.5048846387463750	0.0046225330045889	0.2688343669128180	T	T	T
0.8775001338279457	0.2583503925793664	0.2652587376490707	T	T	T
0.7541478745183547	0.5080884134423597	0.2651190984981018	T	T	T
0.6304463402698567	0.7548784108317785	0.2648529799710347	T	T	T

0.1256306510555633	0.2579482788457347	0.2648468067927866	T	T	T
0.3751045565852917	0.2624490017326332	0.2710794697843153	T	T	T
0.2492108753719050	0.5107985140245700	0.2650711855747089	T	T	T
0.0015548368645687	0.5079077092873673	0.2636221700163924	T	T	T
0.2489425366454306	0.0075132871620901	0.2651745121740750	T	T	T
0.3740645363735800	0.7567858393089555	0.2652475464420221	T	T	T
0.0018921109630856	0.0089176566292794	0.2648630253755458	T	T	T
0.7507085222075783	0.3404272349108176	0.1977271788975020	T	T	T
0.4994889637940059	0.3377218113659818	0.1996591870234068	T	T	T
0.8760467717363074	0.5902311650740305	0.1971103029182334	T	T	T
0.2504262372629728	0.8402640789551020	0.1979782458874324	T	T	T
0.0013008902967184	0.8390454963018468	0.1975519836899515	T	T	T
0.0018855879892835	0.3389416993572865	0.1972195040871457	T	T	T
0.6245587751224730	0.0890984574331435	0.1990556722823951	T	T	T
0.7508507897913751	0.8401100798731818	0.1977169252975597	T	T	T
0.5009861863328156	0.8390964163848744	0.1994147890821050	T	T	T
0.1260020130435459	0.5900521021204030	0.1971899004397051	T	T	T
0.6266647445605442	0.5894961772720764	0.1979354918290867	T	T	T
0.1264519102373940	0.0898832654917679	0.1978724215261519	T	T	T
0.3750267677811094	0.5895833128276915	0.1979481866235440	T	T	T
0.2531273588915933	0.3396077886484398	0.1992352591197027	T	T	T
0.8758146969602599	0.0895737106083847	0.1976960047962676	T	T	T
0.3773457212604618	0.0911910314082151	0.2010499550261126	T	T	T
0.8746921386807934	0.4195922088682716	0.1322100140185333	T	T	T
0.3755027420618861	0.9217537210921976	0.1334357732449578	T	T	T
0.0007146811935429	0.6703178487422948	0.1317608590137100	T	T	T
0.7492391341615926	0.1691697298440205	0.1328752475828750	T	T	T
0.3757981285712143	0.4195658289952344	0.1323815777003854	T	T	T
0.6251668030162935	0.9199365174289036	0.1327951259904137	T	T	T
0.2509391720802587	0.6698331603183389	0.1322190325924889	T	T	T
0.1271947092665018	0.4189564921550238	0.1323533757182420	T	T	T
0.0005688852078784	0.1693578050396514	0.1322145925973128	T	T	T
0.7494922636992770	0.6691328828116155	0.1323097859731072	T	T	T
0.1258690565635368	0.9197697717704788	0.1322414279815720	T	T	T
0.5000849781861109	0.6707592648715539	0.1323056369080985	T	T	T
0.6246872977030794	0.4190918624279606	0.1326431329215912	T	T	T
0.2521398917636536	0.1692582777351821	0.1333754398311741	T	T	T
0.8748962915971168	0.9197613708025943	0.1321992099851504	T	T	T
0.4999315004166854	0.1710354252234358	0.1329204203659650	T	T	T
0.2500000000000000	0.0000000000000000	0.0652299999999997	F	F	F
0.6250000000000000	0.7500000000000000	0.0652299999999997	F	F	F
0.8750000000000000	0.2500000000000000	0.0652299999999997	F	F	F
0.0000000000000000	0.5000000000000000	0.0652299999999997	F	F	F
0.8750000000000000	0.7500000000000000	0.0652299999999997	F	F	F

0.1250000000000000	0.7500000000000000	0.0652299999999997	F	F	F
0.3750000000000000	0.2500000000000000	0.0652299999999997	F	F	F
0.6250000000000000	0.2500000000000000	0.0652299999999997	F	F	F
0.7500000000000000	0.5000000000000000	0.0652299999999997	F	F	F
0.1250000000000000	0.2500000000000000	0.0652299999999997	F	F	F
0.2500000000000000	0.5000000000000000	0.0652299999999997	F	F	F
0.5000000000000000	0.0000000000000000	0.0652299999999997	F	F	F
0.0000000000000000	0.0000000000000000	0.0652299999999997	F	F	F
0.7500000000000000	0.0000000000000000	0.0652299999999997	F	F	F
0.5000000000000000	0.5000000000000000	0.0652299999999997	F	F	F
0.3750000000000000	0.7500000000000000	0.0652299999999997	F	F	F
0.1727750828054780	0.9189794090327524	0.7671461636190182	T	T	T
0.2726738128339526	0.1445965387180006	0.9888035987829924	T	T	T
0.5969833390100681	0.5464713047264924	0.9846608220156584	T	T	T
0.5969818657933145	0.0307623341828250	0.9414221716901956	T	T	T
0.4428249586615598	0.4974408985488598	0.9828175965659264	T	T	T
0.0189048542706225	0.9245773121749202	0.8138358357386839	T	T	T
0.2556678658200351	0.0366451629770310	0.7413128566700140	T	T	T
0.9220755312490896	0.4525346435693022	0.8612012214490979	T	T	T
0.7729602213475620	0.6692757666939932	0.9926531535313204	T	T	T
0.7217880488954026	0.7326654290994150	0.9470973129232192	T	T	T
0.1280278088083731	0.0011860642530192	0.8420985446889887	T	T	T
0.8910719767167976	0.5695021641878442	0.8233057836972716	T	T	T
0.3958009307344402	0.1537906197212976	0.9572963629448225	T	T	T
0.5944361962797415	0.1497797295412588	0.9798964665666218	T	T	T
0.1963346572548628	0.2580801373123689	0.8691665947093173	T	T	T
0.8300112818111487	0.4958644589786790	0.9328502568586839	T	T	T
0.2069076233875892	0.5371303860177014	0.8661795582447501	T	T	T
0.2200039955061175	0.1478054609403008	0.9092834010765536	T	T	T
0.5509526385443253	0.8569116577067115	0.7105566536868936	T	T	T
0.6364900746143857	0.9985916826273280	0.7022583058616113	T	T	T
0.8992688719555093	0.3357673160653583	0.9340798748167366	T	T	T
0.2907728579337707	0.3759220770366021	0.9592296064290640	T	T	T
0.7755017960984983	0.2518816255591705	0.7590965827826472	T	T	T
0.1180537384468183	0.4540284193746398	0.8336281138604911	T	T	T
0.4828308525099378	0.8752234328102104	0.8429238145236180	T	T	T
0.0058434690694837	0.2068765909816578	0.9885988507399180	T	T	T
0.3188329208030788	0.5189358176119382	0.9296225082211408	T	T	T
0.4973530643940495	0.6921443650743654	0.8459710622051286	T	T	T
0.6776975136910931	0.2001690139890063	0.7960875532411276	T	T	T
0.8818994280087922	0.1171183928904518	0.9785794956954184	T	T	T
0.8191694968613621	0.0310757208448482	0.7709140422625894	T	T	T
0.3675162493577049	0.4703540391980680	0.8312994397530357	T	T	T
0.9463718339214532	0.0813124630971491	0.8967044868506548	T	T	T

0.9172228164973864	0.9413094334377456	0.7409290015112308	T	T	T
0.3668864370734692	0.1445851837012045	0.7871959408418394	T	T	T
0.3367006815532677	0.0773704235963787	0.8524579801570911	T	T	T
0.1654505147393055	0.6214380356790472	0.9750967830473573	T	T	T
0.9872412770085934	0.6983524512947737	0.6899993495627222	T	T	T
0.5530144930293299	0.3423835408802653	0.8520665047495198	T	T	T
0.4769773566016338	0.7968332078778515	0.7759865946073302	T	T	T
0.4883610087025798	0.4937676484373332	0.8004344587999922	T	T	T
0.8418197554742414	0.1383256548727377	0.8640330944121241	T	T	T
0.2641222336445908	0.2608396194872429	0.7691430894116605	T	T	T
0.4854971470831970	0.1192542209529976	0.8447283815922103	T	T	T
0.0227765188725733	0.6849003173105963	0.9728364838262852	T	T	T
0.8459222341770233	0.6807342608302873	0.7093435118372898	T	T	T
0.6072515188910961	0.2393482456791594	0.8903319130917622	T	T	T
0.3574964302434950	0.8295401912086592	0.7450278447178484	T	T	T
0.6315195216522287	0.8431160283858011	0.8914870877766896	T	T	T
0.6369480876972197	0.0988454787014807	0.6198734651981608	T	T	T
0.7049598358438957	0.7340428739345864	0.8213990192188106	T	T	T
0.1820486925418396	0.8690684884944911	0.4698647851321449	T	T	T
0.4211283845686347	0.2207661310776856	0.6312716903134028	T	T	T
0.8227073843960476	0.7909912149961865	0.7926764571075583	T	T	T
0.1569599649521354	0.8258840300120773	0.5188142216699314	T	T	T
0.1677342422856779	0.7241585304505739	0.9139394757757888	T	T	T
0.7601992491556010	0.9369687422030316	0.9024361638524010	T	T	T
0.1733923178843486	0.7732692021448324	0.8640840811524340	T	T	T
0.9179596934891452	0.4446932825361092	0.7318467155463358	T	T	T
0.6632915141326564	0.3008523770183902	0.4271379273945213	T	T	T
0.9297062721341484	0.3881020048459494	0.7800124394718067	T	T	T
0.2563529262366652	0.0943715364353976	0.5415641207096890	T	T	T
0.9970814616443910	0.3679629962521724	0.6507779316526530	T	T	T
0.1125116627641926	0.3211440030344414	0.4013876094008937	T	T	T
0.7431758053470309	0.1510460020419304	0.4154117325872930	T	T	T
0.9748577199246105	0.4017394607764598	0.5932524956428481	T	T	T
0.1684610383178225	0.7305272219076057	0.6567531148112906	T	T	T
0.0545909008937847	0.2323584138715585	0.6114592258284580	T	T	T
0.3075240125448525	0.0933257482749816	0.4934369589550525	T	T	T
0.2410065868216925	0.3505026533983850	0.4283465792502279	T	T	T
0.8270246858270405	0.5298061668447344	0.6378997957701267	T	T	T
0.6064341228003005	0.5213784049666953	0.4745850405333335	T	T	T
0.2536830769272485	0.5023933269163260	0.4937459556641758	T	T	T
0.4655119539546290	0.4427421404266056	0.4694610729629654	T	T	T
0.1438893859899450	0.8696362263787414	0.6871147337554684	T	T	T
0.6137293043972722	0.6508057021932001	0.6493628279268040	T	T	T
0.0184047435273545	0.6671424928109213	0.4806550502579238	T	T	T

0.9577332750409930	0.5228082303368357	0.4557756166779560	T	T	T
0.2903114971232768	0.3290194176646822	0.5002682692146843	T	T	T
0.4444015839231477	0.3857037508566364	0.3492994220666329	T	T	T
0.3188087675648096	0.2880601389043303	0.3585002128153222	T	T	T
0.6766252659481121	0.8209736832309377	0.5259431377519637	T	T	T
0.8732745012186306	0.3455196519447755	0.4084157297701233	T	T	T
0.8866060331851399	0.9589778653533392	0.4145138536760690	T	T	T
0.5822259521620412	0.9608995325061260	0.5253551385147081	T	T	T
0.9448143203494952	0.4910503311520422	0.3893540367400105	T	T	T
0.8306712635047274	0.0058308575794677	0.4604380848739372	T	T	T
0.7080954281976298	0.5825507659128549	0.5349334801810846	T	T	T
0.7862869993491045	0.0318730941233671	0.5241474156803625	T	T	T
0.1469992441397595	0.9021912552364200	0.6011931943311223	T	T	T
0.8410985589033171	0.6974265934262125	0.3632229749858979	T	T	T
0.8013834897955118	0.6273319822564143	0.4961184021154330	T	T	T
0.2823788711210066	0.8749199277364585	0.5802608833466519	T	T	T
0.8479533455043072	0.1876646925393096	0.5093848373313472	T	T	T
0.4211283809109220	0.6123216236414049	0.3776473006473999	T	T	T
0.5305035128619067	0.0973333327482497	0.3489760556729809	T	T	T
0.3303319449411863	0.9682278620681706	0.4209563710499106	T	T	T
0.6219882819012889	0.7010801528408217	0.3759667471112149	T	T	T
0.9531174799037270	0.6403769519461602	0.3326547794022180	T	T	T
0.5910607664700827	0.9282882190416054	0.3489869386251702	T	T	T
0.2381228023777252	0.1107033039097090	0.4249866389538807	T	T	T
0.5192759449942838	0.5177818217626902	0.4072086242374869	T	T	T
0.7063859836340097	0.8295766338674112	0.4008135878426752	T	T	T
0.5284973648453329	0.4817187397000304	0.9982995960183616	T	T	T
0.2160233725632342	0.9331741078640730	0.7384825399628413	T	T	T
0.7390566454872952	0.6371801355398962	0.9639808112047904	T	T	T
0.3012397944896392	0.1837214410596639	0.9602142775764132	T	T	T
0.5580320267048920	0.1290191186118698	0.9508998162143582	T	T	T
0.1098714862527689	0.9115062151122816	0.8239888248951577	T	T	T
0.9473170296930020	0.4803016364730239	0.8307975875683365	T	T	T
0.1983658190229228	0.1495621478062529	0.8776826125219835	T	T	T
0.8756190830530093	0.4204831559302684	0.9146638376883038	T	T	T
0.6141253114563409	0.9015326363313308	0.6894499127001257	T	T	T
0.2054187292409435	0.4426644755125248	0.8476993519855170	T	T	T
0.2993414010243708	0.4885669850815373	0.9595497098627740	T	T	T
0.7113896707102978	0.1710224587826254	0.7674023934559788	T	T	T
0.5455921891625208	0.7890646125644947	0.8459979781288024	T	T	T
0.9531881852177274	0.1702722240546174	0.9638562901692728	T	T	T
0.8884787611426059	0.9501842955502192	0.7710880803042494	T	T	T
0.4628640364911756	0.4934223258092997	0.8310894979374392	T	T	T
0.8782633717548523	0.0465547289547923	0.8767613736462743	T	T	T

0.3336322498590191	0.1927365779459407	0.7599328050277298	T T T
0.4142953339980536	0.0500040836808438	0.8342464032121637	T T T
0.1141014177096106	0.7139943375714222	0.9693513696477448	T T T
0.9302938160565412	0.6304761797367447	0.7072910024429108	T T T
0.5993782654697459	0.2455648474572182	0.8585060535398619	T T T
0.4428097274682499	0.7748783765014637	0.7469686211276245	T T T
0.7848284210934940	0.6995187311841773	0.8066570708982760	T T T
0.1561546237387375	0.7844920869731489	0.4889407611115378	T T T
0.6847149337859675	0.8837847308827770	0.9162959192947576	T T T
0.2168873282802716	0.7094578471119201	0.8863100228721010	T T T
0.8581557193742038	0.2296652101710503	0.6199035810292457	T T T
0.9137093229437976	0.3519814196630982	0.7497749409631848	T T T
0.2064432263772769	0.2902235949484470	0.4034563854825270	T T T
0.7241233741947313	0.2560386537820357	0.4059218905916087	T T T
0.3115927297864451	0.1510426934667151	0.5211349984973677	T T T
0.5544265482213248	0.4499852093192428	0.4563537181241132	T T T
0.1073931010667807	0.8159806331218560	0.6613625880626283	T T T
0.2993245753140364	0.4179603405062144	0.4802403878991833	T T T
0.3996155518128769	0.2904563450734391	0.3403264511898900	T T T
0.9484483081341580	0.5895670355732227	0.4816435718647649	T T T
0.8045747255123776	0.9725099105879736	0.4301479880152393	T T T
0.6606414244083361	0.9219031670002230	0.5393021834772770	T T T
0.9580370875499116	0.4022522158791599	0.4092984318126838	T T T
0.7080276996916681	0.6369302595770471	0.5073019406503849	T T T
0.8630724209666616	0.0797157057571963	0.5090861422814825	T T T
0.1959570578646224	0.9154975334534448	0.5736864275455519	T T T
0.9317648663148927	0.6562209130445597	0.3636987191676764	T T T
0.5022391609619520	0.5546073344198416	0.3765194714428952	T T T
0.2705769984805357	0.0193025373450172	0.4407194811717310	T T T
0.6851566561004296	0.7854864488564348	0.3719972542234520	T T T
0.5203433388220738	0.9952735523178620	0.3372187829112240	T T T
0.6305206655314658	0.2193724678430498	0.6258469243247470	T T T
0.5103259466921989	0.2876323583461583	0.6321204433508998	T T T
0.7454869448431514	0.3060491722222309	0.6270514893402802	T T T
0.5033481219510354	0.4442690061959439	0.6402976618695539	T T T
0.9780800185704314	0.3146473908185058	0.6189390601884033	T T T
0.3802714643765436	0.5152277300635097	0.6448338058166512	T T T
0.7394237877371828	0.4617904623106649	0.6356341654232546	T T T
0.6190768486025122	0.5304944564853841	0.6422090164745831	T T T
0.2782476631285586	0.5745983013482506	0.6477815938591764	T T T

The molecular transport final state of without surfactant

1.00000000000000		
10.2424999999999997	0.0000000000000000	0.0000000000000000
0.0000000000000000	8.8702000000000005	0.0000000000000000

0.0000000000000000 0.0000000000000000 30.6609000000000016
Cu H O C N
64 103 49 8 1
Selective dynamics
Direct
0.5012803437249952 0.5013096200476225 0.2637575404421536 T T T
0.1226617750208221 0.7576692976485444 0.2623160892495052 T T T
0.6273101513067602 0.2538801861171779 0.2640965191191514 T T T
0.8741095397334082 0.7533209022622253 0.2644103327035678 T T T
0.7539771303331756 0.0018665929180311 0.2647724611636645 T T T
0.5064608460459069 0.9990094545783190 0.2677193536114282 T T T
0.8755092470437613 0.2516751129248985 0.2652015510310839 T T T
0.7510058344355062 0.5017208046967042 0.2653749346161339 T T T
0.6293666248078064 0.7498056709737223 0.2644178576562945 T T T
0.1261590364733018 0.2475755006184593 0.2623529912410876 T T T
0.3759130368022450 0.2508402234087757 0.2636622305743368 T T T
0.2546337287800751 0.5021764563575842 0.2627107465819624 T T T
0.9998185369479630 0.5014077443480539 0.2696173987020919 T T T
0.2504826130247845 0.0001382207606998 0.2693403842120610 T T T
0.3755794564695820 0.7514557852319115 0.2620626933212604 T T T
0.9977075218634740 0.0025718508241035 0.2644695124296021 T T T
0.7511216109472770 0.3384414541346891 0.1981109352122471 T T T
0.5006052310304789 0.3353602503326237 0.1978715965724428 T T T
0.8781712623595632 0.5861082767783864 0.1989431301284766 T T T
0.2502853963925223 0.8369735351039916 0.1976449216928828 T T T
0.9997768599145350 0.8362086762423224 0.1972720683831139 T T T
0.0014429851471027 0.3395090930099924 0.1988045180622127 T T T
0.6250538479864729 0.0866839320697612 0.1985805858434967 T T T
0.7508909225692189 0.8379836591251847 0.1974789124339199 T T T
0.5019716000999221 0.8363813962681380 0.1982152752772365 T T T
0.1251259130726864 0.5869120624485602 0.1970683218055303 T T T
0.6263176014145957 0.5872397148631391 0.1978344269968960 T T T
0.1281959727209818 0.0830217077963518 0.1984888445331123 T T T
0.3753104959252155 0.5863807837708612 0.1966260960484836 T T T
0.2516869878374255 0.3375858170708380 0.1969414025232067 T T T
0.8759334938687136 0.0886971115753130 0.1975381099391287 T T T
0.3756227240354296 0.0818552708054809 0.1994878724912247 T T T
0.8751817693360231 0.4180782983781430 0.1328226019757181 T T T
0.3752659248461657 0.9181391406066962 0.1325839584276117 T T T
0.9995779323575080 0.6692280465217298 0.1319525005076833 T T T
0.7499155941841817 0.1689358687073851 0.1328650226945713 T T T
0.3759448336304024 0.4181127606521061 0.1314401740374375 T T T
0.6252913390095008 0.9192284380647284 0.1324830047314438 T T T
0.2498592033645924 0.6692867511119677 0.1316814041358188 T T T

0.1253134856816646	0.4186743368848660	0.1318477890865189	T	T	T
0.0008246053872814	0.1694315749479396	0.1329871769728538	T	T	T
0.7507408336280775	0.6674094564959244	0.1329987574903532	T	T	T
0.1251726271792192	0.9187919279569116	0.1320548803963022	T	T	T
0.5008169618988855	0.6689666903551768	0.1318761265371681	T	T	T
0.6255056881287641	0.4183050255232031	0.1323274275672460	T	T	T
0.2515903053428555	0.1680203568681369	0.1328683893956048	T	T	T
0.8747504817740568	0.9193942989552596	0.1324017822942120	T	T	T
0.5005534323588403	0.1689046031055057	0.1326715552191848	T	T	T
0.2500000000000000	0.0000000000000000	0.0652299999999997	F	F	F
0.6250000000000000	0.7500000000000000	0.0652299999999997	F	F	F
0.8750000000000000	0.2500000000000000	0.0652299999999997	F	F	F
0.0000000000000000	0.5000000000000000	0.0652299999999997	F	F	F
0.8750000000000000	0.7500000000000000	0.0652299999999997	F	F	F
0.1250000000000000	0.7500000000000000	0.0652299999999997	F	F	F
0.3750000000000000	0.2500000000000000	0.0652299999999997	F	F	F
0.6250000000000000	0.2500000000000000	0.0652299999999997	F	F	F
0.7500000000000000	0.5000000000000000	0.0652299999999997	F	F	F
0.1250000000000000	0.2500000000000000	0.0652299999999997	F	F	F
0.2500000000000000	0.5000000000000000	0.0652299999999997	F	F	F
0.5000000000000000	0.0000000000000000	0.0652299999999997	F	F	F
0.0000000000000000	0.0000000000000000	0.0652299999999997	F	F	F
0.7500000000000000	0.0000000000000000	0.0652299999999997	F	F	F
0.5000000000000000	0.5000000000000000	0.0652299999999997	F	F	F
0.3750000000000000	0.7500000000000000	0.0652299999999997	F	F	F
0.1869281008962752	0.8980380682356754	0.7733968912072837	T	T	T
0.2743863280830225	0.1434097318687567	0.9902518105853868	T	T	T
0.5982433830841216	0.5460515399601884	0.9838614299023154	T	T	T
0.5926506694723581	0.0366443267602979	0.9415938219104224	T	T	T
0.4457187371429773	0.4940750735878892	0.9824587964450692	T	T	T
0.0204797855122124	0.9277812400180464	0.8132592021326098	T	T	T
0.2635923815953630	0.0132992482613584	0.7445462356051643	T	T	T
0.9116403175844514	0.4402475367390004	0.8632275331144716	T	T	T
0.7743769477365582	0.6712480765635095	0.9921873344161416	T	T	T
0.7177157995981268	0.7352854683695150	0.9474023737073292	T	T	T
0.1277740801840490	0.0056803709364509	0.8430050863184498	T	T	T
0.8825719754357271	0.5583113326011571	0.8254154821744092	T	T	T
0.3934988033060393	0.1547386431994101	0.9574974187551286	T	T	T
0.5968237649937576	0.1713197855472144	0.9760859303968084	T	T	T
0.1963385697030267	0.2608679654654905	0.8700159955962873	T	T	T
0.8220483879937767	0.4938384147957535	0.9335126218713378	T	T	T
0.1984783410285598	0.5476185112884815	0.8673920797760684	T	T	T
0.2192573209086667	0.1455645423272201	0.9087496825835100	T	T	T
0.5610166167487887	0.9071967397638911	0.7175058359317172	T	T	T

0.6409573877123916	0.0539990761080526	0.7249174745229908	T	T	T
0.8928317863296802	0.3355350291955180	0.9372037886591168	T	T	T
0.2874124314151853	0.3722343803863724	0.9603569761428796	T	T	T
0.7823278526966101	0.2483352847093734	0.7611337965529665	T	T	T
0.1094916791006001	0.4522036293829286	0.8373418789984105	T	T	T
0.4795079321706899	0.8728260882705068	0.8418294112056607	T	T	T
0.0209974740773382	0.1945925875844191	0.9847686559270272	T	T	T
0.3153399364149008	0.5106857296848524	0.9292556035793034	T	T	T
0.4915255074047106	0.6894393635477586	0.8458236931377746	T	T	T
0.6784394456064728	0.1978072990441787	0.7972917987563292	T	T	T
0.8838407523647892	0.1259550835380200	0.9858230774649966	T	T	T
0.8274945904481279	0.0259150852940220	0.7761679383067253	T	T	T
0.3606328355027525	0.4688016734129325	0.8340534643436633	T	T	T
0.9120939987059672	0.0973386797749753	0.9047970300935140	T	T	T
0.9265991207236048	0.9396569367520624	0.7459045695883240	T	T	T
0.3690948653083327	0.1377212511675470	0.7862592839604534	T	T	T
0.3380297615540850	0.0787033577455417	0.8524850917538221	T	T	T
0.1775099117884709	0.6292020325456402	0.9758475036846016	T	T	T
0.9822577615174350	0.6733569077338686	0.6884195322811628	T	T	T
0.5553762111391166	0.3400481981343166	0.8550314949736570	T	T	T
0.5021213620953178	0.7740836131587637	0.7768050028295526	T	T	T
0.4782071841604389	0.4828231866408930	0.8013955324492220	T	T	T
0.8045025403971462	0.1594429769442551	0.8737925908163258	T	T	T
0.2672970766096349	0.2502091323872514	0.7656989446839793	T	T	T
0.4873885091450769	0.1168381541602822	0.8450945291949936	T	T	T
0.0388806033100754	0.7025080319759928	0.9810186595750716	T	T	T
0.8351725774330053	0.6748984897694508	0.7026237138096897	T	T	T
0.5939421077931970	0.2301298110998667	0.8939469756086864	T	T	T
0.3792101514934457	0.8134824280128764	0.7483061244819604	T	T	T
0.6205091723040204	0.8423605921991792	0.8921796861688571	T	T	T
0.4333192879034824	0.1670461875108691	0.4127303803892891	T	T	T
0.7005029969309278	0.7241218283239667	0.8206442365064819	T	T	T
0.1611324208796096	0.8725808106957160	0.4811489259730619	T	T	T
0.2357584907643240	0.2769666270058727	0.3797901992720609	T	T	T
0.8206192496834558	0.7779883769494015	0.7929803932090426	T	T	T
0.1107011305686067	0.8926224762686439	0.5296118774645673	T	T	T
0.1601636513449167	0.7327810397389212	0.9153395726745138	T	T	T
0.7449010066054472	0.9429763853841794	0.9030623152437224	T	T	T
0.1591867903770074	0.7830808015097734	0.8652712589957544	T	T	T
0.9074998381733500	0.4452665608377193	0.7329532039736549	T	T	T
0.5579922668541850	0.2662673901129514	0.4970404813732606	T	T	T
0.9253362688938506	0.3873097719732801	0.7814145921138393	T	T	T
0.2876478069009734	0.0833512236562979	0.5697157054262763	T	T	T
0.8091704529085828	0.4319514505763599	0.4119991448476091	T	T	T

0.0996866162748515	0.1667767796448672	0.4731900398353198	T	T	T
0.5960624537366784	0.1419825500793734	0.4643149334177384	T	T	T
0.8093301096658115	0.3336378658514586	0.3605069029229462	T	T	T
0.1838628691784586	0.7044617017444460	0.6634893868614866	T	T	T
0.8624087518381836	0.2383971339693438	0.4091719704810750	T	T	T
0.3996211434477466	0.1306236015225607	0.5375207129899231	T	T	T
0.2129132669827814	0.2838371303452281	0.4745978613363998	T	T	T
0.6636254610902103	0.5415672002365909	0.3648824463773757	T	T	T
0.6179333350777813	0.5217653199818650	0.5161919106354979	T	T	T
0.1837581381304231	0.4671873191848683	0.5216152159367050	T	T	T
0.4644757996236226	0.4826618091508854	0.5138682797843908	T	T	T
0.1594379819899685	0.8369366861326044	0.6959970002187577	T	T	T
0.4645879140711628	0.6575883747626460	0.3352235005074637	T	T	T
0.0351438046825274	0.6808717320679333	0.5082778776904994	T	T	T
0.0096769766388713	0.5448925817383873	0.4779649740623517	T	T	T
0.2774938999610916	0.3323622824485900	0.5365580968765665	T	T	T
0.3494000264500500	0.9752097593074160	0.3472123464830563	T	T	T
0.2063613331449850	0.9114187509454230	0.3538231170540197	T	T	T
0.6916413006157043	0.8312917757315880	0.5371823054794663	T	T	T
0.0600037146590386	0.4339797103759503	0.4122869895408562	T	T	T
0.9363390929905366	0.9305739076546838	0.4600771103260254	T	T	T
0.6148692768882699	0.9840598594876740	0.5263248723119968	T	T	T
0.9931062356078360	0.5920782480623372	0.4035336131092621	T	T	T
0.8533589245567855	0.0610243875538901	0.4775378589679087	T	T	T
0.7563719989068947	0.6103903080140431	0.5662282979387202	T	T	T
0.7920717363853642	0.0583578079796139	0.5416841583778592	T	T	T
0.1614582410074982	0.8917960454726457	0.6118376121194103	T	T	T
0.8189998115340953	0.7627667653228709	0.3779907992249493	T	T	T
0.8422559989263845	0.6061551646630253	0.5238402446850771	T	T	T
0.2673087526287793	0.8319404209795243	0.5801103194275704	T	T	T
0.8194816259296056	0.2305043158506534	0.5346715949680019	T	T	T
0.4377220354461865	0.6919759255640366	0.4273332782586090	T	T	T
0.5325945040447804	0.1069145277955670	0.3468805126198556	T	T	T
0.3690126374405532	0.9065817168967012	0.4583811387713965	T	T	T
0.6262389750153898	0.8102217130207177	0.4171160116308985	T	T	T
0.9021424032337050	0.6876594130215996	0.3402079108895603	T	T	T
0.5928317121975610	0.9489685667900828	0.3599177956814476	T	T	T
0.2640171460928022	0.0376770979249481	0.4499034711768074	T	T	T
0.5284960912077631	0.6315269622740372	0.4639197297358526	T	T	T
0.7454373803799830	0.9277982070359864	0.4178070925464829	T	T	T
0.5318337072809634	0.4779418986651203	0.9972680108150240	T	T	T
0.2293475393707826	0.9071062247194632	0.7442865885900661	T	T	T
0.7380655298594651	0.6396677137301897	0.9638120434592145	T	T	T
0.2980938241701673	0.1789959566689583	0.9607377073084272	T	T	T

0.5565757094856448	0.1390826638405369	0.9486088202073631	T	T	T
0.1088367221963831	0.9129438846700880	0.8261554558767551	T	T	T
0.9393168397810100	0.4696467061133981	0.8331819218156260	T	T	T
0.2005631166828755	0.1511967676120212	0.8770941320841007	T	T	T
0.8600867207719374	0.4107942505519645	0.9161281223882272	T	T	T
0.6133691808589149	0.9840861163408005	0.7018160337826473	T	T	T
0.1985008318868096	0.4491780222491737	0.8505418562894476	T	T	T
0.2966938065146784	0.4848174237363429	0.9595768535038868	T	T	T
0.7194957032464757	0.1653921515159174	0.7701308803828574	T	T	T
0.5413069253321064	0.7853674844052095	0.8447514595289751	T	T	T
0.9481648710017077	0.1682378015748411	0.9653294153532758	T	T	T
0.8935910875606904	0.9419836708886168	0.7757160754219123	T	T	T
0.4563417159535091	0.4892514834169142	0.8323270411633197	T	T	T
0.8535088161228827	0.0679548494434602	0.8809317673774444	T	T	T
0.3347110777919410	0.1774873446307702	0.7580424587438005	T	T	T
0.4154725030038783	0.0490581636793310	0.8344520708813165	T	T	T
0.1288858793406399	0.7253820067250093	0.9728999291667336	T	T	T
0.9085309871874162	0.6076965231804732	0.6972596199303144	T	T	T
0.5997735119073337	0.2429360834054905	0.8619276305716179	T	T	T
0.4676234345471837	0.7663030443257312	0.7471141744924853	T	T	T
0.7778246213902803	0.6863344608539992	0.8050094467424560	T	T	T
0.0824290637242392	0.8611653120492004	0.5002058388765934	T	T	T
0.6718400021453584	0.8847413486093821	0.9170537834488192	T	T	T
0.2041538768740753	0.7174759680551644	0.8868914135768965	T	T	T
0.6660739944594191	0.2641987985691231	0.4052758234076402	T	T	T
0.9094840331066140	0.3523841436703391	0.7510475147000787	T	T	T
0.1733380886930088	0.2034458557511709	0.4560570459779943	T	T	T
0.5531442773606197	0.1554057986611475	0.4924319856437714	T	T	T
0.3425467180344559	0.1696796300692721	0.5605116584134638	T	T	T
0.5479865247856293	0.4731560292610893	0.4982921685878478	T	T	T
0.1239615993980078	0.7877102051763968	0.6691896885383681	T	T	T
0.2660259829358184	0.4150912195139152	0.5148164210627419	T	T	T
0.2562847185808066	0.9910993835866880	0.3396395551683800	T	T	T
0.0024047666828733	0.5742864854396487	0.5094142690060103	T	T	T
0.8674051208391721	0.0001109328659883	0.4505002933096506	T	T	T
0.6653339437810506	0.9307347250428870	0.5488343172297253	T	T	T
0.0487528379221162	0.5343823471273742	0.4244085596806255	T	T	T
0.7517482613725378	0.6293391853530735	0.5349139099549624	T	T	T
0.8588020622827894	0.1314456550001009	0.5308146692352638	T	T	T
0.2000061041121422	0.9093603366282792	0.5827389375097149	T	T	T
0.9032857922836413	0.7116328801687611	0.3714382203492115	T	T	T
0.5075577478366905	0.7241825870402239	0.4467801087701634	T	T	T
0.2836192885872790	0.9284775494724512	0.4456724578116097	T	T	T
0.6865419261618357	0.8692923484173816	0.3978390231580068	T	T	T

0.5235888736607157   0.9988775241925600   0.3411610163275806 T T T
0.4397872410305663   0.2735176810165396   0.3952381437494626 T T T
0.3282435011133760   0.3365916173490781   0.3773327445970902 T T T
0.5622217935272443   0.3426722826015435   0.3896235870174725 T T T
0.3364240596371127   0.4747424654665783   0.3547868235121522 T T T
0.7948731388590382   0.3223664624472529   0.3960346508516218 T T T
0.2241377912526196   0.5194119128478274   0.3300703823216606 T T T
0.5708902415582318   0.4836913928508301   0.3687812070532507 T T T
0.4582952203517917   0.5481653957855268   0.3515375918430806 T T T
0.1071061402661511   0.5257974519917603   0.3238533133680172 T T T

The molecular transport initial state of with surfactant
1.00000000000000
10.2424999999999997   0.0000000000000000   0.0000000000000000
0.0000000000000000   8.8702000000000005   0.0000000000000000
0.0000000000000000   0.0000000000000000   30.6609000000000016
Cu   H   O   C   N
64   109   43   15   2
Selective dynamics
Direct
0.5000966203037717   0.5000108616223322   0.2669260636973396 T T T
0.1256600472933352   0.7486150501020390   0.2664857245134071 T T T
0.6248346579481794   0.2503804010094315   0.2672982878178102 T T T
0.8732612922013516   0.7493907748860967   0.2652251068462380 T T T
0.7481310267164206   0.0001119771424011   0.2666864823916327 T T T
0.4999697776070127   0.0002308985286852   0.2672558316886850 T T T
0.8743187572404567   0.2517705174691906   0.2671228618465343 T T T
0.7499215468562400   0.5004404313517923   0.2674812723748758 T T T
0.6245914054692856   0.7500789642441558   0.2668646048952444 T T T
0.1258630149303026   0.2513168778958480   0.2658182254243899 T T T
0.3750893455495276   0.2502468807163478   0.2669586184407060 T T T
0.2500421159437374   0.5000142940543807   0.2672758228547883 T T T
0.9999070509779030   0.5004376448115062   0.2672354280890462 T T T
0.2512740550278505   0.0000559339394482   0.2659118628125853 T T T
0.3748220463738029   0.7503172347216576   0.2658481979160423 T T T
0.9999183806331120   0.0007510750336290   0.2707171647480098 T T T
0.7497006372380427   0.3331366969564484   0.1992563918336472 T T T
0.5002474534571901   0.3332728370595956   0.1992225280005896 T T T
0.8745253985908079   0.5823156171741147   0.1990220856671942 T T T
0.2495915760529319   0.8328651986690483   0.1989198435817415 T T T
0.0001576190217060   0.8346828548632120   0.1997890965126730 T T T
0.9996701817480590   0.3324462165179640   0.1992183257083682 T T T
0.6249423169364687   0.0834777301959438   0.1991045456812686 T T T
0.7496417583263127   0.8334232026220962   0.1989670678920746 T T T

0.5000513999315653	0.8327656529466551	0.1989805173118301	T	T	T
0.1251029094408940	0.5825792681401407	0.1991021404581944	T	T	T
0.6249782948116596	0.5828479103402927	0.1990729358425648	T	T	T
0.1240674437658487	0.0824447502504010	0.1996478947455386	T	T	T
0.3750887459523744	0.5825757991996225	0.1989804573535082	T	T	T
0.2503758683284567	0.3334294134776299	0.1989178372348788	T	T	T
0.8757440592210687	0.0825565047820877	0.2001597146732276	T	T	T
0.3751365988067377	0.0833615178280093	0.1990117323837656	T	T	T
0.8748717780161896	0.4173185068399042	0.1331750450559914	T	T	T
0.3747914543123075	0.9173014179118322	0.1331194159255580	T	T	T
0.9999655877034550	0.6685964900200868	0.1334506505452955	T	T	T
0.7506898019383705	0.1667652066197636	0.1334264649141634	T	T	T
0.3751251759035070	0.4172856754815214	0.1330262259156049	T	T	T
0.6251013298497594	0.9172666278936824	0.1331797523453473	T	T	T
0.2499602977775743	0.6670154472736890	0.1331270551864513	T	T	T
0.1249832610440248	0.4170410826977319	0.1331342614522823	T	T	T
0.9998863570966821	0.1673104792689283	0.1332877617541579	T	T	T
0.7500534334709197	0.6674966775941568	0.1331034785832170	T	T	T
0.1248743084389257	0.9175332703187332	0.1332764971598578	T	T	T
0.5000003643470636	0.6675170390577695	0.1329813152516016	T	T	T
0.6251055325792413	0.4170300211719907	0.1330767603749498	T	T	T
0.2494899792567496	0.1666501064879964	0.1332592129630567	T	T	T
0.8751115082282442	0.9177539217976224	0.1332822417583764	T	T	T
0.5000164984283660	0.1678508630812322	0.1332288593138604	T	T	T
0.2500000000000000	0.0000000000000000	0.0652299999999997	F	F	F
0.6250000000000000	0.7500000000000000	0.0652299999999997	F	F	F
0.8750000000000000	0.2500000000000000	0.0652299999999997	F	F	F
0.0000000000000000	0.5000000000000000	0.0652299999999997	F	F	F
0.8750000000000000	0.7500000000000000	0.0652299999999997	F	F	F
0.1250000000000000	0.7500000000000000	0.0652299999999997	F	F	F
0.3750000000000000	0.2500000000000000	0.0652299999999997	F	F	F
0.6250000000000000	0.2500000000000000	0.0652299999999997	F	F	F
0.7500000000000000	0.5000000000000000	0.0652299999999997	F	F	F
0.1250000000000000	0.2500000000000000	0.0652299999999997	F	F	F
0.2500000000000000	0.5000000000000000	0.0652299999999997	F	F	F
0.5000000000000000	0.0000000000000000	0.0652299999999997	F	F	F
0.0000000000000000	0.0000000000000000	0.0652299999999997	F	F	F
0.7500000000000000	0.0000000000000000	0.0652299999999997	F	F	F
0.5000000000000000	0.5000000000000000	0.0652299999999997	F	F	F
0.3750000000000000	0.7500000000000000	0.0652299999999997	F	F	F
0.4591192130967145	0.2914748314096883	0.9043479174547268	T	T	T
0.5314935055217553	0.0013556640267555	0.9943193466612320	T	T	T
0.2012107162040938	0.7291859438516026	0.8991033595671669	T	T	T
0.0544185788887583	0.3444911096465788	0.9216011847743336	T	T	T

0.1697001997819118	0.8287617127090262	0.8573197850912814	T	T	T
0.6044291783439133	0.4487283120744200	0.9153917165251684	T	T	T
0.6049552517086826	0.2403163249594412	0.8948622155861727	T	T	T
0.5139574596604571	0.6454260773757416	0.9464276559195004	T	T	T
0.3269059458641326	0.2807485631278926	0.9703819528001516	T	T	T
0.3956418467811286	0.1308223233859198	0.9534620385050798	T	T	T
0.6983792272667835	0.4893317261420199	0.9551537201106776	T	T	T
0.3582605830659556	0.6719365460726645	0.9531005496392267	T	T	T
0.4787979263812907	0.8874852132731269	0.9580613986346432	T	T	T
0.9450228216523436	0.4671560024601611	0.9269916552248380	T	T	T
0.4427819606051529	0.7886257536852986	0.8048328403514524	T	T	T
0.1146716729037607	0.8097672524830128	0.7430300783589567	T	T	T
0.6106596089392073	0.7316358948625099	0.8531296008601088	T	T	T
0.3771365756895279	0.7185257643808970	0.8465247399013193	T	T	T
0.9958844101978170	0.0462038640001212	0.9729666167419032	T	T	T
0.0334125459265147	0.8810938973077483	0.9841298849476964	T	T	T
0.1736377709330831	0.6441519955771212	0.7374288850850942	T	T	T
0.0242712739992282	0.4561248852272513	0.8299463977405052	T	T	T
0.1264316773644069	0.5495322619544193	0.9537903788260376	T	T	T
0.6985372039020752	0.6452962934666997	0.8872221702097983	T	T	T
0.8065198352911047	0.8511477939126257	0.8798353848827150	T	T	T
0.6051768920008626	0.8088533826231862	0.6952154188728830	T	T	T
0.1447039051515217	0.5696287228207565	0.8420554649838849	T	T	T
0.9151534582391164	0.9128871829940348	0.9147322018301332	T	T	T
0.1814362291308981	0.6597779806771040	0.9900225052126672	T	T	T
0.5041641959160155	0.9057282186945173	0.7224507612688434	T	T	T
0.4200896409223253	0.9552384394976664	0.6629849981296700	T	T	T
0.0000348086010734	0.9661455181992180	0.8542419329537220	T	T	T
0.7651431974100992	0.0681695939109237	0.9061592367592683	T	T	T
0.3052335842001490	0.9055694609268474	0.6323230470793264	T	T	T
0.8250958151625852	0.0498407459432014	0.7309715183122920	T	T	T
0.2522487470443098	0.2262243332958788	0.7792136085712356	T	T	T
0.3201683450151454	0.8467317090527942	0.7526130816294846	T	T	T
0.4788538252145437	0.5243451795398094	0.8083623302256250	T	T	T
0.0607965054937529	0.0373559054116255	0.7336909723197780	T	T	T
0.2261829543850502	0.2271311748945945	0.9039565893384018	T	T	T
0.1177996918889473	0.0799825600892318	0.8546237470802860	T	T	T
0.6413232193559546	0.0733337973584253	0.9370758929311308	T	T	T
0.6877790077625512	0.0306901555681859	0.7071199040258428	T	T	T
0.3965156368576421	0.2831113032794035	0.7825979595301078	T	T	T
0.5174239884305557	0.3759526537892126	0.8336318792950103	T	T	T
0.3752117418955325	0.0092819789307721	0.7687988450456362	T	T	T
0.0432868809426653	0.9982348016752530	0.7847193346926971	T	T	T
0.1614391421616989	0.3246053699770314	0.8645318536254365	T	T	T

0.6972945326333206	0.5574776972606618	0.7137855550526059	T	T	T
0.0109413951275207	0.1819776060735266	0.6608782155757233	T	T	T
0.2224043585851349	0.0832942058992712	0.6786767117164139	T	T	T
0.3688522506876160	0.6189716306396360	0.6799038654621049	T	T	T
0.1419910947996930	0.6645618084295974	0.6555052318952883	T	T	T
0.8600474192012516	0.1581950966280511	0.6661809974849283	T	T	T
0.1865679410390753	0.2301280330331698	0.7058911793096260	T	T	T
0.8186199241998208	0.3926987687751607	0.7985559885201988	T	T	T
0.7458089483984248	0.7075880496375540	0.7337195844390347	T	T	T
0.8561165468439798	0.2976765379879740	0.8398865795951043	T	T	T
0.8553201225519111	0.0288484861203553	0.5339364598356843	T	T	T
0.2246309278129918	0.9425212214771912	0.5178895253586285	T	T	T
0.6324186512372628	0.3878903519735963	0.7698375481626379	T	T	T
0.8146867761588710	0.9898288915177580	0.4856963281558909	T	T	T
0.7786848646211689	0.7562406217983756	0.8087240533608863	T	T	T
0.5866282864678954	0.2202096200860457	0.6265245008465707	T	T	T
0.3227879393063411	0.7845006481970370	0.5049626201518533	T	T	T
0.7145983341989202	0.3524684040312238	0.6381761618934548	T	T	T
0.7200294411911343	0.2808435362361557	0.7393978463181042	T	T	T
0.1357014087989143	0.9383049162864316	0.4397687271272029	T	T	T
0.1497459456139955	0.7687120242025326	0.5045852673971675	T	T	T
0.0496972915155010	0.2330509810683156	0.3546273725842549	T	T	T
0.2375810496665200	0.7837076402550521	0.4282893085955040	T	T	T
0.6256355768670909	0.3664898025021288	0.5880830139546572	T	T	T
0.4160435304729170	0.2811996830552130	0.5793745900106522	T	T	T
0.8735390322841259	0.8457885380693531	0.7764034784579323	T	T	T
0.1889224478371258	0.3269504366129841	0.5550169542089997	T	T	T
0.9640535730885030	0.8787527090891475	0.3523176505312277	T	T	T
0.0985357814175613	0.9647914375937860	0.3524141122531022	T	T	T
0.1233907772482873	0.3799421245245561	0.3454151731698180	T	T	T
0.4340262562304423	0.9486130512185960	0.4484485404171002	T	T	T
0.2982332803286205	0.1570297046829168	0.4849921909763754	T	T	T
0.1288748969480177	0.4557815172790624	0.7742161674365085	T	T	T
0.0388518249884663	0.4476238231021984	0.7321931075195549	T	T	T
0.3529444637613430	0.9887162769384304	0.3989509043081595	T	T	T
0.2297238067032409	0.1987376325280130	0.4328023661355425	T	T	T
0.9426659445433682	0.1599429892075234	0.5931639489542520	T	T	T
0.5193377478188698	0.2115481451845921	0.5011587531973026	T	T	T
0.5828974424957957	0.1171117068401983	0.4542282124061265	T	T	T
0.0445241188000264	0.0820389922112627	0.5625314403562206	T	T	T
0.5609418604442159	0.9818711465064818	0.5824440715428050	T	T	T
0.8626963562493108	0.3407038212995673	0.4948622822146402	T	T	T
0.3548415524012475	0.4102497777250177	0.4915068272982857	T	T	T
0.8091062184790725	0.7475649518778851	0.3782663475104015	T	T	T

0.3666027356533197	0.2989683619550706	0.3755076525858848	T	T	T
0.2932308020055079	0.4506460808365909	0.4377569755935519	T	T	T
0.5262037178954113	0.3682827001427633	0.3887839921381449	T	T	T
0.6175757069594893	0.3127632535174935	0.4621699336571765	T	T	T
0.6378347152636890	0.9948005048019460	0.5387282282938145	T	T	T
0.8797248786358969	0.6563668354455985	0.3400554396848450	T	T	T
0.9347533902560450	0.4134567535055979	0.4554840753258686	T	T	T
0.5322973974430441	0.7557191575635342	0.3795563399917388	T	T	T
0.4580088947445368	0.4994638016752900	0.4519060108497070	T	T	T
0.4995859233040653	0.1696949581047048	0.3826014832080274	T	T	T
0.6843434047333796	0.7558122066864326	0.4334884632991836	T	T	T
0.6863322229445462	0.6610767080896541	0.5018237266401920	T	T	T
0.8425059118650715	0.1796876933147786	0.4347793043028609	T	T	T
0.7186714825989613	0.9199996051471204	0.4144696077127285	T	T	T
0.7445716293486778	0.5405218755296117	0.4696427719647939	T	T	T
0.4690171594663100	0.6800841555651010	0.3388573512224791	T	T	T
0.9045528555079244	0.0581308721250439	0.4034024910089529	T	T	T
0.2096998147638527	0.7312859847969964	0.8671424872651214	T	T	T
0.5362637915273788	0.3181246906127683	0.8863452482171208	T	T	T
0.3454266716085913	0.2228126885858521	0.9439926024843520	T	T	T
0.5064667604496277	0.9969517801224012	0.9632501646627596	T	T	T
0.0159654294097604	0.4181190425414503	0.9426725405228954	T	T	T
0.6455281353744212	0.5347661224485390	0.9323054282363112	T	T	T
0.4433684655609168	0.7218437555212182	0.9485263823032604	T	T	T
0.4577468497098577	0.7126236810195926	0.8286390895016406	T	T	T
0.1933977761050237	0.7534257772569989	0.7340708544277286	T	T	T
0.0191300559425425	0.9497838018416356	0.9595839785970084	T	T	T
0.7028083266395732	0.7214000982927075	0.8635769415466464	T	T	T
0.1217988706431457	0.4697459867294358	0.8291189830730124	T	T	T
0.1886747604518594	0.6361849799994850	0.9587516634924780	T	T	T
0.8705883672653298	0.9339401634487322	0.8865637561482631	T	T	T
0.5602859954053663	0.9089730966137241	0.6953493878508091	T	T	T
0.3404004183772787	0.9922976674198102	0.6477986386479677	T	T	T
0.0855050006334263	0.9871747951406070	0.8393895128705423	T	T	T
0.7053763397795629	0.1386900931836673	0.9216697806880836	T	T	T
0.7620157595035584	0.1025669116946515	0.7123089633080628	T	T	T
0.3410500225497263	0.1993345981840082	0.7711525490108154	T	T	T
0.4001178628029370	0.9020073040240388	0.7633689846248530	T	T	T
0.4999733984033480	0.4158106629793821	0.8036460614103270	T	T	T
0.0088789499506292	0.9763769274021520	0.7548395263653316	T	T	T
0.1481223187344916	0.2378869883745625	0.8848653252618593	T	T	T
0.9263248131143632	0.2000489461778938	0.6459060130169669	T	T	T
0.1489510597819802	0.1381853211007386	0.6934739044883629	T	T	T
0.7161680350611416	0.6625828895220689	0.7055493206172723	T	T	T

0.8699728294979313 0.3953877557105491 0.8263327044129708 T T T
0.5323307855127544 0.4390554594318489 0.6452233700800215 T T T
0.7936945866950749 0.9660432856361520 0.5161683596498047 T T T
0.7161344266879529 0.3820517806112027 0.7531276884213788 T T T
0.7856188916369987 0.8038953604388661 0.7797622532998505 T T T
0.0055022776239079 0.9818122343114850 0.3472851676059130 T T T
0.1030801381827124 0.3096500302449901 0.3691642578453597 T T T
0.1297042706457758 0.4507901653316307 0.7415799995900738 T T T
0.9627516080791744 0.1377043811188739 0.5616272507529688 T T T
0.5502987765914005 0.0104832565289268 0.5520700261663182 T T T
0.8516687300723456 0.3694312958594471 0.4643459994283672 T T T
0.8967474574324432 0.7187728827375437 0.3659606874082336 T T T
0.4548744012529346 0.6979260098825352 0.3700999738452491 T T T
0.6798890730398675 0.6228354332643860 0.4721330033481262 T T T
0.8409512331442226 0.0713473805859888 0.4270368776655243 T T T
0.6824246561184132 0.8207083799655706 0.4065542304286869 T T T
0.3289810074731538 0.5573290713764651 0.6521898823044086 T T T
0.2022043834842625 0.5824392712088596 0.6384078149603258 T T T
0.4081493461929935 0.4515199212781115 0.6303684496598082 T T T
0.1512106179408599 0.5012753953152282 0.6026450252142935 T T T
0.0227388379116993 0.5312256399949226 0.5875620759949723 T T T
0.6192491020648498 0.3374199115700499 0.6228280889254498 T T T
0.2317715993534852 0.8434588672101579 0.4965238234511209 T T T
0.3571269168899239 0.3669417899650538 0.5954608541194769 T T T
0.2294030132375098 0.3922882334894202 0.5818702572498000 T T T
0.2301879377434536 0.8864237223536819 0.4481617086856667 T T T
0.3424127195584470 0.9920189349088518 0.4345714948574805 T T T
0.3156409901660874 0.1527571743682594 0.4497005851697665 T T T
0.5449401205113085 0.2238343754202586 0.4666460842438169 T T T
0.3790873163143303 0.4179726616619658 0.4567610854702306 T T T
0.4567073823975369 0.2759812701586339 0.3935619750891892 T T T
0.9177349042548866 0.5597828234905202 0.5748266586037811 T T T
0.4249300956717025 0.2659075592896897 0.4414058016920328 T T T
The molecular transport final state of with surfactant
1.00000000000000
10.2424999999999997 0.0000000000000000 0.0000000000000000
0.0000000000000000 8.8702000000000005 0.0000000000000000
0.0000000000000000 0.0000000000000000 30.6609000000000016
Cu H O C N
64 109 43 15 2
Selective dynamics
Direct
0.4989499150298296 0.5073808394229169 0.2647426662894332 T T T
0.1240757147829389 0.7591581270168879 0.2649651292497093 T T T

0.6241186801250669	0.2580247094188208	0.2645447486612129	T	T	T
0.8725417326111798	0.7596069143235670	0.2644217364682097	T	T	T
0.7498081032244129	0.0050979191919560	0.2655479983494501	T	T	T
0.4998723866542121	0.0061326485908793	0.2696298146341600	T	T	T
0.8716677077062909	0.2515426227988545	0.2649195486459519	T	T	T
0.7453877203070288	0.5067545117301362	0.2645020965348895	T	T	T
0.6243881155548286	0.7542197789227455	0.2656209650174798	T	T	T
0.1236768140305569	0.2509522952925153	0.2644802024011606	T	T	T
0.3728743378981079	0.2575100402688072	0.2636237395970971	T	T	T
0.2493192308989213	0.5054308737640664	0.2629324130243733	T	T	T
0.9931143957700180	0.5047094342696620	0.2675832006890199	T	T	T
0.2464973576654631	0.0051858393365541	0.2651616075817994	T	T	T
0.3725810499477732	0.7548953506958108	0.2651808756273650	T	T	T
0.9981287648802560	0.0062032559133507	0.2650824714829567	T	T	T
0.7496383943087889	0.3393070251780088	0.1979080560855315	T	T	T
0.4990345384951228	0.3381721564002138	0.1978854224510323	T	T	T
0.8746404769368706	0.5887184980663591	0.1986801818865987	T	T	T
0.2493630424903883	0.8387190610878162	0.1979559373523832	T	T	T
0.9992180485469370	0.8377445033732062	0.1978202132113902	T	T	T
0.9986796535048410	0.3397188668617588	0.1990447016042768	T	T	T
0.6231719106415520	0.0879849678125373	0.1990365304930421	T	T	T
0.7486311072069192	0.8395595270022267	0.1978718951564007	T	T	T
0.4993941266356866	0.8408551157122587	0.1994957982497964	T	T	T
0.1218391872473428	0.5887023122089543	0.1985360629697617	T	T	T
0.6246068066329076	0.5897061373630155	0.1977276485092938	T	T	T
0.1242648121650520	0.0884399956975867	0.1973946434530116	T	T	T
0.3738995266352182	0.5892600169839960	0.1977069829902015	T	T	T
0.2478983461424248	0.3387143207123716	0.1973665070260288	T	T	T
0.8735695647029862	0.0886349284914138	0.1977660603494032	T	T	T
0.3751727852891165	0.0872615056052052	0.1990691721981122	T	T	T
0.8747920610051199	0.4199125636266986	0.1325959807416589	T	T	T
0.3750191129791997	0.9196849126717588	0.1327791167557796	T	T	T
0.9991940931081280	0.6692662819492442	0.1322623864597361	T	T	T
0.7486751458561740	0.1693556457199692	0.1328154060690319	T	T	T
0.3746296556601395	0.4198357366610373	0.1324682132511699	T	T	T
0.6241172024382611	0.9196236080756052	0.1326569822415784	T	T	T
0.2491647786503636	0.6689916851693704	0.1326721433355506	T	T	T
0.1240096965385881	0.4197813552319727	0.1324520823549168	T	T	T
0.9993660687966220	0.1714771140626506	0.1326768095089678	T	T	T
0.7502065139226610	0.6692729413878299	0.1326542073027218	T	T	T
0.1251968227697569	0.9189037783871478	0.1324747433666230	T	T	T
0.4996104678072509	0.6713672216865878	0.1328510878580073	T	T	T
0.6247962161177761	0.4194101296844762	0.1325673904815195	T	T	T
0.2508970636457254	0.1692831344505319	0.1326588921949027	T	T	T

0.8741016448720194	0.9194586878910320	0.1325254208521943	T	T	T
0.4995147566938662	0.1694134885602347	0.1326755896824781	T	T	T
0.2500000000000000	0.0000000000000000	0.0652299999999997	F	F	F
0.6250000000000000	0.7500000000000000	0.0652299999999997	F	F	F
0.8750000000000000	0.2500000000000000	0.0652299999999997	F	F	F
0.0000000000000000	0.5000000000000000	0.0652299999999997	F	F	F
0.8750000000000000	0.7500000000000000	0.0652299999999997	F	F	F
0.1250000000000000	0.7500000000000000	0.0652299999999997	F	F	F
0.3750000000000000	0.2500000000000000	0.0652299999999997	F	F	F
0.6250000000000000	0.2500000000000000	0.0652299999999997	F	F	F
0.7500000000000000	0.5000000000000000	0.0652299999999997	F	F	F
0.1250000000000000	0.2500000000000000	0.0652299999999997	F	F	F
0.2500000000000000	0.5000000000000000	0.0652299999999997	F	F	F
0.5000000000000000	0.0000000000000000	0.0652299999999997	F	F	F
0.0000000000000000	0.0000000000000000	0.0652299999999997	F	F	F
0.7500000000000000	0.0000000000000000	0.0652299999999997	F	F	F
0.5000000000000000	0.5000000000000000	0.0652299999999997	F	F	F
0.3750000000000000	0.7500000000000000	0.0652299999999997	F	F	F
0.4581519033486869	0.2973708573808916	0.9093383714834544	T	T	T
0.5169550531179840	0.9802532264169800	0.9944766140149504	T	T	T
0.1995818404572808	0.7330425213573907	0.8988840068843353	T	T	T
0.0550196592580786	0.3506972728651683	0.9240904796066294	T	T	T
0.1646445075048755	0.8341556543270402	0.8580042983081562	T	T	T
0.6066558577162499	0.4477881814203186	0.9250114759287954	T	T	T
0.6016027726882665	0.2415563459235454	0.8982067032839378	T	T	T
0.5058325617149489	0.6489850295425253	0.9447344737297938	T	T	T
0.3135629079771323	0.2698077548392399	0.9739041453395664	T	T	T
0.3886079018537216	0.1281917184555515	0.9548229531056678	T	T	T
0.6805613005716453	0.4976420235197082	0.9688000433491448	T	T	T
0.3514282241163824	0.6787376655926699	0.9493782303783154	T	T	T
0.4701864549598078	0.8819835655158157	0.9538904014862560	T	T	T
0.9379597273020596	0.4611948388350103	0.9331295518876396	T	T	T
0.4497158165124172	0.7905820559860395	0.8079569816353964	T	T	T
0.1062332324769462	0.8117900407220165	0.7550626026885018	T	T	T
0.6102279079787329	0.7237239442486447	0.8588793223408795	T	T	T
0.3805326941225103	0.7206025118858160	0.8488303355933464	T	T	T
0.9875649105553700	0.0234231748882460	0.9821959341725416	T	T	T
0.0539368840590763	0.8654641451409266	0.9780518564292956	T	T	T
0.1750761222595047	0.6568068215158265	0.7652516023058334	T	T	T
0.0275574605550119	0.4583599945630842	0.8327594478373976	T	T	T
0.1202504644163480	0.5637254344953885	0.9560997898946012	T	T	T
0.6984006395148814	0.6437326884072112	0.8945256140667449	T	T	T
0.8076040124621160	0.8480811648210811	0.8840025053826718	T	T	T
0.6105450712692208	0.7900568100510230	0.7002831936946409	T	T	T

0.1469618044649583	0.5741542574547974	0.8433160311554760	T	T	T
0.9162716666860292	0.9167398768209056	0.9182416254048832	T	T	T
0.1923272562798923	0.6536048284371191	0.9937340801267124	T	T	T
0.5098242886142453	0.9006471458613621	0.7236365294161106	T	T	T
0.4139829740899631	0.9303939918430740	0.6661964229854294	T	T	T
0.9931756169560260	0.9694532145782450	0.8583056759802404	T	T	T
0.7618854616095326	0.0623852946091671	0.9084331201660920	T	T	T
0.2861584285450549	0.8535079582879203	0.6505084873695784	T	T	T
0.8340285673728416	0.0448841725805856	0.7370254709905844	T	T	T
0.2504434549646120	0.2100260872375469	0.7926491564762425	T	T	T
0.3222189533635528	0.8580487866538783	0.7572314546217926	T	T	T
0.4760620496266853	0.5207734346426734	0.8129102844186691	T	T	T
0.0536532406616164	0.0216715054806251	0.7383827158706738	T	T	T
0.2265128948387125	0.2320066097327872	0.9056950916628872	T	T	T
0.1108319826594821	0.0858176015980522	0.8580010361381289	T	T	T
0.6360679683146652	0.0670071092978109	0.9390263022392400	T	T	T
0.6980517506791802	0.0190953009454466	0.7126929368800092	T	T	T
0.3875587790794844	0.2854835789596151	0.7879855917118704	T	T	T
0.5101457234238657	0.3735037645433560	0.8395218156821288	T	T	T
0.3817917880356927	0.0183333452120055	0.7709806069079876	T	T	T
0.0333368194751219	0.9968713890491400	0.7904816835792762	T	T	T
0.1644711705513069	0.3300894183278068	0.8658638923978498	T	T	T
0.7022183731731017	0.5376855000417020	0.7227226176194202	T	T	T
0.0126960955263214	0.1721727511975810	0.6663546407660723	T	T	T
0.2186375398066752	0.0562657380533675	0.6856729383413475	T	T	T
0.4667541505909307	0.7494107564458853	0.3946920902784640	T	T	T
0.2265746124808449	0.7486768297265519	0.3816599758117740	T	T	T
0.8610913159657334	0.1658219263182891	0.6719376286228126	T	T	T
0.1722118298816603	0.1999003367176761	0.7146501407402214	T	T	T
0.8207544438465781	0.3852654027228420	0.8050930289166112	T	T	T
0.7474339081751934	0.6923903512148855	0.7397833703447860	T	T	T
0.8647547246598739	0.2957027442611259	0.8467890713284107	T	T	T
0.8800682783902688	0.0392021089329446	0.5565246399306626	T	T	T
0.1842695888571520	0.8634116264820285	0.5534738903976748	T	T	T
0.6311642040568520	0.3769177532751988	0.7774655165509734	T	T	T
0.7974563634970567	0.9650667390061360	0.5201169170362935	T	T	T
0.7792086342042398	0.7390244513160811	0.8148854035664417	T	T	T
0.6689593166736240	0.3036687045395304	0.3936969691936329	T	T	T
0.2836011075086594	0.7110599499135615	0.5358961079293920	T	T	T
0.8025808932869427	0.4334363000192353	0.3846967740783810	T	T	T
0.7208976354680688	0.2689466237303131	0.7475171247600458	T	T	T
0.1019816664059658	0.8934168639504649	0.4753782830633098	T	T	T
0.1104412498082645	0.6966820931169799	0.5338312981545283	T	T	T
0.0559939236539985	0.1524096508315210	0.4030867339783296	T	T	T

0.2000424866971445	0.7408445896228730	0.4593567693091699	T	T	T
0.7062004907549326	0.3699888409757959	0.3395352522844801	T	T	T
0.4894697367916739	0.2849938086070435	0.3523737821199390	T	T	T
0.8720815286403513	0.8318634568082588	0.7823889316834648	T	T	T
0.2517209305165876	0.2786952495024344	0.3439292271658278	T	T	T
0.9914965335942930	0.9284276068086754	0.3712374400315120	T	T	T
0.0788893938958246	0.0230154880025024	0.3392568857002328	T	T	T
0.1052901575184132	0.3199529094105933	0.4113148773587772	T	T	T
0.3995438425799277	0.8910018908660906	0.4853123365793627	T	T	T
0.2547013096726792	0.0855559743260720	0.5280479669916007	T	T	T
0.1073003738022106	0.4197272456739931	0.7732714615227596	T	T	T
0.0005613621721379	0.3961576387816512	0.7375278076042552	T	T	T
0.3193630948413180	0.9560231070927327	0.4376273904050865	T	T	T
0.2080451063415078	0.1556592592391745	0.4757357219830013	T	T	T
0.9365106106097766	0.2206686056147950	0.5980028676821878	T	T	T
0.4512240348177657	0.1513767500672765	0.5602217442069588	T	T	T
0.5314176228049129	0.0369502464241818	0.5203885853443392	T	T	T
0.0481220618207312	0.2136718516105927	0.5632556007368297	T	T	T
0.6312062530553944	0.9520739973133336	0.6342339419143186	T	T	T
0.9007074399361570	0.2983900799331626	0.5115654443873030	T	T	T
0.3094081836304668	0.3573104638831664	0.5371404621538517	T	T	T
0.7819084846333983	0.8240261636717140	0.3815532194318331	T	T	T
0.3935713989749431	0.2352004894693396	0.4263193779768685	T	T	T
0.2875621238193045	0.3949310701465583	0.4800633976471754	T	T	T
0.5441538708200181	0.2893765381340817	0.4517414870608095	T	T	T
0.5814970700179806	0.2286451741775201	0.5282393999706180	T	T	T
0.6985407851267306	0.9680216077845220	0.5876737847174071	T	T	T
0.8722793654806628	0.7336767151378671	0.3491803519455327	T	T	T
0.9445561423803940	0.2984757048640492	0.4616934658344704	T	T	T
0.5421619039786578	0.9650346522876174	0.3625295824006701	T	T	T
0.4414914793516220	0.4337421503412293	0.5053768794108178	T	T	T
0.5074084647732950	0.0938503182289461	0.4433574076867987	T	T	T
0.6240427995422546	0.8108161548037618	0.4291736209304280	T	T	T
0.6129161691356312	0.7039661074345056	0.5011379981987387	T	T	T
0.8098065707472666	0.1511082759086072	0.4697102733281847	T	T	T
0.6873944123853879	0.9694583477939543	0.4258629906696710	T	T	T
0.6824778963274685	0.6057292699799367	0.4663356179464320	T	T	T
0.3966145142384744	0.9756664781070532	0.3453673360305822	T	T	T
0.8657575550498722	0.9934324787530440	0.4543414988715294	T	T	T
0.2067900074931814	0.7369647914470046	0.8669972750649535	T	T	T
0.5354252048469611	0.3242459682378345	0.8913363278957378	T	T	T
0.3404513717566346	0.2231412370542789	0.9464487232746642	T	T	T
0.4974337700101599	0.9886354543925954	0.9630363427425735	T	T	T
0.0193509540581740	0.4238592274118064	0.9455232386128258	T	T	T

0.6447020070599415	0.5357936763941067	0.9414054206208784	T	T	T
0.4341265087574525	0.7205368247921796	0.9378875087336532	T	T	T
0.4605728859288017	0.7116008356415924	0.8308316551350665	T	T	T
0.1842181715468821	0.7493748907215891	0.7484020328531331	T	T	T
0.0172083147148714	0.9486707787150528	0.9608426699114389	T	T	T
0.7020688707290771	0.7135260305901541	0.8694140475333189	T	T	T
0.1241610803276790	0.4757469565172894	0.8292911314531765	T	T	T
0.1796035356954441	0.6525399747932917	0.9618131075439726	T	T	T
0.8700234511062421	0.9327971931936214	0.8898344409766069	T	T	T
0.5695403344194203	0.8920439419751207	0.6975038563946779	T	T	T
0.3252305682572514	0.9521851795810338	0.6551346096563061	T	T	T
0.0777206959819792	0.9930534201938580	0.8427670492343103	T	T	T
0.6993024512023116	0.1318613852522471	0.9231944977354284	T	T	T
0.7672447569966271	0.0952554899050052	0.7189545340453949	T	T	T
0.3313516019446844	0.2024926639474860	0.7760587456570175	T	T	T
0.4057110992983424	0.9110378995033800	0.7645610223151791	T	T	T
0.4938319993332561	0.4109779657243551	0.8090276125663728	T	T	T
0.9988249342462659	0.9688249983459530	0.7607780112028385	T	T	T
0.1500557921935997	0.2426365504171437	0.8857456072875711	T	T	T
0.9300003398431436	0.2089664740292848	0.6530056927563361	T	T	T
0.1419689305556985	0.1098843238572553	0.6989181351427688	T	T	T
0.7166321242306378	0.6419071280176525	0.7124267067337386	T	T	T
0.8751259749485829	0.3921230604615554	0.8320845169766471	T	T	T
0.6235572996855345	0.5319199666782765	0.3814515488270684	T	T	T
0.8276374272439780	0.9471348106536650	0.5502930043467676	T	T	T
0.7150978978811444	0.3706092222904217	0.7608478998172029	T	T	T
0.7870119314097582	0.7827707948808502	0.7853854908956561	T	T	T
0.0664200296617174	0.0011922563880415	0.3706418011152716	T	T	T
0.0611603707750210	0.2359508881785476	0.4253849202486056	T	T	T
0.0938540082434759	0.3859056581387218	0.7428538788056123	T	T	T
0.9532308879883724	0.2157025738024617	0.5657044484798543	T	T	T
0.6184503916717145	0.9907548460662000	0.6048060668243714	T	T	T
0.8685596082383270	0.3149653679010742	0.4816135377427272	T	T	T
0.8735128406120183	0.7915102111533230	0.3763830473737256	T	T	T
0.4826326009746494	0.0222927675561220	0.3428883351119076	T	T	T
0.6088114441530506	0.6731027416252817	0.4705616743089590	T	T	T
0.7855407698836057	0.0416816197640818	0.4642125513336328	T	T	T
0.6398773588209555	0.8936473968395624	0.4076993840160316	T	T	T
0.4178242652664563	0.6484986956135730	0.3831928900147563	T	T	T
0.2845096685530958	0.6472620481065087	0.3767352934248575	T	T	T
0.4938396133046464	0.5192127731584992	0.3745138393779664	T	T	T
0.2234347293998295	0.5127020129476548	0.3627402514109320	T	T	T
0.0917765659454439	0.5117645222347871	0.3505361563073950	T	T	T
0.7041984468075397	0.4003936396988401	0.3745561191591098	T	T	T

```
0.1930743919568102    0.7733302432058858    0.5290227766835104 T T T
0.4339475557522769    0.3861796706023915    0.3598004039338755 T T T
0.2991340613035613    0.3821704444705646    0.3551300831189602 T T T
0.1943656837111400    0.8350345822375904    0.4825199996986861 T T T
0.3087773866140041    0.9422767611556172    0.4730785803149963 T T T
0.2847376521429369    0.0959257025603068    0.4938883069436279 T T T
0.4987208410263103    0.1508598685387824    0.5283362735381104 T T T
0.3566307734267977    0.3585869349536187    0.5052114608353182 T T T
0.4664044154318647    0.2047222084095369    0.4508479733353237 T T T
0.9851362748079592    0.5057708076292859    0.3358533387576493 T T T
0.4021924429173781    0.2015411202818246    0.4945685160041077 T T T
```

Adsorption model of different surface

0 °

relax:

```
   1.00000000000000
    10.2424999999999997    0.0000000000000000    0.0000000000000000
     0.0000000000000000    8.8702000000000005    0.0000000000000000
     0.0000000000000000    0.0000000000000000   21.2722000000000016
   Cu
    64
Selective dynamics
Direct
  0.0000000873523404  0.0001209916301978  0.3815758139260162   T   T   T
  0.2500003272120590  0.5001207202094650  0.3815755222873618   T   T   T
  0.5000000489152798  0.0001208204891625  0.3815759131505284   T   T   T
  0.6249999871130374  0.7501720216893357  0.3815187923839765   T   T   T
  0.8749999404806574  0.7501719158786779  0.3815188009083638   T   T   T
  0.3750000765334959  0.7501717692739064  0.3815188151205833   T   T   T
  0.8749998289768345  0.2501714121929946  0.3815182570079958   T   T   T
  0.7500001040300889  0.5001206114879069  0.3815753892932479   T   T   T
  0.6250000351191257  0.2501715953138811  0.3815197991218055   T   T   T
  0.1249999930261226  0.7501721866354203  0.3815186075413577   T   T   T
  0.3750000969448789  0.2501716398067647  0.3815182251299379   T   T   T
  0.4999997801356026  0.5001206868565151  0.3815753502862895   T   T   T
  0.2499997901882783  0.0001209469285042  0.3815758499742458   T   T   T
  0.7500000706860106  0.0001208876201218  0.3815758774224913   T   T   T
 -0.0000002171696542  0.5001207663663411  0.3815755747233169   T   T   T
  0.1250000486000822  0.2501712323708721  0.3815184831829893   T   T   T
  0.7499996340758480  0.3331497466305081  0.2847561226103456   T   T   T
 -0.0000002593263852  0.8331424949116591  0.2847582383972068   T   T   T
  0.5000000641713833  0.8331424725545842  0.2847583396328860   T   T   T
  0.2500000014804025  0.3331500070341724  0.2847561076213526   T   T   T
  0.0000001265399208  0.3331500012344140  0.2847558903552332   T   T   T
```

0.5000002389159427	0.3331497843096665	0.2847561824224387	T	T	T
0.1249998729789680	0.0831049717773425	0.2849156105669159	T	T	T
0.2500001328009692	0.8331424549972880	0.2847582117422165	T	T	T
0.6250000628652241	0.0831050635118716	0.2849164251344202	T	T	T
0.3749999425578439	0.5831046711832052	0.2849155850509912	T	T	T
0.7500000624986850	0.8331424770199767	0.2847584265142323	T	T	T
0.8750000976020712	0.5831048990972254	0.2849158958987332	T	T	T
0.6249999184193008	0.5831048746189714	0.2849161274425536	T	T	T
0.3750000427722067	0.0831049120539735	0.2849160582566791	T	T	T
0.8750000184013210	0.0831046723557291	0.2849157386269113	T	T	T
0.1250000399670918	0.5831046379842625	0.2849164431344639	T	T	T
0.4999999489001590	0.1666077593472293	0.1904100036537699	T	T	T
0.2499995461454453	0.6666084729488123	0.1904122086318749	T	T	T
0.8750001198573969	0.4172420052699373	0.1906851007410123	T	T	T
0.1249998985815474	0.9172427487525107	0.1906811165675044	T	T	T
0.6249998868447749	0.9172430635807483	0.1906814645428623	T	T	T
0.3749999486576995	0.4172419113828280	0.1906849509787545	T	T	T
0.7499998436612344	0.6666086077227068	0.1904127391124524	T	T	T
-0.0000001391023941	0.1666080599852367	0.1904099503449174	T	T	T
0.2500003228964509	0.1666079800550510	0.1904100177447984	T	T	T
0.0000002882025228	0.6666084944281532	0.1904123426019600	T	T	T
0.8750001251708019	0.9172430052845547	0.1906813353255506	T	T	T
0.7499998654428182	0.1666078821478358	0.1904098657260253	T	T	T
0.5000003230684036	0.6666087087769598	0.1904126219362102	T	T	T
0.1249999348852478	0.4172423654590655	0.1906850431650152	T	T	T
0.3750000904794232	0.9172431677926520	0.1906814911204014	T	T	T
0.6249999941784290	0.4172421599384248	0.1906849071791196	T	T	T
0.2500000000000000	0.0000000000000000	0.0940200000000004	F	F	F
0.6250000000000000	0.7500000000000000	0.0940200000000004	F	F	F
0.8750000000000000	0.2500000000000000	0.0940200000000004	F	F	F
0.0000000000000000	0.5000000000000000	0.0940200000000004	F	F	F
0.8750000000000000	0.7500000000000000	0.0940200000000004	F	F	F
0.1250000000000000	0.7500000000000000	0.0940200000000004	F	F	F
0.3750000000000000	0.2500000000000000	0.0940200000000004	F	F	F
0.7500000000000000	0.0000000000000000	0.0940200000000004	F	F	F
0.5000000000000000	0.5000000000000000	0.0940200000000004	F	F	F
0.3750000000000000	0.7500000000000000	0.0940200000000004	F	F	F
0.6250000000000000	0.2500000000000000	0.0940200000000004	F	F	F
0.7500000000000000	0.5000000000000000	0.0940200000000004	F	F	F
0.1250000000000000	0.2500000000000000	0.0940200000000004	F	F	F
0.2500000000000000	0.5000000000000000	0.0940200000000004	F	F	F
0.5000000000000000	0.0000000000000000	0.0940200000000004	F	F	F
0.0000000000000000	0.0000000000000000	0.0940200000000004	F	F	F

```
adsorption:
   1.00000000000000
     10.2424999999999997    0.0000000000000000    0.0000000000000000
      0.0000000000000000    8.8702000000000005    0.0000000000000000
      0.0000000000000000    0.0000000000000000   21.2722000000000016
   Cu   C   O   N   H
    64    8    1    1    7
Selective dynamics
Direct
  0.9986092485026846  0.9985867663816788  0.3817010068272272   T   T   T
  0.2438301413938191  0.4968303614417642  0.3827774398376259   T   T   T
  0.4989332811559127  0.9993802423160240  0.3823478747100706   T   T   T
  0.6234656710332396  0.7496120806340064  0.3825477046762708   T   T   T
  0.8735530819746502  0.7496284743775188  0.3822746278011039   T   T   T
  0.3747305101521711  0.7514170027289240  0.3798212242661099   T   T   T
  0.8733072149918949  0.2499243712411139  0.3813821143916769   T   T   T
  0.7479374606440633  0.5000345130023260  0.3827433060245313   T   T   T
  0.6234786974449248  0.2502887623161507  0.3820296949916223   T   T   T
  0.1225131626828118  0.7513786178949952  0.3799743632068582   T   T   T
  0.3757366660057508  0.2464538258434250  0.3799044559196990   T   T   T
  0.5012668506018233  0.4994520176579953  0.3792892702322495   T   T   T
  0.2492218603194005  0.9989415590315495  0.3803921871809141   T   T   T
  0.7487366860164610 -0.0000038257227140  0.3812594558709661   T   T   T
  0.9934804207726226  0.4995230833472651  0.3798722170406553   T   T   T
  0.1210017126145700  0.2438357123127008  0.3796833997987755   T   T   T
  0.7488911961540640  0.3327251175740945  0.2852484013266275   T   T   T
  0.9985178705176905  0.8327491720881294  0.2845065507972293   T   T   T
  0.4996766738549236  0.8328811194544063  0.2847698747299354   T   T   T
  0.2488533201973379  0.3326930763893174  0.2851655208783336   T   T   T
 -0.0004281066863889  0.3331837328890709  0.2844308082188453   T   T   T
  0.4985090801216189  0.3331050343979220  0.2843662398942775   T   T   T
  0.1241238021124012  0.0817749697048983  0.2838865024500608   T   T   T
  0.2490179760532291  0.8303404399354399  0.2839862879351706   T   T   T
  0.6242287491847152  0.0833270376817420  0.2852958235085408   T   T   T
  0.3723579032628935  0.5812934226162675  0.2852311159339124   T   T   T
  0.7490503894048879  0.8337100353225111  0.2854784735899253   T   T   T
  0.8736030680830579  0.5838722893107776  0.2846881786659858   T   T   T
  0.6238980205697912  0.5842498594865800  0.2847822990159101   T   T   T
  0.3745993926718144  0.0819762442085090  0.2841347337622456   T   T   T
  0.8742480245939390  0.0830629321694435  0.2851968112091742   T   T   T
  0.1244452213899584  0.5816157125499399  0.2849728624430984   T   T   T
  0.5002033429370557  0.1667128449004620  0.1905932830519530   T   T   T
  0.2494530468505571  0.6662779706116708  0.1898429051432657   T   T   T
  0.8744817985171941  0.4171625377999076  0.1908640056656050   T   T   T
```

0.1238896474743469	0.9163647794070143	0.1903014692378616	T	T	T
0.6247890525280508	0.9173309211315153	0.1908918977528244	T	T	T
0.3748110212372087	0.4166245038179721	0.1901408357736467	T	T	T
0.7493570769935319	0.6682356187116200	0.1908800445896345	T	T	T
0.9990818679555010	0.1667541717964913	0.1905656304113485	T	T	T
0.2497442012815361	0.1680964245672809	0.1901259339952868	T	T	T
-0.0002124529633860	0.6664901183132244	0.1906539281887464	T	T	T
0.8740804448540197	0.9173948011624786	0.1907783576441968	T	T	T
0.7496306571012500	0.1670926112991866	0.1908039153829698	T	T	T
0.4989550380578497	0.6664645851909899	0.1908553998930064	T	T	T
0.1242803939977992	0.4164172585403039	0.1900273101705069	T	T	T
0.3751632932792140	0.9165285272887103	0.1904924509365081	T	T	T
0.6244820702608128	0.4172127682248234	0.1909126352545454	T	T	T
0.2500000000000000	0.0000000000000000	0.0940200000000004	F	F	F
0.6250000000000000	0.7500000000000000	0.0940200000000004	F	F	F
0.8750000000000000	0.2500000000000000	0.0940200000000004	F	F	F
0.0000000000000000	0.5000000000000000	0.0940200000000004	F	F	F
0.8750000000000000	0.7500000000000000	0.0940200000000004	F	F	F
0.1250000000000000	0.7500000000000000	0.0940200000000004	F	F	F
0.3750000000000000	0.2500000000000000	0.0940200000000004	F	F	F
0.7500000000000000	0.0000000000000000	0.0940200000000004	F	F	F
0.5000000000000000	0.5000000000000000	0.0940200000000004	F	F	F
0.3750000000000000	0.7500000000000000	0.0940200000000004	F	F	F
0.6250000000000000	0.2500000000000000	0.0940200000000004	F	F	F
0.7500000000000000	0.5000000000000000	0.0940200000000004	F	F	F
0.1250000000000000	0.2500000000000000	0.0940200000000004	F	F	F
0.2500000000000000	0.5000000000000000	0.0940200000000004	F	F	F
0.5000000000000000	0.0000000000000000	0.0940200000000004	F	F	F
0.0000000000000000	0.0000000000000000	0.0940200000000004	F	F	F
0.8244987741215429	0.7526037281974745	0.6663031662882400	T	T	T
0.4736036692110348	0.7754718377202109	0.6327788089694991	T	T	T
0.6052177795289739	0.7304521729618433	0.6276351272128966	T	T	T
0.3799457004395173	0.7109630292910397	0.5945680325632351	T	T	T
0.6436113941470581	0.6227414552937023	0.5824522320910741	T	T	T
0.4174725442295238	0.5994451048005243	0.5508277958466138	T	T	T
0.5492930766712905	0.5567351413471271	0.5445546861398267	T	T	T
0.3242915650891534	0.5353694856081082	0.5108717664414943	T	T	T
0.6885419225666680	0.7969567630242160	0.6682848339038365	T	T	T
0.2509150681169066	0.4848811858996493	0.4739661622280683	T	T	T
0.8716844980360692	0.8172764797642832	0.7036477684696781	T	T	T
0.8346661026442437	0.6312444367351356	0.6753937378988824	T	T	T
0.4471916183566246	0.8602798642971983	0.6675260526265362	T	T	T
0.8673620189884371	0.7815199564795559	0.6205104700741536	T	T	T
0.2780877199679933	0.7448242493684634	0.5979107097758533	T	T	T

0.7450610571104219 0.5896924874509797 0.5769341493484460 T T T
0.5774356005624721 0.4736158613783970 0.5092409111565188 T T T

77 °
relax:
1.00000000000000
17.9242999999999988 0.0000000000000000 0.0000000000000000
0.0000000000000000 7.6818999999999997 0.0000000000000000
0.0000000000000000 0.0000000000000000 25.8638000000000012
Cu
93
Selective dynamics
Direct
0.4285726291558533 0.3333319052711370 0.4669427532030143 T T T
0.4285716380839231 0.6666721997003475 0.4669422084588746 T T T
0.4285736807037493 0.0000003288023386 0.4669421918274852 T T T
0.5026220649254028 0.5000015650392899 0.4061690427996012 T T T
0.3545223341685692 0.8333363606297874 0.4061693695937827 T T T
0.3545204439161066 0.4999992069183067 0.4061686847941300 T T T
0.3545222369210662 0.1666656326352161 0.4061699430764333 T T T
0.5026211527592271 0.1666662022985975 0.4061695042490435 T T T
0.5026212827721542 0.8333345883573331 0.4061693291967614 T T T
0.4285695436600443 -0.0000006448803743 0.3406137949739513 T T T
0.5729695559674172 -0.0000004768166394 0.3411783544835624 T T T
0.2841708540729970 0.3333353331117692 0.3411787126376712 T T T
0.5729831026270051 0.6666630709026982 0.3411833983954041 T T T
0.2841620787806858 0.6666630013696032 0.3411826375479504 T T T
0.5729739854287177 0.3333399353756891 0.3411810901090108 T T T
0.4285711887444624 0.6666659192303100 0.3406110537884181 T T T
0.2841627583038100 -0.0000005757995472 0.3411815608612524 T T T
0.4285706399145155 0.3333326481156372 0.3406113771415145 T T T
0.2117852884558553 0.1666688279950708 0.2773969578996592 T T T
0.5003406944639409 0.1666668488057748 0.2760424701844200 T T T
0.5003344305626183 0.5000002280757885 0.2760403833654154 T T T
0.3568035084223868 0.8333298740979810 0.2760414957816369 T T T
0.6453627344351834 0.1666695299458230 0.2773971057469777 T T T
0.3568087719523813 0.5000012410843493 0.2760375196740631 T T T
0.2117863972107508 0.4999999720922018 0.2773953727318298 T T T
0.6453619720320258 0.8333308033978460 0.2773981033102286 T T T
0.3568004207607542 0.1666663548729108 0.2760414706144963 T T T
0.5003379892452832 0.8333271646216822 0.2760421857057147 T T T
0.6453578782477815 0.4999975757641674 0.2773967767220910 T T T
0.2117852569246264 0.8333303605543617 0.2773976615027374 T T T
0.5735806440942035 0.3333271702638253 0.2106229912915944 T T T

0.4285715519067777	0.6666571531374045	0.2101530529509806	T	T	T
0.1387171573105103	0.6666714701256198	0.2136556079948068	T	T	T
0.2835613719037281	0.3333286714691294	0.2106186390827946	T	T	T
0.7184271872834638	0.0000004898832109	0.2136499101406321	T	T	T
-0.0010847694981354	0.3333355834169213	0.2116887275970041	T	T	T
0.8582250847686241	0.3333354584049291	0.2116895586843965	T	T	T
0.7184219459623956	0.6666692350118242	0.2136443058292191	T	T	T
0.1387153342076598	0.0000012240753056	0.2136569932850100	T	T	T
0.4285716365514131	-0.0000097477994764	0.2101506588083731	T	T	T
-0.0010838450103161	0.0000052140616677	0.2116888042573227	T	T	T
0.2835596100213636	0.0000000458097558	0.2106218882770736	T	T	T
0.8582258882713801	0.6666663874496941	0.2116894584980422	T	T	T
0.5735808479836738	0.6666676854916824	0.2106234210877264	T	T	T
0.7184221479788997	0.3333299951750998	0.2136427544793551	T	T	T
0.8582314842293126	0.0000032536712895	0.2116899985213055	T	T	T
0.2835607457128397	0.6666703257585367	0.2106200387160618	T	T	T
0.4285716323107727	0.3333519140726043	0.2101508459119488	T	T	T
0.5735840673794809	-0.0000005583182026	0.2106248699032617	T	T	T
-0.0010841594414819	0.6666688148958959	0.2116884421256605	T	T	T
0.1387166386832805	0.3333310748534956	0.2136553823242990	T	T	T
0.5008725720354412	0.8333356441809493	0.1431236989909294	T	T	T
0.9285717506855072	0.8333313437925747	0.1436236192818960	T	T	T
0.6450457596554936	0.5000019625241403	0.1437177230955137	T	T	T
0.7861103872650971	0.1666631784767124	0.1440807462530522	T	T	T
0.9285715338569890	0.5000046331180698	0.1436223850659668	T	T	T
0.2120942595879658	0.8333352631617044	0.1437165048298256	T	T	T
0.7861139729484817	0.8333321988479614	0.1440842563749959	T	T	T
0.0710344043542804	0.1666665672018010	0.1440873891966019	T	T	T
0.3562687768177795	0.5000017499266072	0.1431199205501956	T	T	T
0.3562691091245884	0.1666657591169504	0.1431215291955885	T	T	T
0.6450461638426068	0.8333365880536278	0.1437153667546371	T	T	T
0.5008727377279638	0.1666642640059539	0.1431239726405079	T	T	T
0.0710349363458384	0.5000011590856909	0.1440878743430681	T	T	T
0.7861122737661443	0.4999990898682842	0.1440872686999502	T	T	T
0.2120942307598789	0.1666659884325566	0.1437166181308109	T	T	T
0.2120941003784761	0.5000006002619528	0.1437165858261198	T	T	T
0.9285708848913973	0.1666721276989994	0.1436199685601259	T	T	T
0.0710345382920411	0.8333336607466872	0.1440867034663924	T	T	T
0.6450457244039135	0.1666649364251578	0.1437152340204322	T	T	T
0.3562689204854979	0.8333344008670002	0.1431218326579124	T	T	T
0.5008736759620598	0.5000006367570443	0.1431221446992491	T	T	T
0.0000000000000000	0.3333333332999970	0.0773300000000035	F	F	F
0.7142899999999983	0.0000000000000000	0.0773300000000035	F	F	F
0.8571400000000011	0.6666700000000034	0.0773300000000035	F	F	F

```
    0.5714299999999994    0.6666700000000034    0.0773300000000035    F    F    F
    0.7142899999999983    0.3333333332999970    0.0773300000000035    F    F    F
    0.4285700000000006    0.0000000000000000    0.0773300000000035    F    F    F
    0.8571400000000011    0.0000000000000000    0.0773300000000035    F    F    F
    0.1428599999999989    0.0000000000000000    0.0773300000000035    F    F    F
    0.7142899999999983    0.6666700000000034    0.0773300000000035    F    F    F
    0.2857100000000017    0.6666700000000034    0.0773300000000035    F    F    F
    0.8571400000000011    0.3333333332999970    0.0773300000000035    F    F    F
    0.4285700000000006    0.3333333332999970    0.0773300000000035    F    F    F
    0.4285700000000006    0.6666700000000034    0.0773300000000035    F    F    F
    0.5714299999999994    0.0000000000000000    0.0773300000000035    F    F    F
    0.5714299999999994    0.3333333332999970    0.0773300000000035    F    F    F
    0.0000000000000000    0.6666700000000034    0.0773300000000035    F    F    F
    0.1428599999999989    0.6666700000000034    0.0773300000000035    F    F    F
    0.1428599999999989    0.3333333332999970    0.0773300000000035    F    F    F
    0.2857100000000017    0.3333333332999970    0.0773300000000035    F    F    F
    0.2857100000000017    0.0000000000000000    0.0773300000000035    F    F    F
    0.0000000000000000    0.0000000000000000    0.0773300000000035    F    F    F
```

adsorption：

```
   1.00000000000000
     17.9242999999999988    0.0000000000000000    0.0000000000000000
      0.0000000000000000    7.6818999999999997    0.0000000000000000
      0.0000000000000000    0.0000000000000000   25.8638000000000012
   Cu   C    O    N    H
    93    8    1    1    7
Selective dynamics
Direct
    0.4287025531451973    0.3292834111278214    0.4666645992229796    T    T    T
    0.4295316089251797    0.6658657051017226    0.4710363600001852    T    T    T
    0.4288384040654373    0.0019486739947541    0.4672645532162836    T    T    T
    0.5020147267007561    0.5001850835159046    0.4058622627185224    T    T    T
    0.3570820696972790    0.8294397969182058    0.4063393208343500    T    T    T
    0.3568059239908811    0.5019407322236245    0.4061809156623817    T    T    T
    0.3548642536271040    0.1656167208308183    0.4059092088245844    T    T    T
    0.5033816189219592    0.1659909008660247    0.4059639374690692    T    T    T
    0.5018063632735322    0.8322892173233043    0.4059819745867449    T    T    T
    0.4292162068233472 -0.0006437719242392    0.3401825014472170    T    T    T
    0.5725972553250773 -0.0008096627994873    0.3406875901432687    T    T    T
    0.2853733824577895    0.3334703737279420    0.3410273855031071    T    T    T
    0.5725700878709238    0.6664010048776623    0.3407526754384912    T    T    T
    0.2852521476868230    0.6660734590030648    0.3410419053406112    T    T    T
    0.5726724022148399    0.3332914985996606    0.3407071267874843    T    T    T
    0.4292888687668036    0.6660017157488420    0.3399859084117187    T    T    T
```

0.2854177735327379	-0.0016702562325555	0.3411040851417292	T	T	T
0.4292324764353092	0.3328944537783877	0.3402638056306706	T	T	T
0.2122290972002698	0.1661417280001623	0.2775732008580744	T	T	T
0.5005813312163033	0.1663613644186686	0.2752059656854601	T	T	T
0.5004585247908825	0.4997538061618982	0.2749420980673509	T	T	T
0.3574200431907427	0.8325706921709659	0.2753685624065149	T	T	T
0.6457677740108455	0.1664479191603515	0.2774032631909247	T	T	T
0.3574221856444974	0.4996943760560392	0.2753284287354785	T	T	T
0.2123178499946543	0.4996318633552443	0.2774774286231936	T	T	T
0.6457088205864030	0.8330687693071214	0.2773609621731138	T	T	T
0.3572662824732932	0.1662620974506180	0.2758703572599191	T	T	T
0.5004655704008317	0.8326841102158387	0.2749586918584839	T	T	T
0.6456112110856159	0.4998041582343086	0.2773529746653906	T	T	T
0.2123323778406528	0.8328727386555490	0.2774800798662719	T	T	T
0.5742933549779660	0.3331141992689760	0.2101159970278377	T	T	T
0.4287579678640864	0.6663208015950328	0.2093129534872489	T	T	T
0.1384580723362141	0.6662094593281348	0.2141166114380257	T	T	T
0.2835388445843074	0.3328282722210170	0.2105915625705387	T	T	T
0.7189745031463315	-0.0001901325642383	0.2136566127109562	T	T	T
-0.0009491477659589	0.3336400341829148	0.2118200597369916	T	T	T
0.8585149894590628	0.3334652876077554	0.2118270431786534	T	T	T
0.7191794639379246	0.6664157778752785	0.2139144743827006	T	T	T
0.1385897333339077	-0.0004374338065146	0.2139174407271724	T	T	T
0.4287003684770430	0.0000761526805609	0.2094445737379088	T	T	T
-0.0008697220686483	-0.0000663742840690	0.2118027797355546	T	T	T
0.2836067726842732	-0.0000351813145936	0.2106476807777503	T	T	T
0.8587568969311202	0.6665942576392346	0.2118772974806771	T	T	T
0.5744747953480784	0.6664407824910767	0.2099870266794467	T	T	T
0.7189467484843745	0.3330480494830805	0.2136961464408279	T	T	T
0.8586275488048130	-0.0001856592358630	0.2118147358236792	T	T	T
0.2833638331959688	0.6663932193400831	0.2103585271255421	T	T	T
0.4286864421644319	0.3325599617392976	0.2093811933412879	T	T	T
0.5743132500937941	-0.0002188598443147	0.2101881567929770	T	T	T
-0.0009848375362423	0.6667533569559775	0.2118674138140292	T	T	T
0.1385503496080061	0.3329371914183334	0.2139290820778073	T	T	T
0.5011919556768828	0.8332827760225321	0.1429964821943174	T	T	T
0.9286966454505525	0.8332097725042290	0.1436424907754310	T	T	T
0.6457218266467971	0.4998439614222432	0.1436256211222506	T	T	T
0.7864722576968859	0.1665833684480542	0.1441648656015110	T	T	T
0.9286981348209586	0.5000819367613535	0.1436364819510336	T	T	T
0.2119315261115671	0.8332954699452118	0.1438141162196995	T	T	T
0.7864664580368695	0.8331549777890163	0.1441537064656264	T	T	T
0.0710326153013398	0.1665003599778210	0.1441774618317365	T	T	T
0.3560450961819532	0.4997822557939245	0.1430519136113548	T	T	T

0.3559354173091183	0.1664614477491677	0.1429313851983370	T	T	T
0.6456929356589253	0.8333011044766311	0.1436319097041482	T	T	T
0.5011793337698091	0.1665324471398048	0.1429485557662346	T	T	T
0.0710508359919848	0.4999583862896891	0.1441701151617845	T	T	T
0.7864581447176809	0.4999289506451866	0.1441845683098318	T	T	T
0.2120779928089391	0.1664350636924256	0.1437790411751320	T	T	T
0.2118797772798332	0.4996920096292111	0.1438120156525481	T	T	T
0.9287639872523347	0.1666894418366643	0.1437570453675843	T	T	T
0.0710579596767154	0.8330096469973861	0.1441778415909268	T	T	T
0.6454510534295950	0.1665316003930278	0.1435739665401115	T	T	T
0.3560637037268327	0.8334083440056623	0.1430617139608168	T	T	T
0.5012091148530353	0.4998251316632628	0.1429769830311130	T	T	T
0.0000000000000000	0.3333333332999970	0.0773300000000035	F	F	F
0.7142899999999983	0.0000000000000000	0.0773300000000035	F	F	F
0.8571400000000011	0.6666700000000034	0.0773300000000035	F	F	F
0.5714299999999994	0.6666700000000034	0.0773300000000035	F	F	F
0.7142899999999983	0.3333333332999970	0.0773300000000035	F	F	F
0.4285700000000006	0.0000000000000000	0.0773300000000035	F	F	F
0.8571400000000011	0.0000000000000000	0.0773300000000035	F	F	F
0.1428599999999989	0.0000000000000000	0.0773300000000035	F	F	F
0.7142899999999983	0.6666700000000034	0.0773300000000035	F	F	F
0.2857100000000017	0.6666700000000034	0.0773300000000035	F	F	F
0.8571400000000011	0.3333333332999970	0.0773300000000035	F	F	F
0.4285700000000006	0.3333333332999970	0.0773300000000035	F	F	F
0.4285700000000006	0.6666700000000034	0.0773300000000035	F	F	F
0.5714299999999994	0.0000000000000000	0.0773300000000035	F	F	F
0.5714299999999994	0.3333333332999970	0.0773300000000035	F	F	F
0.0000000000000000	0.6666700000000034	0.0773300000000035	F	F	F
0.1428599999999989	0.6666700000000034	0.0773300000000035	F	F	F
0.1428599999999989	0.3333333332999970	0.0773300000000035	F	F	F
0.2857100000000017	0.3333333332999970	0.0773300000000035	F	F	F
0.2857100000000017	0.0000000000000000	0.0773300000000035	F	F	F
0.0000000000000000	0.0000000000000000	0.0773300000000035	F	F	F
0.7821381231871051	0.4491327224382025	0.7297667565682233	T	T	T
0.7009432567717477	0.6055520675191469	0.6721654367979309	T	T	T
0.6812426764271793	0.7670933803601726	0.6500721915589093	T	T	T
0.6619604580132950	0.4532868411447736	0.6585323220999103	T	T	T
0.6227019228241583	0.7775298552542448	0.6154614615218337	T	T	T
0.6029922951405228	0.4634597161078986	0.6237815007333223	T	T	T
0.5826293454670489	0.6251176163866036	0.6020733625207063	T	T	T
0.5231653736492360	0.6366366138433687	0.5666778511728460	T	T	T
0.7580488432329192	0.6105342689533363	0.7063919992295796	T	T	T
0.4745390626445256	0.6497365911723462	0.5365492365705083	T	T	T
0.7368183338187757	0.3904188875036921	0.7524291991049025	T	T	T

0.8282708255720986	0.4848612576574985	0.7552635692856098	T	T	T
0.8017626400298407	0.3578177548456768	0.7000507844153684	T	T	T
0.7125110295940046	0.8826752205063539	0.6609825839765047	T	T	T
0.6769743547381580	0.3277361916901559	0.6748333183410153	T	T	T
0.6071911612516043	0.9017587783963982	0.5984242037403140	T	T	T
0.5725271263396714	0.3462124217959301	0.6132074772205738	T	T	T

96 °
relax:

1.00000000000000

18.1063000000000009	0.0000000000000000	0.0000000000000000
0.0000000000000000	7.6818999999999997	0.0000000000000000
0.0000000000000000	0.0000000000000000	26.5228000000000002

Cu

120

Selective dynamics

Direct

0.5000007446701504	0.8333331276334101	0.5137594690203570	T	T	T
0.5000007100782908	0.4999996736072278	0.5137593392879823	T	T	T
0.5000006843215778	0.1666656115390406	0.5137609834764367	T	T	T
0.3986665316804506	0.0000024792923868	0.4723330868965109	T	T	T
0.3986647057624142	0.6666679728310632	0.4723313296289179	T	T	T
0.6013351199738967	0.3333286302708335	0.4723326044903697	T	T	T
0.3986660432567089	0.3333282461664937	0.4723333931966705	T	T	T
0.6013346309990546	0.0000019989397507	0.4723319459408972	T	T	T
0.6013366136269175	0.6666680793716020	0.4723303375007948	T	T	T
0.3020569655461059	0.1666664861783669	0.4262262713478118	T	T	T
0.5000010890586989	0.1666654857356157	0.4246726748645110	T	T	T
0.5000011434850588	0.8333332311069701	0.4246732139833419	T	T	T
0.6979411473140535	0.8333337557358126	0.4262236256729535	T	T	T
0.6979420746851772	0.1666664756517787	0.4262252094327578	T	T	T
0.3020581950535747	0.4999994138974144	0.4262253636577681	T	T	T
0.6979410115225294	0.4999990685708738	0.4262240574956671	T	T	T
0.5000010303401713	0.5000000557663294	0.4246729454577776	T	T	T
0.3020579576008457	0.8333341771566006	0.4262251485436106	T	T	T
0.2034013471607092	0.3333338475246675	0.3801288115264924	T	T	T
0.3996017849913114	0.3333323613954530	0.3783344278516328	T	T	T
0.6003977629527273	0.0000015406850871	0.3783306504607969	T	T	T
0.7965976192505811	-0.0000001118525448	0.3801288126117143	T	T	T
0.7965966077267660	0.6666660866249934	0.3801287202862185	T	T	T
0.6003980060519193	0.6666675886314961	0.3783293303415167	T	T	T
0.6003983002011897	0.3333324336764459	0.3783320050191410	T	T	T
0.7965975921314452	0.3333332373331174	0.3801286581747138	T	T	T
0.3996023880567585	0.0000022812169794	0.3783336555772913	T	T	T

0.2034013831120932	0.0000004479331214	0.3801290568355247	T	T	T
0.2034019802381062	0.6666665782946342	0.3801293879662624	T	T	T
0.3996022048631742	0.6666687263231831	0.3783326150906179	T	T	T
0.1026348895106568	0.8333335693239652	0.3364006219293966	T	T	T
0.3002498889677879	0.8333337079701417	0.3310877441708052	T	T	T
0.1026329056257513	0.1666668531459013	0.3364009705147900	T	T	T
0.8973672471601357	0.1666674424387553	0.3364027286053755	T	T	T
0.3002501560376694	0.1666676056815136	0.3310888257146912	T	T	T
0.4999996509844249	0.8333342997193808	0.3322714863342923	T	T	T
0.6997496298022138	0.1666677249744727	0.3310870187727384	T	T	T
0.5000003972663037	0.1666670277909487	0.3322668618444430	T	T	T
0.1026350249122795	0.5000026765456178	0.3364005052049079	T	T	T
0.3002500738817674	0.4999994745445536	0.3310875981624103	T	T	T
0.4999999432637762	0.5000017981587673	0.3322724455812332	T	T	T
0.6997502683181497	0.8333331858430295	0.3310863623441814	T	T	T
0.8973653152365485	0.8333323885167345	0.3364023011798618	T	T	T
0.8973667234264496	0.5000025747046236	0.3364027082347867	T	T	T
0.6997489787823451	0.5000000829493880	0.3310858471260973	T	T	T
0.4001266563557439	0.0000046388338669	0.2848981983976117	T	T	T
-0.0000000154917108	0.6666728874595783	0.2927934485124525	T	T	T
0.1991270908021344	0.0000012096913055	0.2858337749414240	T	T	T
0.0000000042748844	0.3333396178795802	0.2927936689313542	T	T	T
0.4001247092280487	0.6666670196436195	0.2848981684337840	T	T	T
-0.0000005593782790	0.0000022141315732	0.2927929796075054	T	T	T
0.8008739307172688	0.0000007109012524	0.2858340205220614	T	T	T
0.1991270232234806	0.6666675272004837	0.2858329640696359	T	T	T
0.5998734103601941	0.0000027897463571	0.2848948714244529	T	T	T
0.5998724792778345	0.3333325849768046	0.2848966892182945	T	T	T
0.1991274216326045	0.3333343663011817	0.2858334751520596	T	T	T
0.8008737264880209	0.3333343248821914	0.2858342585411544	T	T	T
0.4001265961955829	0.3333325900097199	0.2848987731887548	T	T	T
0.8008741718944807	0.6666668008304611	0.2858334410090362	T	T	T
0.5998742188416445	0.6666689795478270	0.2848948972714226	T	T	T
0.9028723902837956	0.8333336527789482	0.2405518113021314	T	T	T
0.4999983123535191	0.8333345915941450	0.2378411891514990	T	T	T
0.2996202209057644	0.1666688145288495	0.2375266546893195	T	T	T
0.0971274788206411	0.1666674390708316	0.2405523931640312	T	T	T
0.7003808040364118	0.8333349242180237	0.2375249076508353	T	T	T
0.4999994296146215	0.1666677585475941	0.2378386218242732	T	T	T
0.0971276758541382	0.5000007303876813	0.2405512980464645	T	T	T
0.2996204424962601	0.4999970399936040	0.2375251278930801	T	T	T
0.0971283650978726	0.8333339952470924	0.2405518041750762	T	T	T
0.7003806152568829	0.1666699952496151	0.2375261031685881	T	T	T
0.9028732836506506	0.1666677184417612	0.2405524150581501	T	T	T

0.2996205391225335	0.8333360880810363	0.2375260509868137	T	T	T
0.4999988623051134	0.5000018961137229	0.2378435119088145	T	T	T
0.7003796376043444	0.4999977935266217	0.2375247655892583	T	T	T
0.9028731053787667	0.5000004856132954	0.2405518913650037	T	T	T
0.8022672172014212	0.6666674825571336	0.1921720205382091	T	T	T
0.8022666324637444	0.3333337730849065	0.1921724257953174	T	T	T
0.5999743654242843	0.3333387116014093	0.1907775487580082	T	T	T
0.4000273070327827	0.6666690558963035	0.1907780532235719	T	T	T
0.1977345234335221	0.6666667034507113	0.1921709915299151	T	T	T
0.5999716879834877	0.6666686596655469	0.1907766595666995	T	T	T
0.0000001360974832	0.0000018381200619	0.1934091541484141	T	T	T
0.1977346419070684	0.3333345619984915	0.1921710399440804	T	T	T
0.1977348549937136	0.0000014757901135	0.1921710845322339	T	T	T
0.4000252436107715	0.0000008331225238	0.1907781192198964	T	T	T
0.8022660223233127	0.0000015270968796	0.1921720195321855	T	T	T
0.5999741744111462	0.0000014656402115	0.1907768178630885	T	T	T
0.0000005465426560	0.3333337207356373	0.1934099790704345	T	T	T
0.0000002829503357	0.6666668604396927	0.1934094850748164	T	T	T
0.4000248623591149	0.3333384956849754	0.1907782831233898	T	T	T
0.2989365537287999	0.4999999947523429	0.1410415638523120	T	T	T
0.9004281143400034	0.5000000656855080	0.1426754387272842	T	T	T
0.0995723848406619	0.1666694547599876	0.1426748791610213	T	T	T
0.2989350117804986	0.1666676508179441	0.1410414365021044	T	T	T
0.7010645230451688	0.8333343691235872	0.1410413849189996	T	T	T
0.9004275054118445	0.8333338008952313	0.1426755101129904	T	T	T
0.7010643251163116	0.4999999512662054	0.1410414436228316	T	T	T
0.0995724577099109	0.5000015266187376	0.1426737407510557	T	T	T
0.4999968690257233	0.5000000273000783	0.1411266240660877	T	T	T
0.4999997520513488	0.1666718880966258	0.1411477081886102	T	T	T
0.4999997763483410	0.8333404079671402	0.1411271639866107	T	T	T
0.0995727350889940	0.8333343703540772	0.1426751687875522	T	T	T
0.2989363721363621	0.8333345637105795	0.1410416465300116	T	T	T
0.9004278625489269	0.1666684267127480	0.1426751988196291	T	T	T
0.7010658167545886	0.1666679431414735	0.1410418206362718	T	T	T
0.7999999999999972	0.3333333332999970	0.0997300000000010	F	F	F
0.7999999999999972	0.6666700000000034	0.0997300000000010	F	F	F
0.6000000000000014	0.6666700000000034	0.0997300000000010	F	F	F
0.0000000000000000	0.0000000000000000	0.0997300000000010	F	F	F
0.2000000000000028	0.0000000000000000	0.0997300000000010	F	F	F
0.3999999999999986	0.0000000000000000	0.0997300000000010	F	F	F
0.6000000000000014	0.3333333332999970	0.0997300000000010	F	F	F
0.3999999999999986	0.3333333332999970	0.0997300000000010	F	F	F
0.7999999999999972	0.0000000000000000	0.0997300000000010	F	F	F
0.6000000000000014	0.0000000000000000	0.0997300000000010	F	F	F

0.0000000000000000 0.6666700000000034 0.0997300000000010 F F F
0.0000000000000000 0.3333333332999970 0.0997300000000010 F F F
0.3999999999999986 0.6666700000000034 0.0997300000000010 F F F
0.2000000000000028 0.6666700000000034 0.0997300000000010 F F F
0.2000000000000028 0.3333333332999970 0.0997300000000010 F F F

adsorption：
1.00000000000000
18.1063000000000009 0.0000000000000000 0.0000000000000000
0.0000000000000000 7.6818999999999997 0.0000000000000000
0.0000000000000000 0.0000000000000000 26.5228000000000002
Cu C O N H
120 8 1 1 7
Selective dynamics
Direct
0.4988738434247463 0.8338318182944222 0.5132865916770997 T T T
0.4975202478586062 0.4987274775828979 0.5168985191376217 T T T
0.4991395951073697 0.1616616642713673 0.5132250464188163 T T T
0.3972185854381968 -0.0014274775889539 0.4716350359371668 T T T
0.3979015057470454 0.6666770035081329 0.4705188142348491 T T T
0.5996129895937873 0.3316556696054214 0.4720013158164593 T T T
0.3980602665415575 0.3303733874558955 0.4705297441342355 T T T
0.6005803863840042 -0.0019576487113309 0.4724123634395584 T T T
0.5996519002398014 0.6646107444280409 0.4721281350602162 T T T
0.3008220132537054 0.1651510039044609 0.4257446774499256 T T T
0.4996491691931902 0.1658203771170114 0.4238615580328171 T T T
0.4995997325470110 0.8316906301886562 0.4238872976886408 T T T
0.6959775701952789 0.8317060084339972 0.4261268004157047 T T T
0.6959763967674406 0.1656000388659873 0.4261359254967338 T T T
0.3008595674808098 0.4986097405473295 0.4259476045231094 T T T
0.6961158071041806 0.4987393703094287 0.4263199526576242 T T T
0.4996889830298932 0.4987082055601006 0.4261556370967854 T T T
0.3008557379855261 0.8321798155428107 0.4256629438743906 T T T
0.2014569713406262 0.3324843435635694 0.3802436058614992 T T T
0.3993211449999057 0.3327709842938249 0.3774161811734473 T T T
0.5997487355099610 -0.0010691430746541 0.3775250615284123 T T T
0.7958285179312083 -0.0008867806752256 0.3804810762500702 T T T
0.7956835234695895 0.6658065141521285 0.3804679750040980 T T T
0.5996734178091531 0.6653406779358830 0.3774156248296507 T T T
0.5996901334392180 0.3325554197642683 0.3773931217295600 T T T
0.7956668355470045 0.3324274239490720 0.3804641204202567 T T T
0.3991899494537131 -0.0010052836628707 0.3774065880812807 T T T
0.2013451701668080 -0.0008495884164207 0.3802301813336846 T T T
0.2014651594463391 0.6657004416679914 0.3802460270760765 T T T

0.3993158859655050	0.6650428020973390	0.3773883337332110	T	T	T
0.1006201693743902	0.8326754199448930	0.3366966073292161	T	T	T
0.2994204717415387	0.8324145172351706	0.3310834359974068	T	T	T
0.1006193141207176	0.1660740301326156	0.3366735165278498	T	T	T
0.8969244961656994	0.1660835408377013	0.3367893438747711	T	T	T
0.2993856491606393	0.1658284485513498	0.3311237582338311	T	T	T
0.4997095567152683	0.8322225184467903	0.3312408674068511	T	T	T
0.6995668616470472	0.1657080444740499	0.3311529182215348	T	T	T
0.4997048225853557	0.1657111842262618	0.3312013309590687	T	T	T
0.1006860663874203	0.4993630036170564	0.3366065745836117	T	T	T
0.2993639337929684	0.4992055824509080	0.3310584400508623	T	T	T
0.4997838692481582	0.4988453152973800	0.3319794333323420	T	T	T
0.6995603744200661	0.8324910901376702	0.3311445409803148	T	T	T
0.8969299148161067	0.8327341673550664	0.3367958983866852	T	T	T
0.8968355817247979	0.4994004612089011	0.3367073137167347	T	T	T
0.6995596957499143	0.4991493134607197	0.3310712162998385	T	T	T
0.3996525283644384	-0.0007753922965886	0.2841667957275814	T	T	T
-0.0009154211629657	0.6659556759696873	0.2926240120321791	T	T	T
0.1979619046911944	-0.0006416199030345	0.2862540775988455	T	T	T
-0.0009116789604441	0.3327003752533904	0.2926261026075682	T	T	T
0.3996823668533845	0.6657033477429412	0.2843625592190272	T	T	T
-0.0009298316894007	-0.0006858203487914	0.2925444565292233	T	T	T
0.8007937355280206	-0.0007549501903615	0.2861365646378177	T	T	T
0.1978543997276888	0.6660405952143201	0.2862598729303003	T	T	T
0.5998572712812478	-0.0007833722670703	0.2842151852554768	T	T	T
0.5998378757056295	0.3325664462030617	0.2842267235857616	T	T	T
0.1978850534446961	0.3325733806665309	0.2862657712485397	T	T	T
0.8008448195629239	0.3324977814988706	0.2861298149051474	T	T	T
0.3996529161023603	0.3326737350022542	0.2843753994568021	T	T	T
0.8008419486070243	0.6660307506373987	0.2861226606243306	T	T	T
0.5998086457154590	0.6658783790421346	0.2842352805356571	T	T	T
0.9021491527286425	0.8326998687268835	0.2407389259108339	T	T	T
0.4997843038747081	0.8326314714265808	0.2372193473231610	T	T	T
0.2989839938384865	0.1661080059641242	0.2376782714898232	T	T	T
0.0965338529164395	0.1659724181547181	0.2408416850437125	T	T	T
0.7004606222314744	0.8326691279594141	0.2375781320232201	T	T	T
0.4997881097052779	0.1660233760025648	0.2371876904677462	T	T	T
0.0965020772344811	0.4993342080562072	0.2407698021421396	T	T	T
0.2990063000312478	0.4994421750101314	0.2376022309054222	T	T	T
0.0965263233238798	0.8327408244059435	0.2408506516145931	T	T	T
0.7004644501977404	0.1661038833295800	0.2375730142090083	T	T	T
0.9021482808578677	0.1659385881460523	0.2407335427055230	T	T	T
0.2989991742734696	0.8328126764354356	0.2376644579291859	T	T	T
0.4998312599328828	0.4993400902557379	0.2372370416232645	T	T	T

0.7004512611814396	0.4993728624279994	0.2374841520975736	T	T	T
0.9021975205585989	0.4993383595190130	0.2406715215591065	T	T	T
0.8018809632735029	0.6661111586201199	0.1922746596648173	T	T	T
0.8018832570301507	0.3327785277044272	0.1922771047849193	T	T	T
0.6000477648632111	0.3327491383956000	0.1906011342640618	T	T	T
0.3997006545486232	0.6661139132147850	0.1906819418459080	T	T	T
0.1974369237319697	0.6662602847703341	0.1925005493074688	T	T	T
0.6000336190897096	0.6661253426965309	0.1906087456325243	T	T	T
-0.0003171787798510	-0.0007888782590022	0.1934268751267783	T	T	T
0.1974609025971146	0.3327309136979816	0.1924964611535541	T	T	T
0.1974907802537437	-0.0004458276050530	0.1924576931055542	T	T	T
0.3996751376730987	-0.0004580882189068	0.1904941741720367	T	T	T
0.8018237886814941	-0.0005587096250971	0.1922446059316115	T	T	T
0.6000448426928396	-0.0006004291993238	0.1905249938588223	T	T	T
-0.0003285150881785	0.3324473813308762	0.1934762777910959	T	T	T
-0.0003354461580316	0.6659900186002583	0.1934704879226880	T	T	T
0.3996726581932164	0.3328672906442114	0.1906988757953788	T	T	T
0.2987996247517748	0.4997742453679329	0.1411279933563633	T	T	T
0.9002176040663342	0.4996402465211988	0.1426703619062847	T	T	T
0.0996526237684603	0.1663285924509980	0.1426823199146954	T	T	T
0.2988554984329724	0.1664715401174666	0.1411306118206129	T	T	T
0.7009777765587732	0.8329993368804142	0.1409410326058108	T	T	T
0.9001952324088072	0.8329185672509789	0.1426383753149471	T	T	T
0.7009514383342005	0.4997033802889340	0.1408906801473092	T	T	T
0.0996630752839254	0.4996822261411142	0.1427780199101595	T	T	T
0.4999496858982019	0.4997618315210478	0.1406360829951784	T	T	T
0.4999331247881987	0.1666133646254584	0.1408149093285845	T	T	T
0.4999568499802534	0.8329137561136750	0.1408192351082848	T	T	T
0.0996506431370224	0.8329964050210222	0.1426786289649480	T	T	T
0.2988664024845119	0.8330666460497482	0.1411206205477002	T	T	T
0.9001996475164740	0.1663631093577769	0.1426344960837825	T	T	T
0.7009936136768909	0.1663996495062298	0.1409630405668868	T	T	T
0.7999999999999972	0.3333333332999970	0.0997300000000010	F	F	F
0.7999999999999972	0.6666700000000034	0.0997300000000010	F	F	F
0.6000000000000014	0.6666700000000034	0.0997300000000010	F	F	F
0.0000000000000000	0.0000000000000000	0.0997300000000010	F	F	F
0.2000000000000028	0.0000000000000000	0.0997300000000010	F	F	F
0.3999999999999986	0.0000000000000000	0.0997300000000010	F	F	F
0.6000000000000014	0.3333333332999970	0.0997300000000010	F	F	F
0.3999999999999986	0.3333333332999970	0.0997300000000010	F	F	F
0.7999999999999972	0.0000000000000000	0.0997300000000010	F	F	F
0.6000000000000014	0.0000000000000000	0.0997300000000010	F	F	F
0.0000000000000000	0.6666700000000034	0.0997300000000010	F	F	F
0.0000000000000000	0.3333333332999970	0.0997300000000010	F	F	F

0.3999999999999986	0.6666700000000034	0.0997300000000010	F	F	F
0.2000000000000028	0.6666700000000034	0.0997300000000010	F	F	F
0.2000000000000028	0.3333333332999970	0.0997300000000010	F	F	F
0.2099642016723175	0.4999989691870005	0.8120407199905697	T	T	T
0.2791224783195582	0.3985822152765536	0.7405322230908429	T	T	T
0.2990674688976223	0.2570802536854606	0.7095732672802901	T	T	T
0.3068231253891005	0.5664117795252100	0.7305248467784125	T	T	T
0.3465281603367128	0.2818527579091131	0.6693782582883232	T	T	T
0.3547297965042984	0.5916483996611773	0.6902371297015601	T	T	T
0.3752141960107184	0.4500293636081263	0.6594554727785604	T	T	T
0.4234936365455696	0.4735815684206925	0.6183902283618236	T	T	T
0.2329088171149922	0.3605157846781346	0.7790806511970892	T	T	T
0.4629903734144630	0.4882363961514007	0.5836375007117256	T	T	T
0.1751655840015960	0.4385190657777129	0.8406771880355047	T	T	T
0.2579843882764820	0.5612378182537099	0.8301365259378014	T	T	T
0.1779370989334496	0.5978688704860088	0.7911735226294809	T	T	T
0.2764869173034397	0.1287031761061189	0.7177538827701505	T	T	T
0.2911923921368090	0.6769109694905506	0.7537674300119251	T	T	T
0.3618665999432922	0.1732467345002641	0.6452232060924225	T	T	T
0.3763555236195825	0.7209718865012158	0.6824311562873614	T	T	T

126.9 °

relax:

1.00000000000000

17.9242999999999988	0.0000000000000000	0.0000000000000000
0.0000000000000000	7.6818999999999997	0.0000000000000000
0.0000000000000000	0.0000000000000000	25.8638000000000012

Cu

123

Selective dynamics

Direct

0.5710532864525860	0.6666665522729313	0.4776523198526548	T	T	T
0.8505828526133802	0.3333323120657336	0.4742584774113880	T	T	T
0.7103576294331050	0.0000039024031441	0.4777377551997655	T	T	T
0.5710537819524029	0.3333358509716294	0.4776517766084646	T	T	T
0.4298210437621883	0.3333344963786026	0.4735482209587739	T	T	T
0.8505808372320401	0.0000015437092968	0.4742587684030132	T	T	T
0.5710539497245696	0.0000008654889029	0.4776529063693279	T	T	T
0.7103544312235508	0.6666694430285445	0.4777382099367171	T	T	T
0.4298210911238107	0.0000007602883141	0.4735479282504997	T	T	T
0.4298210054711835	0.6666684600482981	0.4735479330151365	T	T	T
0.7103549016158648	0.3333347230120745	0.4777380636068529	T	T	T
0.8505814826170143	0.6666689431129886	0.4742592731549605	T	T	T
0.5006872703029077	0.8333343973229630	0.4103566082243907	T	T	T

0.9216758349161942	0.8333335546695124	0.4088788295827103	T	T	T
0.9216760769122205	0.5000009999728244	0.4088802464072243	T	T	T
0.7821561000735816	0.8333348512417018	0.4096496555906812	T	T	T
0.6413239949823882	0.8333364734430410	0.4112473290445580	T	T	T
0.7821542257729116	0.5000013726027498	0.4096506557604365	T	T	T
0.6413224475586157	0.5000007646070287	0.4112471749449050	T	T	T
0.3573242276940373	0.1666673281976261	0.4090802794242565	T	T	T
0.9216765540786841	0.1666681442893144	0.4088795381844059	T	T	T
0.5006871513679468	0.1666678902055123	0.4103563359783793	T	T	T
0.3573243081132491	0.8333342284039303	0.4090803783091603	T	T	T
0.7821565413606901	0.1666685358971633	0.4096490533379479	T	T	T
0.6413233370050357	0.1666667560004955	0.4112469411264662	T	T	T
0.3573236093195613	0.5000005641857865	0.4090800022514840	T	T	T
0.5006875168054327	0.5000008451316017	0.4103564217959985	T	T	T
0.4298476758778408	0.3333358728452714	0.3427327886406991	T	T	T
0.7125575342961552	0.6666663301207294	0.3432982107980774	T	T	T
0.2852260973135026	0.6666656213127928	0.3437971213595010	T	T	T
0.8576773220896066	0.0000012771808748	0.3418230597204112	T	T	T
0.8576780232186771	0.3333373299950865	0.3418227212342412	T	T	T
0.7125563025576304	0.0000013218498849	0.3432975955054259	T	T	T
0.5711569843247133	0.0000009019666552	0.3432841487981115	T	T	T
0.4298477918696306	0.6666667073363626	0.3427324176844026	T	T	T
0.7125580125616545	0.3333361200735207	0.3432981972494231	T	T	T
0.4298475052064067	0.0000007073997923	0.3427325646608343	T	T	T
0.5711574192027625	0.3333355802835841	0.3432837605480964	T	T	T
0.5711573409760238	0.6666664259998325	0.3432841101999319	T	T	T
0.8576770216916780	0.6666648031069673	0.3418232064266749	T	T	T
0.2852267081436856	0.0000013141722897	0.3437975075237799	T	T	T
0.2852260415987543	0.3333350794894427	0.3437968664204499	T	T	T
0.9333138411239474	0.5000022422059230	0.2799699339047635	T	T	T
0.4995811855434436	0.8333343961309937	0.2762603572216500	T	T	T
0.9333158841061774	0.8333340347795607	0.2799718733225181	T	T	T
0.7862611491905027	0.8333397594490810	0.2775398893186064	T	T	T
0.6425310169900493	0.5000018993305779	0.2767963964747529	T	T	T
0.6425311029648221	0.8333344278086187	0.2767960578146678	T	T	T
0.2131788749941396	0.1666663453779429	0.2790001030083569	T	T	T
0.3570948152437518	0.1666682378521266	0.2775985625888538	T	T	T
0.7862592225582566	0.5000017653464607	0.2775388779456622	T	T	T
0.4995810784132138	0.1666667104995681	0.2762601709567429	T	T	T
0.2131794972069921	0.4999995766901063	0.2789944386173556	T	T	T
0.9333144218899808	0.1666672145078114	0.2799711563066214	T	T	T
0.7862611100635506	0.1666717571371585	0.2775393274042521	T	T	T
0.2131790219389929	0.8333358980730512	0.2790012183925528	T	T	T
0.3570950986723895	0.8333342621902277	0.2775981091066838	T	T	T

0.3570947487815956	0.5000002330549167	0.2775988728214775	T	T	T
0.6425310490930580	0.1666674011110454	0.2767958080691085	T	T	T
0.4995813414473263	0.5000012683114075	0.2762598195215483	T	T	T
0.7155172729518741	0.6666667095674630	0.2107124558448565	T	T	T
0.8602462812930532	0.3333333345915262	0.2121389378222026	T	T	T
0.4271069793772362	0.6666668579750117	0.2102438270674422	T	T	T
0.5723816451644066	0.3333342242507356	0.2104194058904706	T	T	T
0.7155176422113924	0.0000016348214902	0.2107123184338421	T	T	T
0.0031664065617684	0.3333349755773959	0.2140754079625544	T	T	T
0.1404531342048626	0.6666665519028817	0.2144058667267452	T	T	T
0.4271069504742985	0.0000005853544190	0.2102444480006721	T	T	T
0.2840298129677968	0.3333315261375226	0.2113220161771378	T	T	T
0.1404510951156535	0.0000002743320769	0.2144078565061756	T	T	T
0.0031613111733369	0.0000001888638383	0.2140751774133806	T	T	T
0.2840280107836462	0.0000016342147154	0.2113220867337854	T	T	T
0.5723818283212181	0.6666670307157689	0.2104187703531631	T	T	T
0.7155174922263522	0.3333343342773540	0.2107122707970154	T	T	T
0.8602467975975757	-0.0000003067080658	0.2121388586606351	T	T	T
0.2840293737102709	0.6666680758323785	0.2113227137270694	T	T	T
0.4271066712831250	0.3333336138176337	0.2102443096355967	T	T	T
0.8602460613897726	0.6666700059928943	0.2121383075807993	T	T	T
0.5723815497720147	0.0000012823989150	0.2104192933491534	T	T	T
0.0031647445463359	0.6666681436003827	0.2140752056918440	T	T	T
0.1404550018033058	0.3333330864914555	0.2144053996514630	T	T	T
0.9301179774572399	0.1666672909862775	0.1436794057822445	T	T	T
0.4999712972070168	0.1666669799041746	0.1432293524663722	T	T	T
0.0719151253681411	0.5000002984294131	0.1448099941740218	T	T	T
0.7870797352633392	0.5000008727683953	0.1435329472261871	T	T	T
0.2126833621230145	0.1666666052642102	0.1436712378764808	T	T	T
0.6434517266485706	0.8333351012511067	0.1431243311825244	T	T	T
0.9301179580161398	0.8333349363226613	0.1436767984981836	T	T	T
0.3562575029871780	0.8333403925825914	0.1429823816121723	T	T	T
0.3562551331066947	0.5000002827172930	0.1429827681046150	T	T	T
0.4999712958139030	0.5000013117352690	0.1432285542127404	T	T	T
0.6434515721038397	0.1666672840327600	0.1431221628093074	T	T	T
0.2126844744812025	0.8333355252752783	0.1436714886591556	T	T	T
0.0719159578551656	0.8333333329678361	0.1448107376759670	T	T	T
0.7870796196777523	0.1666671263122141	0.1435331701164149	T	T	T
0.2126834661962148	0.5000013775288830	0.1436781774288243	T	T	T
0.3562547748548631	0.1666669565552599	0.1429829628685439	T	T	T
0.6434517570899849	0.5000007715135147	0.1431226870105652	T	T	T
0.0719153036820819	0.1666688145730206	0.1448112439180144	T	T	T
0.7870793193883140	0.8333348465322010	0.1435338817134422	T	T	T
0.4999709621631930	0.8333342467655454	0.1432293823763564	T	T	T

```
0.9301182428507646   0.5000007623477057   0.1436803546054020   T   T   T
0.7142899999999983   0.6666700000000034   0.0773300000000035   F   F   F
0.5714299999999994   0.6666700000000034   0.0773300000000035   F   F   F
0.1428599999999989   0.0000000000000000   0.0773300000000035   F   F   F
0.4285700000000006   0.0000000000000000   0.0773300000000035   F   F   F
0.8571400000000011   0.3333333332999970   0.0773300000000035   F   F   F
0.7142899999999983   0.3333333332999970   0.0773300000000035   F   F   F
0.0000000000000000   0.3333333332999970   0.0773300000000035   F   F   F
0.7142899999999983   0.0000000000000000   0.0773300000000035   F   F   F
0.8571400000000011   0.0000000000000000   0.0773300000000035   F   F   F
0.4285700000000006   0.6666700000000034   0.0773300000000035   F   F   F
0.5714299999999994   0.3333333332999970   0.0773300000000035   F   F   F
0.2857100000000017   0.6666700000000034   0.0773300000000035   F   F   F
0.4285700000000006   0.3333333332999970   0.0773300000000035   F   F   F
0.8571400000000011   0.6666700000000034   0.0773300000000035   F   F   F
0.5714299999999994   0.0000000000000000   0.0773300000000035   F   F   F
0.1428599999999989   0.6666700000000034   0.0773300000000035   F   F   F
0.0000000000000000   0.6666700000000034   0.0773300000000035   F   F   F
0.2857100000000017   0.3333333332999970   0.0773300000000035   F   F   F
0.1428599999999989   0.3333333332999970   0.0773300000000035   F   F   F
0.2857100000000017   0.0000000000000000   0.0773300000000035   F   F   F
0.0000000000000000   0.0000000000000000   0.0773300000000035   F   F   F
```

adsorption：

```
  1.00000000000000
    17.9242999999999988    0.0000000000000000    0.0000000000000000
     0.0000000000000000    7.6818999999999997    0.0000000000000000
     0.0000000000000000    0.0000000000000000   25.8638000000000012
  Cu   C   O   N   H
  123   8   1   1   7
Selective dynamics
Direct
0.5729532084610686   0.6694239117918798   0.4763399864162156   T   T   T
0.8540852395821696   0.3336744862455340   0.4721421200630762   T   T   T
0.7126491060819670   0.0007595289836778   0.4774158176900001   T   T   T
0.5751845962103731   0.3346501143122572   0.4760461393817297   T   T   T
0.4288250107962633   0.3346608572945723   0.4767213343756484   T   T   T
0.8539468870034663   0.0002011542553568   0.4722314146238640   T   T   T
0.5725846287395920   0.0017893639130861   0.4773074524547758   T   T   T
0.7123426051549863   0.6675502178293189   0.4770885523243866   T   T   T
0.4303052511002365   0.0004700987866935   0.4738097494988695   T   T   T
0.4299793620183840   0.6732418391837852   0.4725675210162610   T   T   T
0.7132783589327587   0.3341697413770315   0.4765486138828441   T   T   T
0.8538620310601274   0.6670360866640836   0.4722308187038919   T   T   T
```

0.5014918569264488	0.8365006674303602	0.4099744538499173	T	T	T
0.9238672821558277	0.8337988328767107	0.4059471777969372	T	T	T
0.9239972437877337	0.5004931291622406	0.4058999760769347	T	T	T
0.7832575511200399	0.8339060396187318	0.4090537537499198	T	T	T
0.6426529187155289	0.8355434871388963	0.4105045226320258	T	T	T
0.7836016861581148	0.5008116577330952	0.4088349292209885	T	T	T
0.6426345543970927	0.5021131261426534	0.4101929844821992	T	T	T
0.3577248227585476	0.1701544836712030	0.4095057471777117	T	T	T
0.9238578480215862	0.1670101162772105	0.4059440042897421	T	T	T
0.5003682026675089	0.1704725798283453	0.4105823377222627	T	T	T
0.3564699565082015	0.8368462685247101	0.4092331707742607	T	T	T
0.7835063146433124	0.1668127828523729	0.4088478623451003	T	T	T
0.6425448819782914	0.1667658598590690	0.4104094617388518	T	T	T
0.3576293657589591	0.5014761071204584	0.4092495892884220	T	T	T
0.5007593859685114	0.5006448893623122	0.4103568640919728	T	T	T
0.4295294157953251	0.3350522620108252	0.3431215326347809	T	T	T
0.7133739311666296	0.6678689757599927	0.3425427124671122	T	T	T
0.2848289908923609	0.6680991435638044	0.3438888035459161	T	T	T
0.8574301382302121	0.0004058627313692	0.3403842484123885	T	T	T
0.8576595336002952	0.3338736383554088	0.3404075526419710	T	T	T
0.7133235608446173	0.0005483287854507	0.3423970505575972	T	T	T
0.5715178041634658	0.0018152398778299	0.3431918930060089	T	T	T
0.4294325644483910	0.6687630586160982	0.3431546819570119	T	T	T
0.7134018710633141	0.3338960440686025	0.3426916079746610	T	T	T
0.4294096629647560	0.0018387691003281	0.3428734794122965	T	T	T
0.5716496405528162	0.3346254309804300	0.3431386474411351	T	T	T
0.5716375275904190	0.6685038535495863	0.3431907763148739	T	T	T
0.8575254407154843	0.6672453681414270	0.3403898018593189	T	T	T
0.2849157804961856	0.0023386131164275	0.3438429736047286	T	T	T
0.2846303588219823	0.3348699051053501	0.3438556083930235	T	T	T
0.9335752958259178	0.5003942932271077	0.2791298559886481	T	T	T
0.4991685775119407	0.8343394086792304	0.2768797389522802	T	T	T
0.9334923223143335	0.8337153251368987	0.2789682615706955	T	T	T
0.7857319112198110	0.8338274140766971	0.2763723508361146	T	T	T
0.6423825549050631	0.5010370228113955	0.2767029663253256	T	T	T
0.6423359965768861	0.8342890676307204	0.2762846211182968	T	T	T
0.2127745145639262	0.1674401770495180	0.2788454107351256	T	T	T
0.3565452286623357	0.1680627611108041	0.2777933542103204	T	T	T
0.7857416519722259	0.5004971484144183	0.2765119655129272	T	T	T
0.4991060567360034	0.1681457455315806	0.2769168725783437	T	T	T
0.2128411258283120	0.5007867414327652	0.2789092786135017	T	T	T
0.9335119371295745	0.1670274576654947	0.2790811263764497	T	T	T
0.7857624695675838	0.1673340822908832	0.2764640219736215	T	T	T
0.2126595031864075	0.8341376251745909	0.2789545380014710	T	T	T

0.3564110000447487	0.8344049158548622	0.2778373089057488	T	T	T
0.3565673018040737	0.5012431708376922	0.2778808557221559	T	T	T
0.6424010737899812	0.1673567495579367	0.2765410807492292	T	T	T
0.4990203720737833	0.5011363689747441	0.2770299629844949	T	T	T
0.7147807731408716	0.6669847344480907	0.2099301251641261	T	T	T
0.8601855142036651	0.3336627638130074	0.2118035590810905	T	T	T
0.4264165643042434	0.6671661729868654	0.2108200486153987	T	T	T
0.5715427955094881	0.3341615026119178	0.2106487440451519	T	T	T
0.7148446386170946	0.0006074502016555	0.2098224928226413	T	T	T
0.0028969800878242	0.3334860904653233	0.2128737142419550	T	T	T
0.1400927204601385	0.6670560326347621	0.2142927913829507	T	T	T
0.4264628105565014	0.0003962644542734	0.2108488045544452	T	T	T
0.2834887698713425	0.3337995305230936	0.2113926092659951	T	T	T
0.1401188662231295	0.0004699341985185	0.2142357512907385	T	T	T
0.0029075722070632	0.0002217989197504	0.2128254835734446	T	T	T
0.2835526598294533	0.0003401186844893	0.2114583948180857	T	T	T
0.5713926569882951	0.6671194727208837	0.2106115017604167	T	T	T
0.7147766360384600	0.3338995016257762	0.2100866773898608	T	T	T
0.8601209684809537	0.0004386004091238	0.2117518659636803	T	T	T
0.2834832795366047	0.6670636913891090	0.2114354053342268	T	T	T
0.4265586694830282	0.3339808708973276	0.2108352046320262	T	T	T
0.8601576441447044	0.6668399950303980	0.2117930469325097	T	T	T
0.5714332271758672	0.0006314026039527	0.2106631927251595	T	T	T
0.0029308412137031	0.6666812290783541	0.2128629300313952	T	T	T
0.1400803648008878	0.3338037907223997	0.2143095936311399	T	T	T
0.9297760290624216	0.1667920496417235	0.1433040133909824	T	T	T
0.4993302559184730	0.1670252497297347	0.1434631333474014	T	T	T
0.0721433457724402	0.5001886627823314	0.1443704784578769	T	T	T
0.7868351463659080	0.5001724273813821	0.1433965820055603	T	T	T
0.2126320696170967	0.1667634344551134	0.1437166554962669	T	T	T
0.6426547088463900	0.8336130529961601	0.1430702589443310	T	T	T
0.9298045248386625	0.8333570576018575	0.1433126564870039	T	T	T
0.3559221631161216	0.8334550053137527	0.1431782758768125	T	T	T
0.3559843274596843	0.5000283255225209	0.1432589763121644	T	T	T
0.4993077435973013	0.5000931719468094	0.1433911501567837	T	T	T
0.6427207626778232	0.1670510393824075	0.1430978977430914	T	T	T
0.2126177608938229	0.8334679436056891	0.1437557840593388	T	T	T
0.0721273629364690	0.8334711589350434	0.1443958713074859	T	T	T
0.7868531489266980	0.1670204357782676	0.1433955924138212	T	T	T
0.2125803705861559	0.5001912458004395	0.1437248350584630	T	T	T
0.3560227052298518	0.1668218690344807	0.1432773949291413	T	T	T
0.6427526058428210	0.5001557924192318	0.1430795730919418	T	T	T
0.0721487418544991	0.1667716415408581	0.1443703079725562	T	T	T
0.7869292381929418	0.8334719350841494	0.1434551041778787	T	T	T

0.4992945350579341	0.8335683487212814	0.1433917022749365	T	T	T
0.9297728564000919	0.5000824458845549	0.1433383752908066	T	T	T
0.7142899999999983	0.6666700000000034	0.0773300000000035	F	F	F
0.5714299999999994	0.6666700000000034	0.0773300000000035	F	F	F
0.1428599999999989	0.0000000000000000	0.0773300000000035	F	F	F
0.4285700000000006	0.0000000000000000	0.0773300000000035	F	F	F
0.8571400000000011	0.3333333332999970	0.0773300000000035	F	F	F
0.7142899999999983	0.3333333332999970	0.0773300000000035	F	F	F
0.0000000000000000	0.3333333332999970	0.0773300000000035	F	F	F
0.7142899999999983	0.0000000000000000	0.0773300000000035	F	F	F
0.8571400000000011	0.0000000000000000	0.0773300000000035	F	F	F
0.4285700000000006	0.6666700000000034	0.0773300000000035	F	F	F
0.5714299999999994	0.3333333332999970	0.0773300000000035	F	F	F
0.2857100000000017	0.6666700000000034	0.0773300000000035	F	F	F
0.4285700000000006	0.3333333332999970	0.0773300000000035	F	F	F
0.8571400000000011	0.6666700000000034	0.0773300000000035	F	F	F
0.5714299999999994	0.0000000000000000	0.0773300000000035	F	F	F
0.1428599999999989	0.6666700000000034	0.0773300000000035	F	F	F
0.0000000000000000	0.6666700000000034	0.0773300000000035	F	F	F
0.2857100000000017	0.3333333332999970	0.0773300000000035	F	F	F
0.1428599999999989	0.3333333332999970	0.0773300000000035	F	F	F
0.2857100000000017	0.0000000000000000	0.0773300000000035	F	F	F
0.0000000000000000	0.0000000000000000	0.0773300000000035	F	F	F
0.7414943432924089	0.4405674454591170	0.7488457348104789	T	T	T
0.6515601830395220	0.5383128425054727	0.6868323804170489	T	T	T
0.6027879238934295	0.6721867299142040	0.6707882182735778	T	T	T
0.6470278789199602	0.3716746153706496	0.6643990435877075	T	T	T
0.5474341373008446	0.6376401457199392	0.6350703464273811	T	T	T
0.5920836195094540	0.3369780352198383	0.6281322126482046	T	T	T
0.5405248727654799	0.4680645325876917	0.6142430243528343	T	T	T
0.4828987148400365	0.4250978814411592	0.5796499251435089	T	T	T
0.7012124121067433	0.5807466157203781	0.7242367246921780	T	T	T
0.4374012879132091	0.3819765021263531	0.5494584404426186	T	T	T
0.7733675155281821	0.5013575102570388	0.7800361416869273	T	T	T
0.7019525463807853	0.3452625179094454	0.7648769920289451	T	T	T
0.7795651215572067	0.3763829552314591	0.7215639158146218	T	T	T
0.6082666639196930	0.8009351856986811	0.6879981053779681	T	T	T
0.6858927523295590	0.2691752403915297	0.6751287278136768	T	T	T
0.5087080712285594	0.7393735255163939	0.6233211884425141	T	T	T
0.5883868237790437	0.2087561293822615	0.6104404277492789	T	T	T
